# Rapid prototyping of microbial production strains for the biomanufacture of potential materials monomers

**DOI:** 10.1016/j.ymben.2020.04.008

**Published:** 2020-07

**Authors:** Christopher J. Robinson, Pablo Carbonell, Adrian J. Jervis, Cunyu Yan, Katherine A. Hollywood, Mark S. Dunstan, Andrew Currin, Neil Swainston, Reynard Spiess, Sandra Taylor, Paul Mulherin, Steven Parker, William Rowe, Nicholas E. Matthews, Kirk J. Malone, Rosalind Le Feuvre, Philip Shapira, Perdita Barran, Nicholas J. Turner, Jason Micklefield, Rainer Breitling, Eriko Takano, Nigel S. Scrutton

**Affiliations:** a Manchester Centre for Synthetic Biology of Fine and Speciality Chemicals (SYNBIOCHEM), Manchester Institute of Biotechnology, The University of Manchester, Manchester, M1 7DN, UK; b Manchester Institute of Innovation Research, Alliance Manchester Business School, The University of Manchester, Manchester, M15 6PB, UK; c Department of Chemistry, The University of Manchester, Manchester, M13 9PL, UK

**Keywords:** Synthetic biology, Biofoundry, Biomanufacturing, Industrial biotechnology, Mandelic acid, Hydroxymandelic acid

## Abstract

Bio-based production of industrial chemicals using synthetic biology can provide alternative green routes from renewable resources, allowing for cleaner production processes. To efficiently produce chemicals on-demand through microbial strain engineering, biomanufacturing foundries have developed automated pipelines that are largely compound agnostic in their time to delivery. Here we benchmark the capabilities of a biomanufacturing pipeline to enable rapid prototyping of microbial cell factories for the production of chemically diverse industrially relevant material building blocks. Over 85 days the pipeline was able to produce 17 potential material monomers and key intermediates by combining 160 genetic parts into 115 unique biosynthetic pathways. To explore the scale-up potential of our prototype production strains, we optimized the enantioselective production of mandelic acid and hydroxymandelic acid, achieving gram-scale production in fed-batch fermenters. The high success rate in the rapid design and prototyping of microbially-produced material building blocks reveals the potential role of biofoundries in leading the transition to sustainable materials production.

## Highlights

• An automated biomanufacturing pipeline is benchmarked for the production of material monomers.
• Over 85 days, 160 genes were screened for activity, assembled into 115 unique pathways and tested for *in vivo* production.
• *E. coli* production strains were successfully constructed to produce 17 target compounds at competitive titers.
• Scale-up potential is demonstrated through enantioselective production of mandelic acid targets at gram-scale in bioreactors.
• The high success rate demonstrates the capabilities of biofoundries to rapidly prototype microbial production strains.

## Graphical abstract

**Figure:**
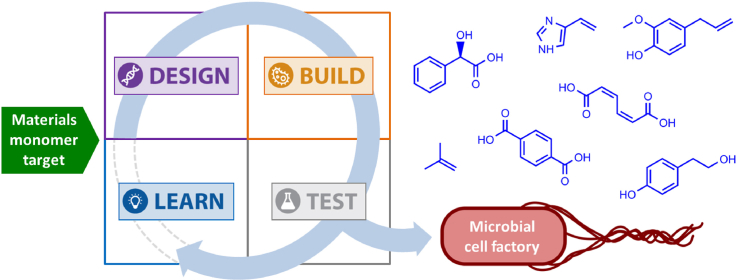

## 1 Introduction

Synthetic biology has expanded our ability to engineer bio-based routes for the production of added-value chemicals, through rational genetic design and metabolic engineering (Choi et al., 2019; Machas et al., 2019; Smanski et al., 2016; Zhang et al., 2019). Progress in synthetic biology automation is leading to the establishment of biofoundry and biorefinery pipelines where the design, selection, robotic assembly and testing of engineered microbial strains is increasingly performed in an automated manner (Appleton et al., 2017; Carbonell et al., 2018a; Chao et al., 2017; Hillson et al., 2019). The current capabilities of biomanufacturing foundries have been demonstrated for several classes of compounds (Casini et al., 2018), including the application of a rapid *Design-Build-Test-Learn* (DBTL) pipeline for iterative optimization of production strains (Carbonell et al., 2018a), and machine learning for the translational tuning of biosynthetic pathways (Jervis et al., 2019). Despite such successes in producing natural added-value chemicals, few bio-based alternatives to established chemical products have reached the market, and much effort has to be dedicated toward increasing bioproduction yield (Clomburg et al., 2017; Davy et al., 2017; Wehrs et al., 2019). The challenge is particularly compelling for compounds, including bio-based material building blocks, which are prevalent in modern daily life but are mainly derived from petrochemicals, with associated environmental and sustainability concerns. Bio-based production using synthetic biology could provide alternative green routes from renewable resources, allowing cleaner production processes by removing the need for harsh solvents and chemicals and by reducing associated waste (Ahmed et al., 2019; Anderson et al., 2018; Kawaguchi et al., 2017; Le Feuvre and Scrutton, 2018; Zhu et al., 2016). Further potential benefits from synthetic biology production approaches include rapid access to chemical diversity and the exploitation of enzymatic specificity, which can tackle chemistry that is difficult or not possible through organic synthesis, such as chiral specificity, regioselectivity or functionalization for improved properties (Wagner et al., 2019).

In this study, we applied a semi-automated synthetic biology pipeline to construct microbial strains capable of producing a range of materials monomers via fermentation of simple carbon sources. Our goal was to benchmark and demonstrate the capabilities of the DBTL approach to provide on-demand biosynthetic routes to chemical building blocks for materials (Fig. S1A). An important goal was to provide a competitive, widely applicable workflow, which is compound agnostic and can be applied to multiple targets in parallel with a short time to delivery, a requirement seldom achieved in synthetic chemistry production processes. The assessment presented here provides precise metrics on the ability of biomanufacturing foundries to rapidly deliver diverse bio-based monomers for sustainable materials applications. To that end, we applied a synthetic biology pipeline comprising state-of-the-art bioproduction pathway design tools, robotized strain engineering, and high-throughput product quantification. The pipeline proceeds iteratively through sequential steps, starting with the identification of a target compound of interest. Retrosynthetic *design* algorithms are used to explore known and putative biochemical routes connecting the target chemical to the metabolism of the host organism. DNA sequences encoding enzymes to catalyze each reaction in the pathway are then designed, synthesized and screened for activity. The build and test steps of the pipeline proceed through an automated platform for genetic pathway assembly, bacterial chassis construction and culture, followed by automated high-throughput screening and quantification of key metabolites and target compounds.

The materials biomanufacturing pipeline was benchmarked based on multiple metrics, including target titers and time to delivery, using *Escherichia coli* as a production strain. The purpose of selecting *E. coli* was to perform rapid prototyping of the bio-based synthetic routes in a well-characterized host, which could later be transferred to other industrial strains for further optimization. We demonstrate how, for several of our targets, the application of our synthetic biology pipeline led to prototype production strains with target titers comparable to those reported for optimized *E. coli* shake-flask cultures (Table 1). Over 85 days we were able to produce 17 potential materials monomers and key intermediates *in vivo*. To explore the scale-up potential of our microbial production strains, we dedicated a second 65-day period to optimizing the production of mandelic acid and hydroxymandelic acid at high enantiopurity. We succeeded in producing 0.8–4.8 g/L of the (*R*)- and (*S*)-enantiomers of both targets from glucose or glycerol in fed-batch fermenter cultures. This study provides a *proof-of-concept* for the promising capabilities of synthetic biology pipelines for the production of materials monomers, and showcases how prototype producer strains can be optimized towards industrially relevant titers and economy-efficient production processes.

**Table 1:** **Summary of material monomer targets ordered by compound class.** Targets are given by their common name, CAS number and three-letter code used in this study. Days to production: days from target selection to first *in vivo* production. Titers are listed as mean values (±standard deviation) for *E. coli* production strains grown at 30 °C for 24 h in TBP media supplemented with 0.4% glycerol. Mandelate titers are for 1-ml scale cultures, prior to optimization and scale-up. Yields are given as mass of product divided by mass of substrate added (glycerol or phenylacrylic acid). The number of gene steps required to produce targets from primary metabolism, the number of enzyme orthologs screened, and the number o pathway constructs screened are presented (see Tables S1–S2). Prior published titers in shake-flask are listed, along with the corresponding references.

Target	Code	CAS	Compound class	Days to production	**Titer (mg/L)**	**Titer (mM)**	**Yield (g/g)**	Genes required	Pathway figure	Enzymes screened	Pathways screened	Published titer in shake-flask (mg/L)	Reference
Cinnamic acid	CIN	140-10-3	Phenylacrylic acids	30	669 ± 59	4.52	0.17	1	Fig. 3A	1	2	650	Bang et al. (2018)
Coumaric acid	COU	501-98-4	Phenylacrylic acids	30	405 ± 64	2.47	0.09	1	Fig. 3A	1	2	974	Kang et al. (2012)
Ferulic acid	FER	537-98-4	Phenylacrylic acids	–	–	–	–	3	Fig. S3A	8	11	196	Kang et al. (2012)
Urocanic acid	URO	3465-72-3	Phenylacrylic acids	40	485 ± 99	3.51	0.12	1	Fig. 3A	5	5	–	–
Styrene	STY	100-42-5	Vinylbenzenes	45	318 ± 3	3.05	0.08	3	Fig. 3A	3	2 + 1	836	McKenna et al. (2015)
4-Vinylphenol	4VP	2628-17-3	Vinylbenzenes	45	26 ± 7	0.22	0.01	3	Fig. 3A	3	2 + 1	355	Kang et al. (2015)
4-Vinylguaiacol	4VG	7786-61-0	Vinylbenzenes	45	a504 ± 24	3.36	0.87	5	Fig. 3A	10	11 + 1	64	Kang et al. (2015)
4-Vinylimidazole	4VI	3718-04-5	Vinylbenzenes	54	17 ± 0	0.18	<0.01	3	Fig. 3A	7	5 + 1	–	–
Coumarol	CMO	20649-40-5	Monolignols	81	25 ± 31	0.17	0.01	4	Fig. 4A	15	2 + 12	a854	Chen et al. (2017)
Coniferol	CFO	32811-40-8	Monolignols	81	a23 ± 2	0.13	0.04	6	Fig. S4A	22	11 + 12	a125	Chen et al. (2017)
Chavicol	CHV	501-92-8	Allylbenzenes	80	a28 ± 7	0.21	0.06	6	Fig. 4A	23	2 + 12+5	–	–
Eugenol	EUG	97-53-0	Allylbenzenes	80	a102 ± 17	0.62	0.18	8	Fig. S4A	30	11 + 12+5	a25	Kim et al. (2014)
Muconic acid	MUC	1119-72-8	Muconic acid	84	31 ± 16	0.22	0.01	5	Fig. S5A	5	1	3153	Thompson et al. (2018)
Butadiene	BUT	106-99-0	Dienes	–	–	–	–	7	Fig. S5A	15	1 + 0	–	–
Tyrosol	TYO	501-94-0	Tyrosol	–	–	–	–	4	Fig. S6A	19	0†	1316	Yang et al. (2018)
(*S*)-Mandelic acid	SMA	17199-29-0	Mandelates	52	170 ± 54	1.12	0.04	1	Fig. 5A	12	13	740	Sun et al. (2011)
(*R*)-Mandelic acid	RMA	611-71-2	Mandelates	68	251 ± 73	1.65	0.06	3	Fig. 5A	17	15	680	Sun et al. (2011)
(*R/S*)-Mandelic acid	XMA	90-64-2	Mandelates	68	220 ± 16/126 ± 25	1.45/0.83	0.09	2	Fig. 5A	13	11	–	–
Phthalic acid	PA	88-99-3	Benzenedicarboxylates	–	–	–	–	5/7	Fig. S7	2	0†	–	–
Isophthalic acid	IPA	121-91-5	Benzenedicarboxylates	–	–	–	–	5/7	Fig. S7	2	0†	–	–
Terephthalic acid	TPA	100-21-0	Benzenedicarboxylates	–	–	–	–	5/7	Fig. S7	2	0†	145	Luo et al. (2017)
Isobutyric acid	IBA	79-31-2	Isobutyl compounds	–	–	–	–	2/3	Fig. S8C	0^b^	0	12,900	Xiong et al. (2015)
Isobutene	IBE	115-11-7	Isobutyl compounds	–	–	–	–	3	Fig. S8C	0^b^	0	–	–
2-Oxoisovaleric acid	OIV	759-05-7	Chassis metabolites	83	2.6 ± 0.7 (>10xWT)§	0.02	–	4	Fig. S8A	3	18	–	–
Phenylalanine	PHE	63-91-2	Chassis metabolites	82	2352 ± 101 (4xWT)§	12.98	–	4	Fig. S2A	4	2	4200	Liu et al. (2018)
Tyrosine	TYR	60-18-4	Chassis metabolites	82	1283 ± 230 (16xWT)§	7.77	<0.01	4	Fig. S2A	4	10	2579	Kim et al. (2018)

a Titers for targets with substrate feeding (3 mM of relevant phenylacrylic acid substrate). † Pathways for these targets were not built.

b MS method not optimized within the benchmarking timeframe. § Chassis engineering was undertaken to boost metabolite precursor levels in *E. coli*, fold titers are also given compared to wildtype (WT).

## 2 Results and discussion

### 2.1 Target selection

**Fig. 1:**
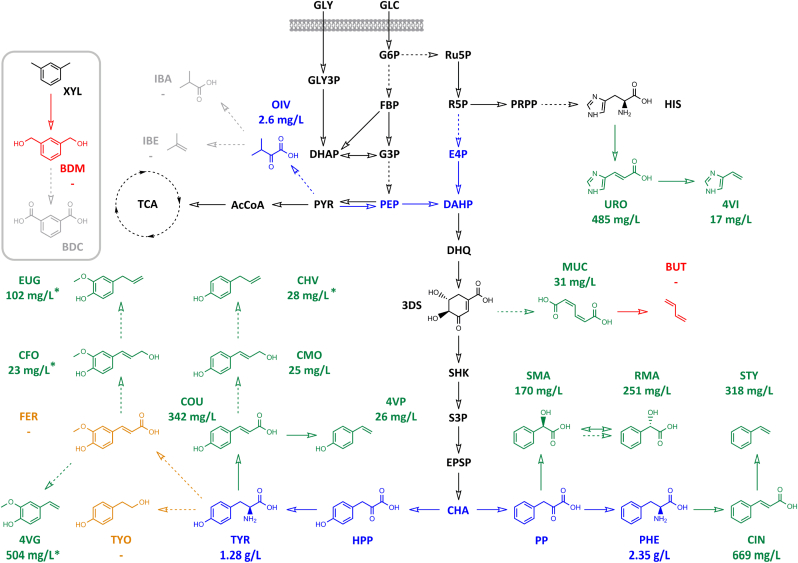
**Biosynthetic routes to material monomer targets**. Target compounds and primary metabolite precursors are represented as skeletal formulae, whilst other *E. coli* primary metabolites are shown as abbreviations. Continuous arrows show single enzyme conversion steps, and dashed arrows indicate multiple enzyme steps. Engineering of the *E. coli* host to boost production of primary metabolite precursors is highlighted in blue. Targets successfully produced *in vivo* are highlighted in green, with growth media titers indicated (asterisks show titers from feeding of phenylacrylic acid precursors). Orange targets were produced enzymatically *in vitro*. Where no active enzyme candidates were identified the targets are red, whilst enzyme steps to gray targets could not be verified within the benchmarking timeframe. Abbreviations used: 3-dehydroshikimate (3DS); 4-vinylguaiacol (4VG); 4-vinylimidazole (4VI); 4-vinylphenol (4VP); acetyl-coenzyme A (AcCoA); benzene dicarboxylate (BDC); benzene dimethanol (BDM); butadiene (BUT); coniferyl alcohol (CFO); chorismate (CHA); chavicol (CHV); cinnamic acid (CIN); coumaryl alcohol (CMO); coumaric acid (COU); 3-deoxy-D-arabinoheptulosonate-7-phosphate (DAHP); dihydroxyacetone phosphate (DHAP); 3-dehydroquinate (DHQ); erythrose-4-phosphate (E4P); 5-enolpyruvylshikimate-3-phosphate (EPSP); eugenol (EUG); fructose-1,6-bisphosphate (FBP); ferulic acid (FER); glucose-3-phosphate (G3P); glucose-6-phosphate (G6P); glucose (GLC); glycerol (GLY); glycerol-3-phosphate (GLY3P); histidine (HIS); 4-hydroxyphenylpyruvate (HPP); isobutyric acid (IBA); isobutene (IBE); muconic acid (MUC); 2-oxoisovaleric acid (OIV); phosphoenolpyruvate (PEP); phenylalanine (PHE); phenylpyruvate (PP); phosphoribosyl-pyrophosphate (PRPP); pyruvate (PYR); ribose-5-phosphate (R5P); (R)-mandelic acid (RMA); ribulose-5-phosphate (Ru5P); shikimate-3-phosphate (S3P); shikimate (SHK); (S)-mandelic acid (SMA); styrene (STY); tricarboxylic acid cycle (TCA); tyrosol (TYO); tyrosine (TYR); urocanic acid (URO); xylene (XYL). (For interpretation of the references to color in this figure legend, the reader is referred to the Web version of this article.)

The first step required selection of the chemical targets for the benchmark set. Selection from the chemical space of industrially relevant monomers was based on targets that are either found in natural biosynthesis routes, or are biosynthetically accessible based on literature information and prospective bio-retrosynthetic research. In total, we selected a list of 25 targets encompassing 7 compound classes that were either materials monomers or advanced precursors, including variants of current materials monomers (Table 1). This chemical diversity includes a broad range of compounds that are not native to the *E. coli* host (Fig. 1), and represents a number of challenges relevant to materials production: vinylbenzene targets were selected due to the chemical diversity accessible through biosynthetic routes and as less-energy intensive, sustainable alternatives to petrochemical equivalents; allylbenzenes form biodegradable polymers with novel routes to production and derivatization; mandelate lactides are used to prepare recyclable polymers, with chiral monomers accessible at high enantiomeric excess through enzymatic catalysis; butadiene is used to produce synthetic rubber, with biorefineries offering the potential to replace petrochemical cracking; isobutyl compounds are monomer targets accessible through clean biosynthesis routes; benzene dicarboxylates (phthalates) are high-volume material targets with prospects for regioselective biosynthesis; and tyrosol can be used for flame-retardant polymers, with fermentation offering simpler and less expensive purification than natural sources. Outside of the 7 primary compound classes, advanced precursor targets included the phenylacrylic acids (vinylbenzene intermediates), monolignols (allylbenzene intermediates) and muconic acid (butadiene intermediate). These targets offer compelling reasons for pursuing alternative synthetic biology solutions to production; however, ongoing assessment and reflection on the anticipated implications of the chosen targets for responsible innovation and sustainability is important.

### 2.2 Design-Build-Test-Learn strategy

**Fig. 2:**
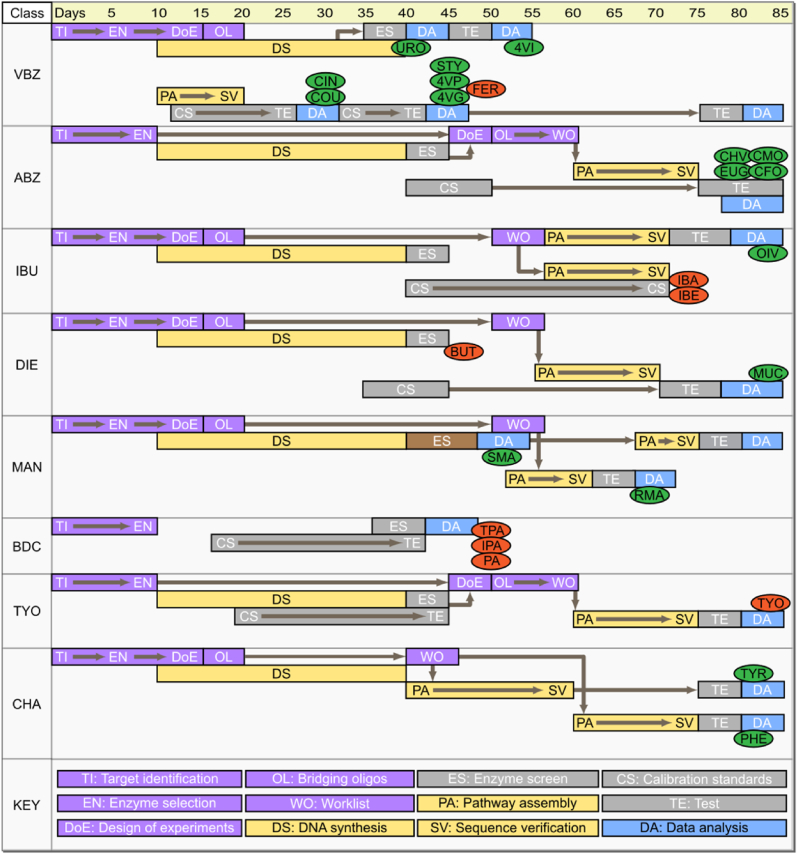
**Gantt chart representing key milestones and their dependencies within the biomanufacturing pipeline**. Steps within the DBTL pipeline are color-coded: Design (purple); Build (yellow); Test (gray); Learn (blue). Abbreviations within ovals represent target compounds and appear at their delivery points (3 letter codes are listed in Table 1). Targets successfully produced *in vivo* are green, red targets were not produced. Compound class abbreviations are: VBZ, vinylbenzene and phenylacrylic acid targets; ABZ, allylbenzene and monolignol targets; IBU, isobutyl compound targets; DIE, butadiene and muconate; MAN, mandelate targets; BDC, benzene dicarboxylate targets; TYO, tyrosol; and CHA, chassis metabolites. BDC targets are: terephthalic acid (TPA), isophthalic acid (IPA) and phthalic acid (PA). (For interpretation of the references to color in this figure legend, the reader is referred to the Web version of this article.)

For each class of chemical target, synthetic metabolic pathways were selected following a semi-automated approach, which involved the use of retrosynthesis software tools such as RetroPath (Delépine et al., 2018), augmented with manual assessment of feasibility and identification of literature precedents. Enzyme candidates for each biosynthetic step were selected following a similar approach, using the computational tool Selenzyme (Carbonell et al., 2018b) and phylogenetic analysis, with final selection considering literature examples of expression and activity in *E. coli* where available. In most cases, where a single ideal enzyme candidate could not be identified, multiple orthologous enzymes were selected for activity screening. In total, 136 gene parts were designed and commercially synthesized (~200 kilobase pairs of DNA; Table S1), coding for enzymes in 25 putative biosynthetic pathways. The design phase, from target and pathway selection through to the design and ordering of DNA parts, took 10 days (Fig. 2). The turnaround time for gene synthesis was 30 days (with genes delivered in batches). Where multiple enzyme candidates were selected from different species to catalyze a given step, the enzymatic activity of these homologs was assessed using a 5-day automated enzyme screening pipeline. In total, 142 unique *in vitro* enzyme assays were performed (including both single and combinatorial cascade reactions), and through this process the best performing homologs (Table S1) were earmarked for assembly into pathway constructs. Larger biosynthetic pathways were designed using a modular approach, with sub-pathways assigned to different vector backbones with compatible replication origins and antibiotic resistance markers. Pathways were split into modules that produced stable and quantifiable intermediates to aid troubleshooting, and allowed us to re-use certain modules where different targets shared common intermediates (Fig. S1B). An optimal *design of experiments* approach was used to combine the selected genes for each pathway/module into a library of expression constructs, with genes arranged in different permutations and with regulatory elements of different strengths (Carbonell et al., 2018a). This approach allowed rapid prototyping of expression construct designs from screening a limited subset of potential combinatorial space, as well as providing robustness against assembly failures given the time constraints. Moreover, while it was not feasible to 'close the loop' of the DBTL cycle within the strict 85-day time limit of this study, our statistical design of experiments was implemented in such a way that we not only identified well-producing prototype strains, but also were able to identify the underlying design rules that can be used to accelerate the next stage of optimization, as illustrated below for the example of mandelic acid production (section 2.9).

Once pathways and enzymes were selected, the construction and screening of target-producing strains followed a semi-automated DBTL pipeline described previously (Carbonell et al., 2018a), but with a number of additional optimizations. The combinatorial library of expression constructs was assembled through an automated approach using liquid handling robots following *in silico* generated worklists (*Supplementary Information - Build*). The resulting DNA constructs were screened first for the correct size by capillary electrophoresis, then subjected to next-generation sequencing to confirm correct assembly (Currin et al., 2019). During the timeframe of this project our *first-pass* assembly efficiency (screening 4–6 clones per assembly) was 29%, with 115 of the 400 planned constructs successfully assembled (Table S2). The sequence-verified expression constructs were then introduced into *E. coli* host strains for monitoring of chemical target production by mass spectrometry (MS; 11 bespoke methods established to quantify 40 target analytes; Table S3). To further boost the titers of certain target compounds, 9 mutant bacterial strains were engineered to overproduce pathway precursors (tyrosine, phenylalanine and 2-oxoisovalerate; Table S4) through a combination of targeted gene knockouts and overexpression of native genes including feedback-resistant mutations. In total, the whole process took 85 working days to perform in parallel for all the 25 material target projects (Fig. 2).

### 2.3 Chassis engineering for phenylalanine/tyrosine overproduction

A majority of the materials monomer targets are accessible through pathways that branch off from the native shikimate pathway for aromatic amino acid biosynthesis (Fig. S2A). To enhance titers for these targets we undertook chassis engineering of *E. coli* strains (DH5α and MG1655) to boost production of phenylalanine and tyrosine (Rodriguez et al., 2014). First, we deleted the *tyrR* gene (a transcriptional regulator of aromatic amino acid biosynthesis) from the host cell genome, followed by deletion of either the *pheA* or *tyrA* genes (chorismate mutase/prephenate dehydratase (CMPDH) for phenylalanine and tyrosine biosynthesis, respectively). For pathways with aromatic aldehyde intermediates we also deleted the *feaB* gene (phenylacetaldehyde dehydrogenase). These knockout strains were then used as hosts for plasmids carrying a number of genes to channel carbon into the shikimate pathway. Transketolase (*tktA* gene) and phosphoenolpyruvate synthase (*ppsA*) both boost availability of shikimate pathway substrates, whereas 3-deoxy-D-arabinoheptulosonate 7-phosphate synthase (DAHPS; *aroF, aroG*) and CMPDH (*pheA, tyrA*) exist as amino acid specific isoforms that are feedback regulated. We incorporated mutations into our DAHPS and CMPDH genes to relieve feedback inhibition (denoted with a * (Kikuchi et al., 1997; Lütke-Eversloh and Stephanopoulos, 2005; Nelms et al., 1992; Weaver and Herrmann, 1990)).

To boost tyrosine production, we designed a plasmid library containing the tktA, ppsA, aroG* and tyrA* genes. However, the *tktA* gene consistently failed to assemble into these constructs; therefore, we screened a panel of ten 3-gene plasmids without this gene (Fig. S2B, Table S2). In the Δ*tyrR,* Δ*pheLA* double knockout strain one plasmid clearly outperformed the rest (SBC005753), producing 1.28 g/L of tyrosine, representing a 16-fold improvement over the wildtype strain with no plasmid (0.08 g/L). To boost phenylalanine production, we designed a separate plasmid library containing tktA, ppsA, aroF* and pheA* genes, and noticed similar difficulties in assembling *tktA* into our constructs, however in this instance we identified two complete 4-gene plasmids (SBC008290 and SBC008376, Table S2). The two plasmids were screened in the Δ*tyrR,* Δ*tyrA* double knockout strain (Fig. S2C), producing 2.35 g/L and 1.96 g/L of phenylalanine respectively, representing a 3.9- and 3.2-fold improvement over the wildtype without plasmid (0.61 g/L). These engineered host strains, transformed with amino acid boost plasmids (Fig. S2D), were used as described with target production plasmids to measure final target titers. For mandelic acid targets, later optimization work was done with double knockout strains in which the amino acid boost constructs were integrated into the genome, to relieve the burden on cells to maintain multiple plasmids in the presence of multiple antibiotics.

### 2.4 Production of phenylacrylic acid targets

**Fig. 3:**
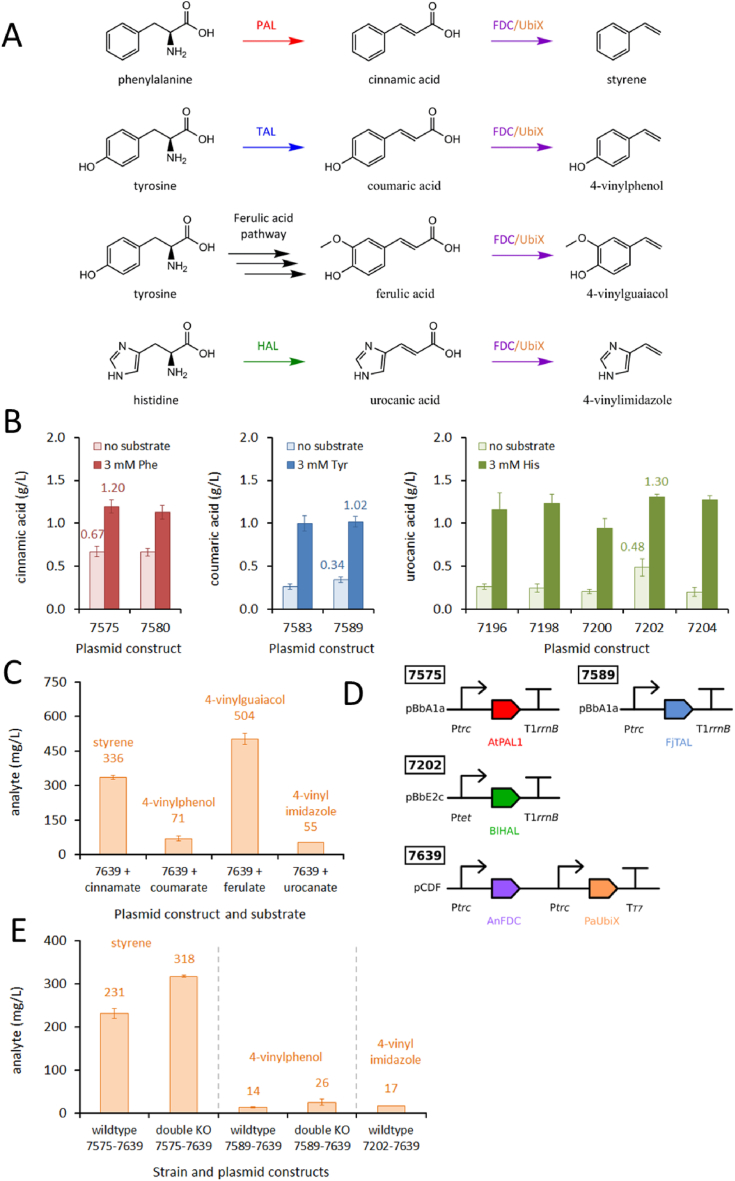
(**A**) Pathways from amino acid substrates to corresponding *trans*-phenylacrylic acid and vinylbenzene/vinylimidazole targets. Enzyme abbreviations: PAL (phenylalanine ammonia-lyase); TAL (tyrosine ammonia-lyase); HAL (histidine ammonia-lyase); FDC (ferulic acid decarboxylase); and UbiX (flavin prenyltransferase). (**B**) Screening of ammonia-lyase candidates. Enzymes were expressed in DH5α cells with or without 3 mM amino acid feed. AtPAL (*Arabidopsis thaliana*) expressed from pBbA1a (SBC007575) and pBbA5a (SBC007580) plasmids. FjTAL (*Flavobacterium johnsoniae*) expressed from pBbA1a (SBC007583) and pBbA5a (SBC007589) plasmids. HAL candidates expressed from the pBbE2c plasmid, from which SBC007202 (BlHAL, *Burkholderia lata*) was selected. (**C**) Screening AnFDC (*Aspergillus niger*) activity with PaUbiX (*Pseudomonas aeruginosa*) and various substrates. Both enzymes were expressed from Ptrc promoters on a pCDF plasmid in DH5α cells with 3 mM substrate feed. (**D**) Best performing plasmid constructs. (**E**) *In vivo* production of targets. DH5α wildtype and double knockout (*ΔtyrR,ΔtyrA* (styrene) or *ΔtyrR,ΔpheLA* (vinylphenol)) strains transformed with indicated plasmids and assayed after 24 h.

Phenylacrylic acids are intermediates along the pathways to our vinylbenzene and allylbenzene targets (Fig. 3A), but are also themselves interesting materials monomer targets. Cinnamic acid can be polymerized through its double bond to produce acrylic resins with high thermal stability (Imada et al., 2019), or it can be co-polymerized with styrene or methyl acrylate (Terao et al., 2019). In contrast, coumaric acid and ferulic acid can be polymerized through their carboxylate and hydroxyl moieties to form polyester materials that are cytocompatible for biomedical applications (Thi et al., 2008). Given these potential materials uses of phenylacrylic acids, and since these compounds are intermediates towards several other monomer targets, we expressed the gene(s) for their biosynthesis from separate plasmid modules.

Phenylalanine ammonia-lyase (AtPAL1, from *Arabidopsis thaliana*) and tyrosine ammonia-lyase (FjTAL, from *Flavobacterium johnsoniae*) were chosen on the basis of our prior work with these enzymes (Carbonell et al., 2018a), and were expressed from *trc* or *lacUV5* promoters (Fig. 3B). Both promoters yielded similar final titers of product, so we selected the P*trc* plasmids for both ammonia-lyase genes. The SBC007575 (AtPAL1) plasmid yielded 0.67 g/L (4.5 mM) of cinnamic acid when expressed in wildtype DH5α cells, or 1.2 g/L (8.1 mM) when media was supplemented with 3 mM phenylalanine; whereas SBC007575 (FjTAL) yielded 0.34 g/L (2.1 mM) coumaric acid, or 1.02 g/L (6.2 mM) when supplemented with 3 mM tyrosine. *E. coli* strains have been reported to produce 650 mg/L cinnamic acid (Bang et al., 2018) and 974 mg/L coumaric acid (Kang et al., 2012); however, these titers are for shake-flask cultures fed considerable quantities of glucose, so our 1-ml scale cultures compared favorably. For urocanic acid production, we selected 5 histidine ammonia-lyase (HAL) homologs for gene synthesis (Table S1) and screened their activity in wildtype DH5α cells (Fig. 3B). All five HAL plasmids were functional, although SBC007202 (HAL from *Burkholderia lata*) was the most productive, yielding 0.48 g/L (3.5 mM) urocanic acid without media supplementation and 1.3 g/L (9.4 mM) with 3 mM histidine. We did not find prior literature reports for optimization of urocanic acid production in *E. coli*.

We attempted to construct a 3-gene pathway to ferulic acid using genes available in house (Fig. S3A). This pathway progresses from tyrosine to L-DOPA (tyrosinase, TYR), then through caffeic acid (TAL from *Herpetosiphon aurantiacus*) to ferulic acid (caffeate *O*-methyl transferase, COMT). We designed a 12-member DoE library of ferulic acid plasmids and successfully constructed 11 (Table S2). These plasmids were tested in wildtype DH5α cells and although they produced coumaric acid and L-DOPA, there was little detectable caffeic acid and no ferulic acid (Fig. S3B). We subsequently tested the *in vitro* activity of our TYR and COMT enzymes, alongside a panel of homologs (Fig. S3D; Table S1), and found that our COMT (MsCOMT1, *Mentha spicata*) had poor activity with both L-DOPA and caffeic acid as substrates. Another issue with this pathway is likely to come from competition for tyrosine between TYR and TAL, generating coumaric acid which tyrosinase cannot process. In redesigning this pathway, we would seek to create a sequential route (Fig. S3A), by replacing TYR with a coumarate 3-hydroxylase (Berner et al., 2006) and expressing this alongside HaTAL and the best performing COMT enzyme (PkCOMT1, *Populus kitakamiensis*).

### 2.5 Production of vinylbenzene targets

Polystyrene is one of the most widely produced plastics, commonly used for protective packaging, food packaging and disposable cups/cutlery. It is produced by free-radical catalyzed polymerization of styrene through its vinyl moiety. 4-Vinylphenol (4VP) and 4-vinylguaiacol (4VG) are styrene derivatives that polymerize in a similar manner, provided the hydroxyl moieties are first protected (Barclay et al., 1998; Takeshima et al., 2017). Poly(4-vinylphenol) is used in electronics applications due to its dielectric and photoresist properties, whilst poly(4-vinylguaiacol) is antimicrobial and potentially biodegradable (Hatakeyama et al., 1977). 4-vinylimidazole (4VI) is another styrene analog that can form polymers with potentially novel properties, such as the absorbance of heavy metal ions (Rivas et al., 1998). Styrene and its derivatives are produced industrially from the unsustainable use of petrochemical feedstocks (Cavani and Trifir, 1995). The decarboxylation of *trans*-phenylacrylic acids offers an alternative production process from a renewable resource, as these compounds are precursors in the biosynthesis of lignin by plants (Boerjan et al., 2003).

We planned to produce vinylbenzene targets through a 2-step pathway (Fig. 3A). Amino acid substrates would first be converted to *trans*-phenylacrylic acids by ammonia-lyases, followed by decarboxylation by ferulic acid decarboxylase (FDC). FDC requires a prenylated flavin cofactor (PrFMN), and so to support enhanced production of this cofactor the enzyme UbiX was also included. We expressed the gene(s) for biosynthesis of phenylacrylic acids from separate plasmids (section 2.4) to the FDC and UbiX genes. Replication origins and antibiotic resistance were selected to ensure compatibility of ammonia-lyase plasmids with amino acid overexpression plasmids, as well as with vinylbenzene and allylbenzene constructs (Table S2). We were able to construct most of these plasmids immediately from genes already in house.

To produce the vinylbenzene targets, from *trans*-phenylacrylic acid precursors, we modified a plasmid carrying AnFDC (*Aspergillus niger*) and PaUbiX (*Pseudomonas aeruginosa*), kindly provided by a colleague (Prof. David Leys). We tested this plasmid (SBC007639) in wildtype DH5α cells grown in media supplemented with 3 mM of phenylacrylic acid precursors (Fig. 3C). We grew these cultures in gas-tight glass vessels and overlaid culture media with 50% v/v isooctane to capture the volatile products. With this set-up we successfully produced all four targets *in vivo*: 336 mg/L styrene; 71 mg/L 4VP; 504 mg/L 4VG; and 55 mg/L 4VI. Finally, to express full biosynthetic pathways we dual-transformed wildtype and double knockout strains of DH5α with ammonia-lyase and FDC plasmids (Fig. 3D). We measured our highest titer of styrene (318 mg/L) in the Δ*tyrR*, Δ*tyrA* strain and highest 4VP (26 mg/L) in the Δ*tyrR*, Δ*pheLA* strain (Fig. 3E). The highest literature titers for *E. coli* production of vinylbenzenes are from bioreactor cultures fed high quantities of glucose with gas-stripping of products; however, for shake-flask cultures, titers of 836 mg/L styrene, 355 mg/L 4VP and 64 mg/L 4VG have been reported (Kang et al., 2012; McKenna et al., 2015). Titers for our prototype production strains compare favorably to the literature, especially since they were obtained at 1-ml scale with just 0.4% glycerol. Our highest titer of 4VI without urocanic acid feeding was 17 mg/L in wildtype DH5α (Fig. 3E). To our knowledge this is the first time 4VI has been produced in *E. coli*. With further time we would next combine ammonia-lyase genes with FDC and UbiX into full pathway constructs, these plasmids could then be co-transformed into our double knockout strains along with amino acid boost plasmids (Fig. S2D). Vinylbenzenes are quite toxic to *E. coli* cells (styrene toxicity threshold is ~300 mg/L for *E. coli* (McKenna et al., 2015)), so further improvements in production might require tolerance engineering or *in situ* product removal, for example by gas-stripping from bioreactor cultures.

### 2.6 Production of allylbenzene and monolignol targets

Lignin is a polymer component of plant cell walls, conferring mechanical strength to plant tissues and facilitating water-transport. Monolignols are the monomer precursors in lignin biosynthesis, and are crosslinked into a complex branched polymer structure (Boerjan et al., 2003). The three common monolignols are coumaryl alcohol (coumarol), coniferyl alcohol (coniferol) and sinapyl alcohol; and there is significant interest in using these monomers to produce synthetic lignin mimics for materials applications (Ganewatta et al., 2019; Önnerud et al., 2002). Monolignols are difficult to purify from plant materials, and so microbial production of these valuable compounds has been investigated (Chen et al., 2017; Jansen et al., 2014). As well as their role in lignin biosynthesis, monolignols are also precursors in the biosynthesis of the allylbenzenes chavicol and eugenol, volatile compounds used as flavors and fragrances. Allylbenzenes resemble vinylbenzenes, with terminal double bonds for polymerization; however, most materials uses seek to polymerize through functionalization of the phenyl hydroxyl group, to preserve the allyl moiety for its antimicrobial properties (Kaufman, 2015). For example, eugenyl methacrylate and ethoxyeugenyl methacrylate monomers are simple to prepare (Rojo et al., 2006), and have been polymerized into oil-absorbent microspheres (Deng et al., 2015). Whilst chavicol-based benzoxazine monomers can been polymerized into thermoset resins with adjustable thermo-mechanical properties through controlled crosslinking (Dumas et al., 2016). Allylbenzenes have been produced from plant genes expressed in *E. coli*, but only from feeding of their respective monolignol precursors (Kim et al., 2014). Therefore, we selected chavicol and eugenol, along with their precursors coumarol and coniferol, as targets for production.

**Fig. 4:**
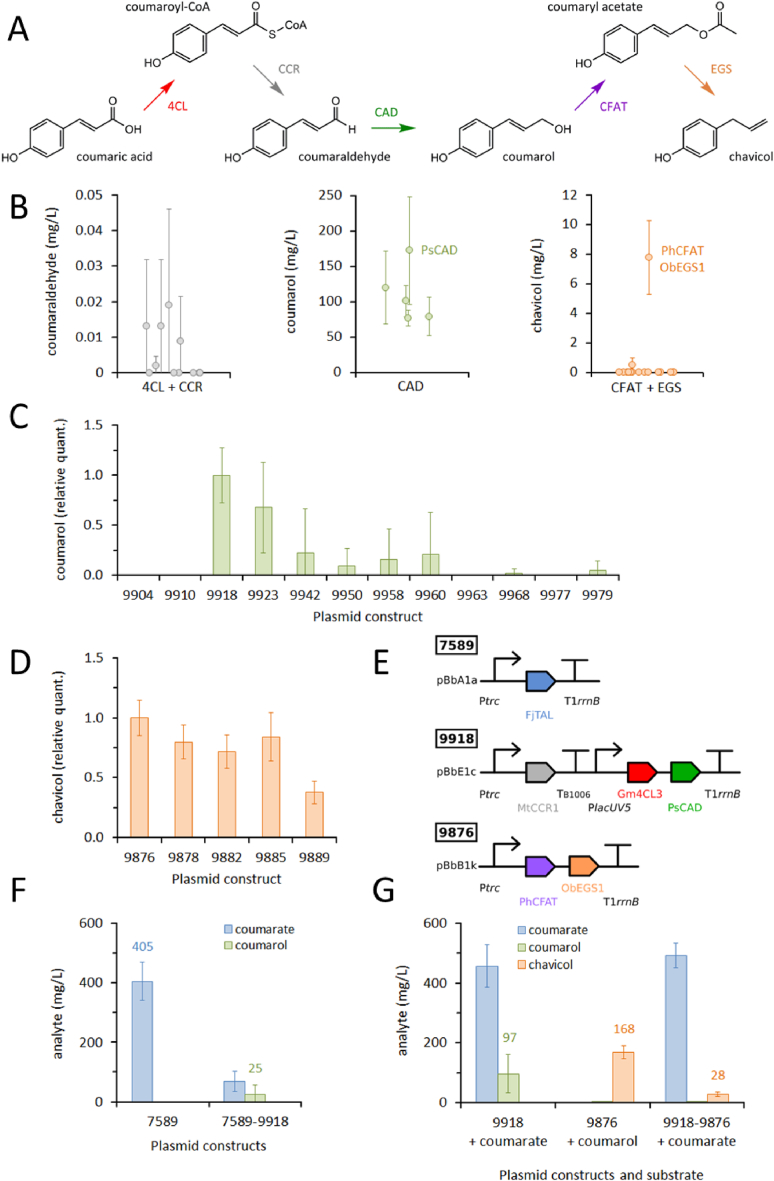
(**A**) Pathway from coumarate to coumarol and chavicol. Enzyme abbreviations: 4CL (4-coumarate-CoA ligase); CCR (cinnamoyl-CoA reductase); CAD (cinnamyl alcohol dehydrogenase); CFAT (coniferyl alcohol acyltransferase); and EGS (eugenol synthase). (**B**) Screening enzyme candidates in cell lysates with 3 mM substrate. No enzyme candidates were selected from the paired 4CL + CCR screen fed coumarate. PsCAD (*Pseudomonas* strain HR199) was selected to convert coumaraldehyde to coumarol. PhCFAT (*Petunia hybrida*) and OcEGS1 (*Ocimum basilicum*) were selected for converting coumarol to chavicol. (**C**) Screening coumarol pathway constructs in DH5α cells fed 3 mM coumarate. After 24 h, SBC009918 was the most productive plasmid. (**D**) Screening chavicol pathway constructs in DH5α cells fed 3 mM coumarol. After 24 h, SBC009876 was the most productive plasmid. (**E**) Best performing plasmid constructs. SBC007589 was screened in Fig. 3. MtCCR1 (CCR from *Medicago truncatula*), Gm4CL3 (4CL from *Glycine max*). (**F**) *In vivo* production of coumarate and coumarol. DH5α (*ΔtyrR ΔpheLA*) cultures with the indicated plasmids were assayed after 24 h. (**G**) *In vivo* production of coumarol and chavicol. DH5α (*ΔtyrR ΔpheLA*) cultures with the indicated plasmids were fed 3 mM coumarate or coumarol and assayed after 24 h.

Coumaric acid and ferulic acid can be produced in *E. coli* through pathways already described (section 2.4). These *trans*-phenylacrylic acids can then be activated as CoA thioesters by 4-coumarate-CoA ligase (4CL), and reduced twice by cinnamoyl-CoA reductase (CCR) and cinnamyl-alcohol dehydrogenase (CAD) to yield coumarol or coniferol (Figs. 4A and S4A). Allylbenzenes can then be reached though acetylation by coniferyl alcohol acyltransferase (CFAT) and reduction by eugenol synthase (EGS). We selected 3–6 enzyme homologs for each step in this pathway (Table S1) for *in vitro* enzyme screening (Figs. 4B and S4B). We could not identify functional 4CL and CCR candidates in a paired assay, presumably due to instability of the aldehyde products. For the remaining steps, PsCAD (*Pseudomonas sp.* strain HR199), PhCFAT (*Petunia hybrida*) and ObEGS (*Ocimum basilicum*) were the best performing candidates for both the chavicol and eugenol targets. A library of plasmid constructs was designed to express PsCAD with Gm4CL3 (Glycine max), which we knew to be functional from previous studies (Carbonell et al., 2018a), and all six CCR gene candidates. From this library we successfully constructed 12 plasmids (Table S2), and screened *E. coli* DH5α transformants for monolignol production with feeding of phenylacrylic acid substrates (Figs. 4C and S4C). The best performing plasmid for coumarol (SBC009918) produced relatively little coniferol, and the best plasmid for coniferol (SBC009968) likewise produced little coumarol. These plasmids differ in the source of their genes for CCR (MtCCR1, *Medicago truncatula* for coumarol; PhCCR1 *P. hybrida* for coniferol), indicating that these enzymes discriminate between the two corresponding CoA thioesters (Figs. 4A and S4A). A second plasmid library was designed to express PhCFAT and ObEGS1, to convert monolignols into allylbenzene targets. We constructed 5 plasmid variants (Table S2) and tested DH5α transformants for production of allylbenzene targets with monolignol feeding (Figs. 4D and S4D). For this library, a single plasmid (SBC009876) performed best at producing both chavicol and eugenol.

Combining the coumarol pathway plasmid (Fig. 4E; SBC009918) with the TAL plasmid (Fig. 3D; SBC007589) should allow for production of coumarol from tyrosine. The DH5α (Δ*tyrR* Δ*pheLA*) double knockout strain was transformed with the TAL plasmid alone, or in combination with the coumarol plasmid (Fig. 4F). In this strain, the TAL plasmid produced 405 mg/L of coumaric acid after 24 h culture in TBP media with 0.4% glycerol. This was a modest improvement over the 342 mg/L observed for this plasmid in wildtype DH5α cells (Fig. 3B). The double knockout strain with both the TAL and coumarol plasmids, produced 25 mg/L coumarol (Fig. 4F). To expand the pathway to chavicol, the double knockout strain was transformed with the coumarol and CFAT/EGS (SBC009876) plasmids, both together and singularly (Fig. 4G). The coumarol plasmid transformants yielded 97 mg/L of coumarol in media supplemented with 3 mM coumaric acid, however most of the coumaric acid substrate remained unconverted. Cells transformed with just the CFAT/EGS plasmid yielded 168 mg/L chavicol in media supplemented with 3 mM coumarol. In this case, whilst the substrate was not detectable after 24 h, chavicol titers were below the theoretical maximum of 403 mg/L (3 mM). It is possible that the substrate was converted to other side products by these cells, or alternatively the volatile chavicol product may have been lost from the media. When double knockout cells were transformed with both the coumarol and CFAT/EGS plasmids, 28 mg/L of chavicol was produced from the coumaric acid substrate (Fig. 4G).

Similar experiments were conducted for the coniferol/eugenol targets (Fig. S4E); however, in this case no pathway to ferulic acid was available to connect to primary metabolism. Double knockout cells transformed with the coniferol plasmid (Fig. S4F; SBC009968) yielded 23 mg/L coniferol from 3 mM ferulic acid substrate, whilst those transformed with the CFAT/EGS plasmid yielded 28 mg/L eugenol from 3 mM coniferol. Combining both plasmids in the double knockout strain gave our highest titer of 102 mg/L eugenol after 24 h culture, but with a significant amount of the ferulic acid substrate left unconverted (Fig. S4E). To our knowledge this is the first time chavicol and eugenol have been produced microbially from phenylacrylic acid substrates. Coumarol and coniferol have been produced in engineered *E. coli* cells before, yielding 502 mg/L and 125 mg/L respectively in shake-flask cultures (Chen, 2017). Given further time we would seek to consolidate the genes for coumarol/coniferol and chavicol/eugenol biosynthesis into single plasmid constructs, allowing production of these targets directly from tyrosine produced by the *E. coli* host. We would then further transform these cells with our tyrosine boost plasmid (Fig. S2D; SBC005753) to channel carbon flux towards production of the targets. Similarities in structure between the allylbenzenes and styrene suggest that these targets may also be toxic and volatile, therefore further improvements in yield might be achieved through including an organic overlay or gas-stripping from bioreactor fermentations.

### 2.7 Additional material monomer targets

Butadiene is an important commodity chemical as a monomer for the production of synthetic rubber. The FDC enzyme has documented activity for converting sorbic acid into 1,3-pentadiene (Aleku et al., 2018), so we considered the potential for this enzyme to produce butadiene from muconic acid. For this purpose, we planned to construct a muconic acid pathway to support the potential *in vivo* production of butadiene. Muconic acid is also a materials target, which can be used as monomer for producing bio-based polyacrylate mimics (Quintens et al., 2019) and is a valuable platform chemical for the production of nylon, polyurethane and PET monomers. Muconic acid production by *E. coli* has been thoroughly explored, with a titer of 3.1 g/L reported for a ‘metabolic funnel’ pathway which converts the metabolites 3-dehydroshikimate (3DS) and chorismate into protocatechuic acid, then onto *cis,cis*-isomer of muconic acid (Thompson et al., 2018). Since it would be difficult to improve upon this highly optimized pathway (Fig. S5A), we constructed a 5-gene plasmid encoding the same set of enzymes (Fig. S5B). This pathway was tested in a range of wildtype and knockout host strains (Fig. S5C), with a highest observed titer of 31 mg/L muconic acid (138 mg/L with 3DS substrate feeding). Although this was significantly less than the titer reported by Thompson et al., that study was conducted in shake-flasks with optimized media supplemented with 20 g/L glucose. We *in vitro* tested a panel a five different FDC homologs in combination with five UbiX homologs, for their ability to convert *cis,cis-* or *trans-,trans-*muconic acid into butadiene, but failed to detect this product. We also failed to observe butadiene production from muconic acid fed to *E. coli* cells carrying the functional FDC/UbiX plasmid (SBC007639), despite these cells producing 1,3-pentadiene from sorbic acid. Future projects to produce butadiene might consider screening a wider library of FDC homologs or undertaking mutagenesis of the active site to alter the substrate tolerance of this enzyme.

Tyrosol is an antioxidant compound found naturally in olive oil, that can be polymerized into flame-retardant plastics (Bouldin et al., 2017). Tyrosol can be synthesized in *E. coli* by expressing phenylacetaldehyde synthase (PAAS) to convert tyrosine into 4-hydroxyphenylacetaldehyde (HPAA), which is then reduced by native aldehyde dehydrogenases (ADHs) into tyrosol (Chung et al., 2017). Alternatively, tyrosine decarboxylase (TDC) can produce tyramine, which is acted upon by monoamine oxidase (MAO) enzyme to produce HPAA (Satoh et al., 2012). We aimed to improve on these studies by combining both routes in a 4-gene pathway (Fig. S6A), including an exogenous ADH gene. We *in vitro* screened 6 enzyme homologs each for the PAAS, TDC and ADH enzymes, and 3 for MAO (Fig. S6B; Table S1). Whilst we discovered enzymes with good activity for all steps, the best performing homologs were those previously identified (Chung et al., 2017; Satoh et al., 2012). For this reason, and due to time constraints, we did not continue work on tyrosol.

The benzene dicarboxylate terephthalic acid (TPA) is a monomer for polyethylene terephthalate (PET), the most widely produced plastic in the world, used to make clothing and food/drink containers. Isophthalic acid (IPA) and phthalic acid (PA) are isomers of TPA, and also commodity chemicals used to make plastics, often as copolymers with TPA. *E. coli* has been used for whole-cell biotransformation of *p*-xylene into TPA, using a 6-step (8 gene) pathway in a two-phase partitioning fermentation to achieve 96.7% conversion yield (Luo and Lee, 2017). For this study we were interested in whether we could produce a shorter route to TPA, and whether we could produce IPA or PA from similar enzymes. A patented xylene monooxygenase (XMO, XylMA heterodimer) from *Sphingomonas* is reported to oxidize both methyl moieties of *p*- or *m*-xylenes in the production of hydroxymethyl benzoates (Bramucci et al., 2003). We tested the ability of this enzyme, alongside the literature *Pseudomonas putida* XMO, to convert *o*-, *m*- or *p*-xylene into their respective methylbenzyl alcohol or benzenedimethanol products (Fig. S7). However, using our *in vitro* enzyme assays we were unable to detect these products by MS from either XMO candidate. Given the lack of evidence for XMO functionality, we decided not to pursue the *in vivo* production of benzene dicarboxylates in favor of other targets.

2-oxoisovaleric acid (OIV) is an *E. coli* metabolite intermediate in the synthesis of valine, leucine and coenzyme A (CoA). Decarboxylation of OIV can be exploited for the synthesis of a variety of isobutyl compounds, including our targets isobutene and isobutyric acid. To boost OIV production we deleted the *ilvE* gene of DH5α, and designed plasmid constructs to overexpress four genes in the valine biosynthesis pathway (Fig. S8A). We screened a library of these constructs in the *ΔilvE* strain and identified plasmid SBC008111 (Fig. S8B) as the best performing construct, producing 2.61 mg/L of OIV. Although this was a modest level of production, OIV was undetectable in the wildtype strain without plasmid, and so this engineered chassis was selected to host pathways to isobutyl compound targets. Isobutene is a monomer for polyisobutylene (butyl rubber), widely-used for its gas-impermeability, whilst isobutyrate is an important precursor for the synthesis of the methyl methacrylate monomer of poly(methyl methacrylate) (PMMA, Perspex). We designed a biosynthetic pathway to isobutene following an established route to isobutanol ((Atsumi et al., 2008); Fig. S8C). Decarboxylation of OIV by 2-keto acid decarboxylase (KDC) produces isobutyraldehyde which is then reduced by the native *E. coli* alcohol dehydrogenase (ADH). Isobutanol could then be converted into isobutene by oleate hydratase (OHD (Marliere, 2011)). We selected 6 enzyme homologs for KDC and 9 for OHD (Table S1) for *in vitro* enzyme screening. Isobutyric acid can also be produced in *E. coli* through the action of KDC followed by oxidation, provided several native aldehyde reductases are deleted (Xiong et al., 2015). We selected an alternative route through an acyl-CoA intermediate, using 2-oxoisovalerate dehydrogenase (ODH) and acyl-CoA ligase (ACL, Fig. S8C). For this pathway we designed a plasmid library and successfully constructed 7 pathway variants (Table S2) for screening in our engineered host. Methods for GC-MS detection of isobutene, isobutyric acid and various intermediates were developed, but the sensitivity of detection dropped significantly in culture medium, which meant these compounds could not be reliably quantified in enzyme and pathway screens. Therefore, further progress on these targets was not possible in the limited timeframe we had set ourselves.

### 2.8 Production of mandelic acid enantiomers

**Fig. 5:**
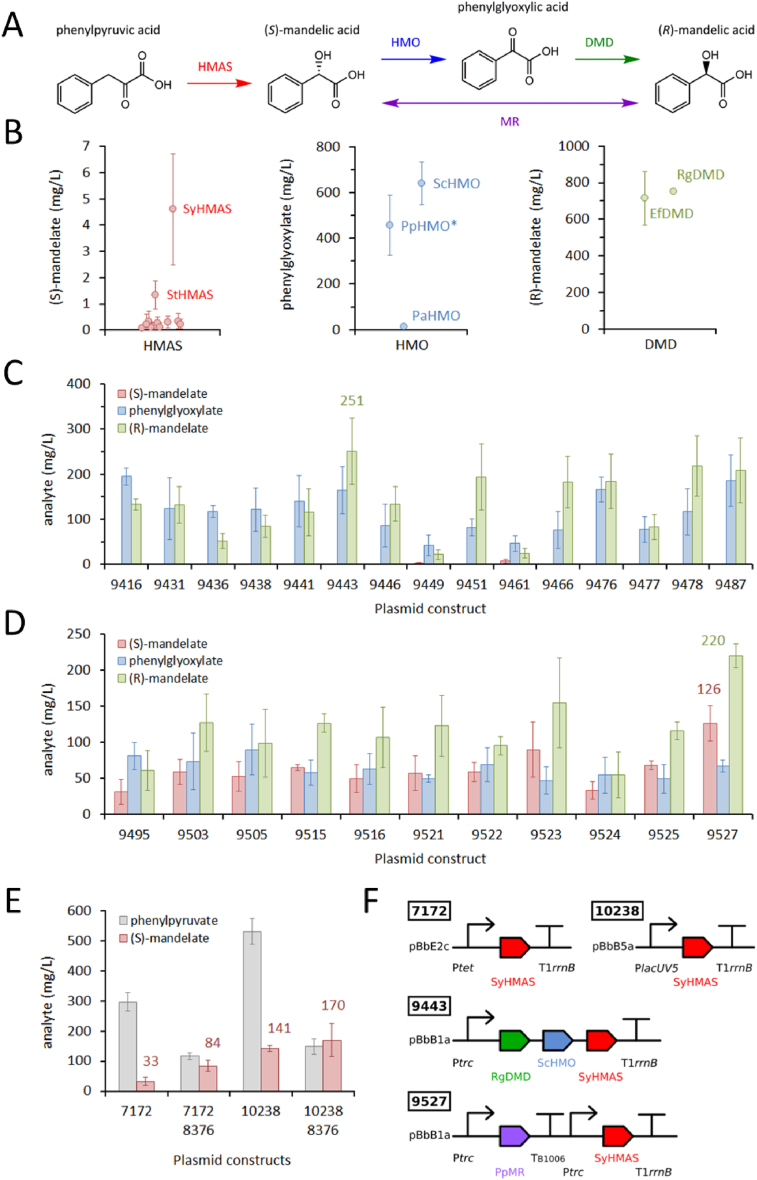
(**A**) Pathways from phenylpyruvate to the (*S*)- and (*R*)-enantiomers of mandelate. Enzyme abbreviations: HMAS (hydroxymandelate synthase); HMO (hydroxymandelate oxidase); DMD (D-mandelate dehydrogenase); and MR (mandelate racemase). (**B**) Screening enzyme candidates. HMAS candidates expressed from the pBbE2c plasmid in DH10β cells fed 3 mM phenylpyruvate. SyHMAS (*Streptomyces yokosukanensis*) was the best performing enzyme. HMO and DMD candidates were screened in cell lysates fed 3 mM (*S*)-mandelate or phenylglyoxylate respectively. ScHMO (*Streptomyces coelicolor*) and RgDMD (*Rhodotorula graminis*) were selected as the best performing enzymes. (**C**) Screening (*R*)-mandelate pathway constructs in DH5α mutants (*ΔtyrR,ΔtyrA*). After 24 h, plasmid SBC009443 produced 251 mg/L (R)-mandelate with no detectable (S)-mandelate. (**D**) Screening (*R*/*S*)-mandelate pathway constructs in DH5α mutants (*ΔtyrR,ΔtyrA*). After 24 h, plasmid SBC009527 produced (*R*)- and (*S*)-mandelate at 220 and 126 mg/L, respectively (27% e.e.). (**E**) Final (*S*)-mandelate titre screen. SyHMAS was subcloned from pBbE2c (SBC007172) into pBbB5a (SBC010238) and screened in DH5α mutants (*ΔtyrR,ΔtyrA*) with or without the SBC008376 plasmid to boost phenylpyruvate production (Fig. S2). (**F**) Best performing plasmid constructs. PpMR (MR from *Pseudomonas putida*).

Polymandelic acid (PMA) is a thermoplastic with polystyrene-like properties (Liu et al., 2007), which can be thermally degraded for recycling. PMA can be prepared through ring-opening polymerization of mandelide (the cyclic dimer of mandelic acid (MA) (Liu et al., 2007)) or the *O*-carboxyanhydride derivative of MA (Buchard et al., 2014). Chemical synthesis of MA from petroleum-derived feedstocks is environmentally unsustainable, and requires further costly purification of (*R*)- and (*S*)-enantiomers. Therefore, bioproduction of enantiopure MA and derivatives from renewable feedstocks would further improve the green credentials of novel PMA materials. Biosynthetic pathways to MA enantiomers have been established in *E. coli* (Sun et al., 2011). Oxidative decarboxylation of phenylpyruvate (PP) by hydroxymandelic acid synthase (HMAS) generates (*S*)-mandelic acid (SMA), which if desired can be oxidized by hydroxymandelate oxidase (HMO) then reduced by D-mandelate dehydrogenase (DMD) to yield (*R*)-mandelic acid (RMA; Fig. 5A). Engineered *E. coli* strains hosting these pathways produced 740 mg/L of SMA and 680 mg/L of RMA in shake-flask cultures, but PP accumulation suggests the HMAS step is a bottleneck (Sun et al., 2011). For this reason, we focused on looking for alternative enzyme homologs with superior activities for the three biotransformation steps (Fig. 5B, Table S1).

We screened 12 different HMAS genes in our standard gene synthesis plasmid (pBbE2c) in *E. coli* and, although we saw uniformly low SMA titers even for the literature AoHMAS (*Amycolatopsis orientalis*), we identified SyHMAS (*Streptomyces yokosukanensis*) as the best performing homolog. We also conducted *in vitro* screening of three HMO and two DMD enzymes (Fig. 5B, Table S1), but in this case the best performing enzymes ScHMO (*Streptomyces coelicolor*) and RgDMD (*Rhodotorula graminis*) were those from the literature (Sun et al., 2011). The genes for the three best performing enzymes were used to design a library of RMA pathway plasmids, of which we constructed and sequence verified 15 (Table S2). Screening of these plasmids in wildtype DH5α cells revealed a range of RMA titers from 22 to 251 mg/L, with generally no detectable SMA (Fig. 5C). However, it was notable that all plasmids also produced significant quantities of phenylglyoxylate (42–195 mg/L), the intermediate between SMA and RMA, perhaps due to the reversibility or product inhibition of the RgDMD enzyme. As an alternative route to RMA we designed a library of plasmids to express SyHMAS with PpMR (mandelate racemase from *Pseudomonas putida*). We successfully constructed 11 plasmids and screened them in wildtype DH5α (Fig. 5D; Table S2), observing a fairly uniform ratio of 65% RMA to 35% SMA, with the most productive plasmid (SBC009527) yielding 220/126 mg/L RMA/SMA. Whilst this plasmid produced the most MA overall (346 mg/L in total), chiral resolution of these enantiomers would be necessary to yield a useful product, and again the presence of phenylglyoxylate highlights inefficiencies in the pathway.

The pathways to RMA pass first through SMA, suggesting that our SyHMAS enzyme is more productive when expressed from plasmids other than pBbE2c (ColE1 origin, *tet* promoter, *chl*^*R*^). We therefore subcloned SyHMAS into a different plasmid backbone, pBbB5a (BBR1 origin, *lacUV5* promoter, *amp*^*R*^). The original SyHMAS plasmid (SBC007172) was screened alongside the new plasmid (SBC010238) in a mutant DH5α strain (Δ*tyrR,* Δ*tyrA*), with or without co-transformation with a phenylalanine boost plasmid (SBC008376)(Fig. 5E). There was a clear increase in SMA titer when comparing the original SyHMAS plasmid to the new plasmid (33 vs. 141 mg/L), and our highest SMA titer (170 mg/L) was achieved when the new plasmid was expressed in a host with enhanced carbon flux towards the PP substrate.

### 2.9 Optimization of mandelic acid production

**Fig. 6:**
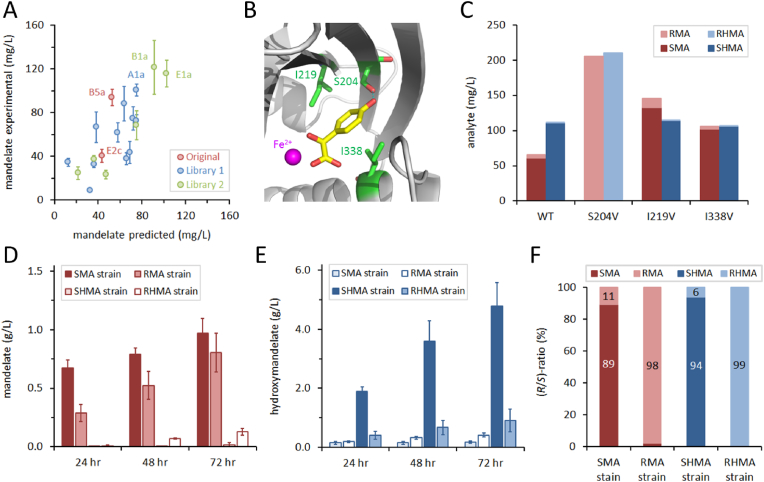
Optimization and scale-up of mandelic acid production. (**A**) Screening SyHMAS performance in different expression vectors. DoE was used to design a library of plasmids with different variables (replication origin, promoter and antibiotic resistance gene). This library (*blue*) was screened in DH5α cells along with two original plasmids (*red*; see Fig. 5). Analysis of mandelic acid titers after 24 h allowed modelling of predicted titers for different vector variables. A second library of 6 plasmids (*green*), predicted to include high, medium and low producers, was then constructed and screened. (**B**) Homology model of SyHMAS, showing the predicted position of hydroxymandelic acid (*yellow sticks*) and the catalytic Fe^2+^ ion. Residues targeted for mutagenesis are labelled in green. (**C**) Production of mandelic acid (*red*) and hydroxymandelic acid (*blue*) by selected SyHMAS mutants. Stacked bar heights represent the combined titer for each analyte, with the relative proportion of (*R*)- and (*S*)- enantiomers indicated (light and dark colors, respectively). (**D**) Mandelic acid titers for bioreactor cultures. Engineered *E. coli* strains hosting optimized mutant SyHMAS expression plasmids were cultured under conditions selected to favor production of the (*R*)- or (*S*)-enantiomers of mandelic acid (RMA & SMA) or hydroxymandelic acid (RHMA & SHMA). (**E**) Hydroxymandelic acid titers for bioreactor cultures described above. (**F**) Chiral analysis of mandelic acid (*red*) and hydroxymandelic acid (*blue*) produced by bioreactor cultures after 72 h. (For interpretation of the references to color in this figure legend, the reader is referred to the Web version of this article.)

From our list of 25 material monomers and precursors we successfully constructed *E. coli* producer strains for 17 targets over an 85-day time limit (Table 1). This showcases the power of the biofoundry approach to rapidly prototype microbial production strains for materials monomer targets. Having developed these strains we anticipate that further significant improvements in target titers could be achieved through a focused optimization of the genetic construct design, further engineering of host strains, and fine-tuning of fermentation conditions for scale-up to bioreactor cultures. To explore this potential, we undertook a further time-limited project (65 days) to optimize and scale-up production of the (*S*)- and (*R*)-enantiomers of MA. We had already observed a significant increase in SMA titer when SyHMAS was subcloned from the pBbE2c vector into pBbB5a (Fig. 5E); this prompted us to investigate other expression vectors from the BglBrick collection (pBb vectors (Lee et al., 2011)). Design of experiments was used to select a library of 12 plasmids, sampling different combinations of replication origins (S = SC101, A = p15A, B = BBR1, E = ColE1), inducible promoters (1 = P*trc*, 2 = P*tet*, 5 = P*lacUV5*, 8 = P*bad*) and antibiotic resistance genes (a = *ampR*, k = *kanR*, c = *camR*). We successfully constructed 11 members of this library and screened them, along with our 2 original plasmids, in wildtype DH5α cultures (Fig. 6A). After 24 h, MA titers for these plasmids ranged from 8 to 100 mg/L, with the highest titer observed for pBbA1a-SyHMAS. This experimental data was used to model the effects of the different vector variables to predict MA titers for other vectors that were not tested. To validate the model, we constructed a second library of 6 plasmids, predicted to encompass high, medium and low MA producers. Screening of this second library showed good correlation between predicted and experimental titers (*R*^2^ = 0.88, *p*-value ≤ 0.006), and identified pBbB1a-SyHMAS (121 mg/L MA) as the optimal plasmid construct. In an attempt to further boost titers, we investigated the effects of gene dosage. We cloned one, two or three copies of SyHMAS (using different redundant codons to minimize sequence homology) into a single expression vector and cultured *E. coli* transformants to measure MA and HMA titers (Figs. S9A and S9B). Whilst we observed no significant effect on MA production, HMA titers increased linearly for each additional copy of SyHMAS present (2.05-fold for two copies, 3.15-fold for three copies after 72 h).

The native substrate for HMAS is hydroxyphenylpyruvate (HPP), which is converted to 4-hydroxymandelic acid (HMA). We anticipated that significant amounts of HMA will be produced as a side-product in our MA cultures, limiting productivity. However, HMA can be considered a material monomer target in its own right, which could be dimerized into hydroxymandelide for ring-opening polymerization. We developed new mass spectrometry methods, first to quantify both mandelic acid and hydroxymandelic acid in our cultures, then to determine relative proportions of (*R*)- and (*S*)-enantiomers for these targets. The specificity of HMAS for HPP over phenylpyruvate (PP) can be influenced by active site mutagenesis, as can the enantioselectivity of product formation (Pratter et al., 2013; Reifenrath and Boles, 2018). We undertook mutagenesis of our SyHMAS enzyme to determine whether we could replace our 3-step RMA pathway with a single enzyme, and to select mutants that favor production of MA over HMA. We prepared a homology model of SyHMAS in SWISS-MODEL (Waterhouse et al., 2018), using the crystal structure of AoHMAS bound to HMA (Brownlee et al., 2008), and selected residues S204, I219 and I338 for mutagenesis (Fig. 6B). Each residue was randomized separately (by PCR using the NNK codon) and the library transformed into *E. coli* DH5α. Automated colony picking selected 96 colonies for each single mutation (3x coverage of NNK codon variants), and MA and HMA titers were quantified in 1-ml cultures after 24 h. Cultures with MA titers higher than wildtype HMAS were then selected for DNA sequencing and chiral analysis of the MA and HMA products (Figs. 6C and S10). Chiral analysis for the wildtype enzyme showed that whilst the HMA produced was >99.9% SHMA, the MA product contained ~7% RMA. The S204V mutation had the greatest effect on MA titers, enhancing these 3.1-fold to 206 mg/L. The S204V mutation also inverted the enantioselectivity of SyHMAS, yielding 92.9% RMA and >99.9% RHMA, an effect that was more pronounced than described for other HMAS homologs (Pratter, 2013; Reifenrath, 2018). In contrast, the I219V mutation enhanced titers 2.2-fold over wildtype whilst retaining the selectivity for SMA & SHMA. The I338V mutation slightly improved the SMA/RMA ratio (96/4%), but reduced overall MA titer compared to I219V (106 mg/L vs. 145 mg/L). We subcloned the S204V and I219V mutants from the pBbB5a vector into both pBbE1a and pBbB1a, the best expression vectors identified from the library screen (Fig. 6A), and confirmed that pBbB1a supported the highest titers of MA and HMA (Figs. S9C and S9D).

In parallel to optimization of our expression constructs, we sought to improve our host strain and identify optimal fermentation conditions for bioreactor scale-up. We already constructed gene knockout strains to support phenylalanine (Δ*tyrR*, Δ*tyrA*) and tyrosine (Δ*tyrR*, Δ*pheLA*) overproduction, along with ancillary plasmids to channel metabolic flux to these targets (SBC008290 and SBC005753 respectively). We further engineered these strains using recombineering with CRISPR editing to integrate the plasmid constructs into the genomes at the *lacZ* locus. This allowed us to use these engineered host strains (identified as DH5α(Phe+) and DH5α (Tyr+)) with just one plasmid (for SyHMAS) and antibiotic. To optimize fermentation conditions (temperature, stirrer speed, O_2_ feed-rate, etc.) we ran pilot fermenter cultures using our original pBbB5a-SyHMAS plasmid in wildtype DH5α cells. We also conducted 1-ml deepwell block cultures to screen different media compositions and carbon sources (Fig. S11A). Once we had constructed our engineered host strains we further screened media requirements for constructs that produced predominantly (*S*)- and (*R*)-enantiomers of MA and HMA (Figs. S11B and S11C). From these screens we identified our optimal strains and media for production of all four targets: SMA strain [DH5α(Phe+) with pBbB1a-SyHMAS(I219V) in SOB-glycerol]; RMA strain [DH5α (Phe+) with pBbB1a-SyHMAS(S204V) in TBP-glycerol]; SHMA strain [DH5α (Tyr+) with pBbB1a-SyHMAS(I219V) in M9-glycerol]; RHMA strain [DH5α (Tyr+) with pBbB1a-SyHMAS(S204V) in M9-glucose]. The four strains were grown in fermenter cultures in triplicate, with media samples taken at 24/48/72 h for quantification of MA (Fig. 6D), HMA (Fig. 6E) and chiral analysis (Fig. 6F). In addition, triplicate bioreactor cultures of the SMA and RMA strains were sampled after 72 h for genome and plasmid resequencing. No single-nucleotide variants were detectable above background noise, indicating that our producer strains were genetically stable over the duration of the fermentation. Our final scaled-up cultures yielded 0.97 ± 0.13 g/L MA (89% *S*-enantiomer), 0.80 ± 0.17 g/L MA (98% *R*-enantiomer), 4.78 ± 0.80 g/L HMA (>94% *S*-enantiomer) and 0.90 ± 0.39 g/L HMA (99% *R*-enantiomer), respectively. Previously, titers of 1.02 g/L SMA and 0.88 g/L RMA were reported for *E. coli* shake-flask cultures (Sun et al., 2011), whereas HMA was produced at 15.8 g/L in fed batch fermentation (Li et al., 2016) but with no indication of enantiopurity.

## 3 Conclusions

We have demonstrated the rapid prototyping capabilities of an automated synthetic biology pipeline for the production of materials monomer targets. We successfully produced 17 chemically diverse key materials building blocks out of 25 selected targets, in some cases with titers close to those reported for optimized *E. coli* fermentations. This was achieved over an 85-day timeframe and without subsequent optimization. Overall, we estimate that the personnel and consumable costs amounted to approximately £15,000 (US$18,500) per successful target compound, corresponding to an average of 360 personnel hours per compound. A second 65-day period was used for optimization and scale-up of strains capable of producing mandelic acid and hydroxymandelic acid. These strains produced 0.8–4.8 g/L of targets with high enantiopurity in fed-batch bioreactor cultures. This rigorous and comprehensive benchmarking study showcases the ability of biofoundries to provide quick access to a diverse range of materials monomers, and demonstrates how prototype microbial production strains can be rapidly scaled-up and optimized to achieve gram-scale fermentations. Through further iterations of the *Design-Build-Test-Learn* cycle, together with subsequent synthetic biology-based chassis engineering approaches for increasing fluxes, regulating enzyme expression and engineering enhanced enzyme performance; microbial production strains can be optimized to meet the technical specifications for industrial biomanufacturing. The rapid prototyping capacity demonstrated here establishes the foundation for a comprehensive biomanufacturing pipeline for the sustainable microbial production of diverse materials monomers.

## Declarations of competing interest

None.

## References

[bib1] Ahmed S.T., Leferink N.G.H., Scrutton N.S. (2019). Chemo-enzymatic routes towards the synthesis of bio-based monomers and polymers. Mol. Catal..

[bib2] Aleku G.A., Prause C., Bradshaw-Allen R.T., Plasch K., Glueck S.M., Bailey S.S., Payne K.A.P., Parker D.A., Faber K., Leys D. (2018). Terminal alkenes from acrylic acid derivatives via non-oxidative enzymatic decarboxylation by ferulic acid decarboxylases. ChemCatChem.

[bib3] Anderson L.A., Islam M.A., Prather K.L.J. (2018). Synthetic biology strategies for improving microbial synthesis of “green” biopolymers. J. Biol. Chem..

[bib4] Appleton E., Madsen C., Roehner N., Densmore D. (2017). Design automation in synthetic biology. Cold Spring Harb. Perspect. Biol..

[bib5] Atsumi S., Hanai T., Liao J.C. (2008). Non-fermentative pathways for synthesis of branched-chain higher alcohols as biofuels. Nature.

[bib6] Bang H.B., Lee K., Lee Y.J., Jeong K.J. (2018). High-level production of trans-cinnamic acid by fed-batch cultivation of Escherichia coli. Process Biochem..

[bib7] Barclay G.G., Hawker C.J., Ito H., Orellana A., Malenfant P.R.L., Sinta R.F. (1998). The “living” free radical synthesis of poly(4-hydroxystyrene): Physical properties and dissolution behavior. Macromolecules.

[bib8] Berner M., Krug D., Bihlmaier C., Vente A., Müller R., Bechthold A. (2006). Genes and enzymes involved in caffeic acid biosynthesis in the actinomycete Saccharothrix espanaensis. J. Bacteriol..

[bib9] Boerjan W., Ralph J., Baucher M. (2003). Lignin biosynthesis. Annu. Rev. Plant Biol..

[bib10] Bouldin R.M., Xia Z., Klement T.J., Kiratitanavit W., Nagarajan R. (2017). Bioinspired flame retardant polymers of tyrosol. J. Appl. Polym. Sci..

[bib11] Bramucci M., Nagarajan V., Thomas S. (2003). Use of Xylene Monooxygenase for the Oxidation of Substituted Monocyclic Aromatic Compounds.

[bib12] Brownlee J., He P., Moran G.R., Harrison D.H.T. (2008). Two roads diverged: the structure of hydroxymandelate synthase from Amycolatopsis orientalis in complex with 4-hydroxymandelate. Biochemistry.

[bib13] Buchard A., Carbery D.R., Davidson M.G., Ivanova P.K., Jeffery B.J., Kociok-Köhn G.I., Lowe J.P. (2014). Preparation of stereoregular isotactic poly(mandelic acid) through organocatalytic ring-opening polymerization of a cyclic O-carboxyanhydride. Angew. Chem. Int. Ed..

[bib14] Carbonell P., Jervis A.J., Robinson C.J., Yan C., Dunstan M., Swainston N., Vinaixa M., Hollywood K.A., Currin A., Rattray N.J.W., Taylor S., Spiess R., Sung R., Williams A.R., Fellows D., Stanford N.J., Mulherin P., Le Feuvre R., Barran P., Goodacre R., Turner N.J., Goble C., Chen G.G., Kell D.B., Micklefield J., Breitling R., Takano E., Faulon J.L., Scrutton N.S. (2018). An automated Design-Build-Test-Learn pipeline for enhanced microbial production of fine chemicals. Commun. Biol..

[bib15] Carbonell P., Wong J., Swainston N., Takano E., Turner N.J., Scrutton N.S., Kell D.B., Breitling R., Faulon J.L. (2018). Selenzyme: enzyme selection tool for pathway design. Bioinformatics.

[bib16] Casini A., Chang F.Y., Eluere R., King A.M., Young E.M., Dudley Q.M., Karim A., Pratt K., Bristol C., Forget A., Ghodasara A., Warden-Rothman R., Gan R., Cristofaro A., Borujeni A.E., Ryu M.H., Li J., Kwon Y.C., Wang H., Tatsis E., Rodriguez-Lopez C., O'Connor S., Medema M.H., Fischbach M.A., Jewett M.C., Voigt C., Gordon D.B. (2018). A pressure test to make 10 molecules in 90 Days: external evaluation of methods to engineer biology. J. Am. Chem. Soc..

[bib17] Cavani F., Trifir F. (1995). Alternative processes for the production of styrene. Appl. Catal. Gen..

[bib18] Chao R., Mishra S., Si T., Zhao H. (2017). Engineering biological systems using automated biofoundries. Metab. Eng..

[bib19] Chen Z., Sun X., Li Y., Yan Y., Yuan Q. (2017). Metabolic engineering of Escherichia coli for microbial synthesis of monolignols. Metab. Eng..

[bib20] Choi K.R., Jang W.D., Yang D., Cho J.S., Park D., Lee S.Y. (2019). Systems metabolic engineering strategies: integrating systems and synthetic biology with metabolic engineering. Trends Biotechnol..

[bib21] Chung D., Kim S.Y., Ahn J.H. (2017). Production of three phenylethanoids, tyrosol, hydroxytyrosol, and salidroside, using plant genes expressing in Escherichia coli. Sci. Rep..

[bib22] Clomburg J.M., Crumbley A.M., Gonzalez R. (2017). Industrial biomanufacturing: the future of chemical production. Science.

[bib23] Currin A., Swainston N., Dunstan M.S., Jervis A.J., Mulherin P., Robinson C.J., Taylor S., Carbonell P., Hollywood K.A., Yan C., Takano E., Scrutton N.S., Breitling R. (2019). Highly multiplexed, fast and accurate nanopore sequencing for verification of synthetic DNA constructs and sequence libraries. Synth. Biol..

[bib24] Davy A.M., Kildegaard H.F., Andersen M.R. (2017). Cell factory engineering. Cell Syst.

[bib25] Delépine B., Duigou T., Carbonell P., Faulon J.L. (2018). RetroPath2.0: a retrosynthesis workflow for metabolic engineers. Metab. Eng..

[bib26] Deng J., Yang B., Chen C., Liang J. (2015). Renewable eugenol-based polymeric oil-absorbent microspheres: preparation and oil absorption ability. ACS Sustain. Chem. Eng..

[bib27] Dumas L., Bonnaud L., Olivier M., Poorteman M., Dubois P. (2016). Chavicol benzoxazine: ultrahigh Tg biobased thermoset with tunable extended network. Eur. Polym. J..

[bib28] Ganewatta M.S., Lokupitiya H.N., Tang C. (2019). Lignin biopolymers in the age of controlled polymerization. Polymers.

[bib29] Hatakeyama H., Hayashi E., Haraguchi T. (1977). Biodegradation of poly (3-methoxy-4-hydroxy styrene). Polymer.

[bib30] Hillson N., Caddick M., Cai Y., Carrasco J.A., Chang M.W., Curach N.C., Bell D.J., Le Feuvre R., Friedman D.C., Fu X., Gold N.D., Herrgård M.J., Holowko M.B., Johnson J.R., Johnson R.A., Keasling J.D., Kitney R.I., Kondo A., Liu C., Martin V.J.J., Menolascina F., Ogino C., Patron N.J., Pavan M., Poh C.L., Pretorius I.S., Rosser S.J., Scrutton N.S., Storch M., Tekotte H., Travnik E., Vickers C.E., Yew W.S., Yuan Y., Zhao H., Freemont P.S. (2019). Building a global alliance of biofoundries. Nat. Commun..

[bib31] Imada M., Takenaka Y., Hatanaka H., Tsuge T., Abe H. (2019). Unique acrylic resins with aromatic side chains by homopolymerization of cinnamic monomers. Commun. Chem..

[bib32] Jansen F., Gillessen B., Mueller F., Commandeur U., Fischer R., Kreuzaler F. (2014). Metabolic engineering for p-coumaryl alcohol production in Escherichia coli by introducing an artificial phenylpropanoid pathway. Biotechnol. Appl. Biochem..

[bib33] Jervis A.J., Carbonell P., Vinaixa M., Dunstan M.S., Hollywood K.A., Robinson C.J., Rattray N.J.W., Yan C., Swainston N., Currin A., Sung R., Toogood H., Taylor S., Faulon J.L., Breitling R., Takano E., Scrutton N.S. (2019). Machine learning of designed translational control allows predictive pathway optimization in Escherichia coli. ACS Synth. Biol..

[bib34] Kang S.Y., Choi O., Lee J.K., Ahn J.O., Ahn J.S., Hwang B.Y., Hong Y.S. (2015). Artificial de novo biosynthesis of hydroxystyrene derivatives in a tyrosine overproducing Escherichia coli strain. Microb. Cell Factories.

[bib35] Kang S.Y., Choi O., Lee J.K., Hwang B.Y., Uhm T.B., Hong Y.S. (2012). Artificial biosynthesis of phenylpropanoic acids in a tyrosine overproducing Escherichia coli strain. Microb. Cell Factories.

[bib36] Kaufman T.S. (2015). The multiple faces of Eugenol. A versatile starting material and building block for organic and bio-organic synthesis and a convenient precursor toward bio-based fine chemicals. J. Braz. Chem. Soc..

[bib37] Kawaguchi H., Ogino C., Kondo A. (2017). Microbial conversion of biomass into bio-based polymers. Bioresour. Technol..

[bib38] Kikuchi Y., Tsujimoto K., Kurahashi O. (1997). Mutational analysis of the feedback sites of phenylalanine-sensitive synthase of Escherichia coli.

[bib39] Kim B., Binkley R., Kim H.U., Lee S.Y. (2018). Metabolic engineering of Escherichia coli for the enhanced production of l-tyrosine. Biotechnol. Bioeng..

[bib40] Kim S.J., Vassão D.G., Moinuddin S.G.A., Bedgar D.L., Davin L.B., Lewis N.G. (2014). Allyl/propenyl phenol synthases from the creosote bush and engineering production of specialty/commodity chemicals, eugenol/isoeugenol, in Escherichia coli. Arch. Biochem. Biophys..

[bib41] Le Feuvre R.A., Scrutton N.S. (2018). A living foundry for Synthetic Biological Materials: a synthetic biology roadmap to new advanced materials. Synth. Syst. Biotechnol..

[bib42] Lee T.S., Krupa R.A., Zhang F., Hajimorad M., Holtz W.J., Prasad N., Lee S.K., Keasling J.D. (2011). BglBrick vectors and datasheets: a synthetic biology platform for gene expression. J. Biol. Eng..

[bib43] Li F.F., Zhao Y., Li B.Z., Qiao J.J., Zhao G.R. (2016). Engineering Escherichia coli for production of 4-hydroxymandelic acid using glucose-xylose mixture. Microb. Cell Factories.

[bib44] Liu T., Simmons T.L., Bohnsack D.A., Mackay M.E., Smith M.R., Baker G.L. (2007). Synthesis of polymandelide: a degradable polylactide derivative with polystyrene-like properties. Macromolecules.

[bib45] Liu Y., Xu Y., Ding D., Wen J., Zhu B., Zhang D. (2018). Genetic engineering of Escherichia coli to improve L-phenylalanine production. BMC Biotechnol..

[bib46] Luo Z.W., Lee S.Y. (2017). Biotransformation of p-xylene into terephthalic acid by engineered Escherichia coli. Nat. Commun..

[bib47] Lütke-Eversloh T., Stephanopoulos G. (2005). Feedback inhibition of chorismate mutase/prephenate dehydrogenase (TyrA) of Escherichia coli: generation and characterization of tyrosine-insensitive mutants. Appl. Environ. Microbiol..

[bib48] Machas M., Kurgan G., Jha A.K., Flores A., Schneider A., Coyle S., Varman A.M., Wang X., Nielsen D.R. (2019). Emerging tools, enabling technologies, and future opportunities for the bioproduction of aromatic chemicals. J. Chem. Technol. Biotechnol..

[bib49] Marliere P. (2011). Method for Producing an Alkene Comprising the Step of Converting an Alcohol by an Enzymatic Dehydration Step.

[bib50] McKenna R., Moya L., McDaniel M., Nielsen D.R. (2015). Comparing in situ removal strategies for improving styrene bioproduction. Bioproc. Biosyst. Eng..

[bib51] Nelms J., Edwards R.M., Warwick J., Fotheringham I. (1992). Novel mutations in the pheA gene of Escherichia coli K-12 which result in highly feedback inhibition-resistant variants of chorismate mutase/prephenate dehydratase. Appl. Environ. Microbiol..

[bib52] Önnerud H., Zhang L., Gellerstedt G., Henriksson G. (2002). Polymerization of monolignols by redox shuttle-mediated enzymatic oxidation: a new model in lignin biosynthesis I. Plant Cell.

[bib53] Pratter S.M., Konstantinovics C., Di Giuro C.M.L., Leitner E., Kumar D., De Visser S.P., Grogan G., Straganz G.D. (2013). Inversion of enantioselectivity of a mononuclear non-heme iron(II)-dependent hydroxylase by tuning the interplay of metal-center geometry and protein structure. Angew. Chem. Int. Ed..

[bib54] Quintens G., Vrijsen J.H., Adriaensens P., Vanderzande D., Junkers T. (2019). Muconic acid esters as bio-based acrylate mimics. Polym. Chem..

[bib55] Reifenrath M., Boles E. (2018). Engineering of hydroxymandelate synthases and the aromatic amino acid pathway enables de novo biosynthesis of mandelic and 4-hydroxymandelic acid with Saccharomyces cerevisiae. Metab. Eng..

[bib56] Rivas B.L., Maturana H.A., Jesús Molina M., Gómez-Antón M.R., Piérola I.F. (1998). Metal ion binding properties of poly(N-vinylimidazole) hydrogels. J. Appl. Polym. Sci..

[bib57] Rodriguez A., Martínez J.A., Flores N., Escalante A., Gosset G., Bolivar F. (2014). Engineering Escherichia coli to overproduce aromatic amino acids and derived compounds. Microb. Cell Factories.

[bib58] Rojo L., Vazquez B., Parra J., Bravo A.L., Deb S., San Roman J. (2006). From natural products to polymeric derivatives of “Eugenol”: a new approach for preparation of dental composites and orthopedic bone cements. Biomacromolecules.

[bib59] Satoh Y., Tajima K., Munekata M., Keasling J.D., Lee T.S. (2012). Engineering of a tyrosol-producing pathway, utilizing simple sugar and the central metabolic tyrosine, in Escherichia coli. J. Agric. Food Chem..

[bib60] Smanski M.J., Zhou H., Claesen J., Shen B., Fischbach M.A., Voigt C.A. (2016). Synthetic biology to access and expand nature's chemical diversity. Nat. Rev. Microbiol..

[bib61] Sun Z., Ning Y., Liu L., Liu Y., Sun B., Jiang W., Yang C., Yang S. (2011). Metabolic engineering of the L-phenylalanine pathway in Escherichia coli for the production of S- or R-mandelic acid. Microb. Cell Factories.

[bib62] Takeshima H., Satoh K., Kamigaito M. (2017). Bio-based functional styrene monomers derived from naturally occurring ferulic acid for poly(vinylcatechol) and poly(vinylguaiacol) via controlled radical polymerization. Macromolecules.

[bib63] Terao Y., Satoh K., Kamigaito M. (2019). Controlled radical copolymerization of cinnamic derivatives as renewable vinyl monomers with both acrylic and styrenic substituents: reactivity, regioselectivity, properties, and functions. Biomacromolecules.

[bib64] Thi T.H., Matsusaki M., Shi D., Kaneko T., Akashi M. (2008). Synthesis and properties of coumaric acid derivative homo-polymers. J. Biomater. Sci. Polym. Ed..

[bib65] Thompson B., Pugh S., Machas M., Nielsen D.R. (2018). Muconic acid production via alternative pathways and a synthetic “metabolic funnel. ACS Synth. Biol..

[bib66] Wagner H.J., Engesser R., Ermes K., Geraths C., Timmer J., Weber W. (2019). Synthetic biology-inspired design of signal-amplifying materials systems. Mater. Today.

[bib67] Waterhouse A., Bertoni M., Bienert S., Studer G., Tauriello G., Gumienny R., Heer F.T., De Beer T.A.P., Rempfer C., Bordoli L., Lepore R., Schwede T. (2018). SWISS-MODEL: homology modelling of protein structures and complexes. Nucleic Acids Res..

[bib68] Weaver L.M., Herrmann K.M. (1990). Cloning of an aroF allele encoding a tyrosine-insensitive 3-deoxy-D-arabino-heptuloxsonate 7-phosphate synthase. J. Bacteriol..

[bib69] Wehrs M., Tanjore D., Eng T., Lievense J., Pray T.R., Mukhopadhyay A. (2019). Engineering robust production microbes for large-scale cultivation. Trends Microbiol..

[bib70] Xiong M., Yu P., Wang J., Zhang K. (2015). Improving engineered Escherichia coli strains for high-level biosynthesis of isobutyrate. AIMS Bioeng.

[bib71] Yang C., Chen X., Chang J., Zhang L., Xu W., Shen W., Fan Y. (2018). Reconstruction of tyrosol synthetic pathways in Escherichia coli. Chin. J. Chem. Eng..

[bib72] Zhang R., Zhao C.H., Chang H.C., Chai M.Z., Li B.Z., Yuan Y.J. (2019). Lignin valorization meets synthetic biology. Eng. Life Sci..

[bib73] Zhu Y., Romain C., Williams C.K. (2016). Sustainable polymers from renewable resources. Nature.

