# Supplementary material for: Rapid prototyping of microbial production strains for the biomanufacture of potential materials monomers
Source: Metab Eng. 2020 Jul;60:168–82. doi: 10.1016/j.ymben.2020.04.008 (PMC7225752; doi:10.1016/j.ymben.2020.04.008)
Supplement: Multimedia component 1 [file mmc1.docx]

**Supplementary Information**

**Rapid prototyping of microbial production strains for the biomanufacture of potential materials monomers**

Christopher J. Robinson^1^, Pablo Carbonell^1^, Adrian J. Jervis^1^, Cunyu Yan^1^, Katherine A. Hollywood^1^, Mark S. Dunstan^1^, Andrew Currin^1^, Neil Swainston^1^, Reynard Spiess^1^, Sandra Taylor^1^, Paul Mulherin^1^, Steven Parker^1^, Nicholas E. Matthews^1,2^, William Rowe^1^, Kirk J. Malone^1^, Rosalind Le Feuvre^1^, Philip Shapira^,1,2^, Perdita Barran^1,3^, Nicholas J. Turner^1,3^, Jason Micklefield^1,3^, Rainer Breitling^1,3^, Eriko Takano^1,3^, Nigel S. Scrutton^1,3^.

^1^ Manchester Centre for Synthetic Biology of Fine and Speciality Chemicals (SYNBIOCHEM), Manchester Institute of Biotechnology, The University of Manchester, Manchester M1 7DN, UK.

^2^ Manchester Institute of Innovation Research, Alliance Manchester Business School, The University of Manchester, Manchester M15 6PB, UK.

^3^ School of Chemistry, The University of Manchester, Manchester M13 9PL, UK.

**Table of Contents**

Methods

**1. Design**

*1.1.Target selection*4

*1.2. Enzyme and pathway selection*4

*1.3. DNA part design*4

*1.4. Pathway design*5

*1.5. DNA synthesis*6

**2. Build**

*2.1. Bacterial strains and media*6

*2.2. Genome engineering*6

*2.3. Pathway assembly*7

*2.4. Mutagenesis*7

*2.5. Next-generation sequencing*8

**3. Test**

*3.1. In vitro enzyme screening*9

*3.2. In vivo enzyme and pathway screening*9

*3.3. Bioreactor scale-up*10

*3.4. Quantification of target compounds*10

*3.4.1. LC-TripleQuad : LC-MS/MS analysis*11

*3.4.2. LC-IMS QToF: GC-MS analysis*12

*3.4.3. GC-QToF: GC-MS sample preparation and analysis*13

**4. Learn**

*4.1. Plasmid vector optimization*15

**References**16-17

**Supplementary tables**

*Table S1. Summary of enzyme candidates and screening results..*18-20

*Table S2. Summary of plasmid constructs and screening results..*21-22

*Table S3. Chemical standards and substrates.*23

*Table S4. E. coli strains used in this study..*24

*Table S5. General DNA parts for construction of plasmid constructs..*25

*Table S6. DNA primers used for gene knockout and plasmid construction...*26

**Supplementary figures**

*Figure S1. The DBTL cycle for microbial production of materials monomers*27

*Figure S2. Metabolic engineering of E. coli cells to increase flux down the shikimate pathway*28

*Figure S3. Design, Build & Test of biosynthetic pathways towards ferulic acid*29

*Figure S4. Design, Build & Test of biosynthetic pathways towards coniferol and eugenol*30

*Figure S5. Design, Build & Test of biosynthetic pathways towards muconic acid and diene targets*31

*Figure S6. Design & Test of candidate enzymes for biosynthesis of tyrosol*32

*Figure S7. Design & Test of candidate enzymes for biosynthesis of benzene dicarboxylate targets*33

*Figure S8. Design, Build & Test of biosynthetic pathways towards isobutyl compound targets*34

*Figure S9. Optimization of plasmid constructs for production of mandelic/hydroxymandelic acid*35

*Figure S10. Mutagenesis of hydroxymandelic acid synthase*36

*Figure S11. Optimization of E.coli growth media for production of mandelic/hydroxymandelic acid*37

**Supplementary - Design of Experiments** (separate file)

*Table D1. PCR primers for LCR part preparation.*

*Table D2. Bridging oligonucleotides for LCR assembly of plasmids.*

*Figure D1-D18. Combinatorial design of experiments for DE13, DE22, DE27, DE28, DE31, DE32, DE33, DE35, DE36, DE37, DE38, DE39, DE40, DE42, DE45, DE46, DE47, and DE49.*

# Methods

## Design

### Target selection

Initial research consisted of discussions with colleagues at the School of Materials at the University of Manchester and with contacts in the materials industry, in order to identify platform chemicals that could be amenable targets for biosynthesis in *E. coli*. We discounted targets which were endogenous metabolites (e.g. lactic acid, glucaric acid), targets for which bio-based production had been extensively optimized (e.g. itaconic acid, 1,3-butanediol) and targets that did not seem accessible through conventional biosynthetic routes (e.g. levoglucosenone, 5-hydroxymethyl-furfural). Further discussion and feasibility studies enabled us to create a shortlist of 22 targets of interest which were either primary targets, alternative targets accessible through the same biosynthetic pathways, or key intermediate compounds with material uses (Table 1). In addition, we formulated host cell engineering strategies to boost the levels of 3 primary metabolites (tyrosine, phenylalanine and 2-oxoisovalerate) that serve as common substrates for several of our planned biosynthetic pathways (Figs. S2 and S8).

### Enzyme and pathway selection

Targets were submitted to RetroPath (Delépine et al., 2018) for bio-retrosynthetic analysis. Reaction rules from RetroRules (Duigou et al., 2019) were run at different diameters and pathway parameters. For those targets where no pathway was found, synthetic routes were explored using the Reaxys Synthesis Planner (Lawson et al., 2014). Precursors involved in synthetic chemistry routes were also submitted to RetroPath in order to identify semi-synthetic pathways. For each reaction step in the pathway, candidate enzyme sequences were selected (typically 5 orthologs for each enzyme). The selection was assisted by Selenzyme (Carbonell et al., 2018b), with ranked candidates assessed manually to favor enzymes with literature-validated activity and to ensure candidates were selected from a broad phylogenetic range of source organisms. In total, 128 enzymes were selected from 88 different species and encoded enzymes for the catalysis of 50 distinct target reactions (Table S1).

### DNA part design

All enzyme-encoding genes were designed using PartsGenie (Swainston et al., 2018), optimizing the coding sequence for expression in *E. coli* and including a ribosome binding site optimized for a translation initiation rate of 20,000 using the RBS calculator (Espah Borujeni et al., 2013). In addition, a unique randomized 5′-sequence with a Tm of 70 °C was added to facilitate pathway assembly (see below). All DNA parts were automatically stored in a local installation of JBEI-ICE (Ham et al., 2012), where they were assigned part ID numbers (SBC numbers). SBC numbers for the gene parts used in this study are listed alongside the corresponding enzymes in Table S1, whilst SBC numbers for plasmid backbone parts are listed in Table S5. In total, the project required the synthesis of 111 new gene parts (~155 kb of DNA), along with 25 gene parts and 18 plasmid backbone parts already in house (a further ~57 kb of DNA).

### Pathway design

For convenience of assembly/screening or sharing common genes between pathways, the genes for some pathways were split into modules carried on different compatible plasmids (Fig. S1B). These pathways/modules were given design (DE) numbers, as listed below. Figures for the relevant biosynthetic pathways are also listed.

**DE13** tyrosine overproduction genes (4 genes: Fig S2)

**DE22** tyrosine to tyrosol pathway (4 genes: Fig S6)

**DE27** 2-oxoisovalerate overproduction genes (4 genes: Fig S8)

**DE28** 2-oxoisovalerate to isobutene pathway (1-3 genes: Fig S8)

**DE29** 2-oxoisovalerate to isobutyric acid pathway (2-4 genes: Fig S8)

**DE30** xylene to benzene dicarboxylates pathway (4 genes: Fig S7)

**DE31** phenylpyruvate to (*S*)-mandelate (1 gene: Fig 5)

**DE32** phenylpyruvate to (*R*)-mandelate pathway (3 genes: Fig 5)

**DE33** 3-dehydroshikimate/chorismate to *cis,cis-*muconate pathway (5 genes: Fig S5)

**DE35** phenylalanine overproduction genes (4 genes: Fig S2)

**DE36** phenylalanine to cinnamic acid (1 gene: Fig 3)

**DE37** histidine to urocanic acid (1 gene: Fig 3)

**DE38** tyrosine to coumaric acid (1 gene: Figs 3)

**DE39** tyrosine to ferulic acid pathway (3 genes: Figs 3 & S3)

**DE40** phenylacrylic acids to monolignols (3 genes: Figs 4 & S4)

**DE42** monolignols to allylbenzenes pathway (2 genes: Figs 4 & S4)

**DE45** decarboxylation of muconic acid & acrylic acid compounds (2 genes: Figs 3 & S5)

**DE46** phenylpyruvate to (*S/R*)-mandelate pathway (2 genes: Fig 5)

**DE49** phenylpyruvate to (*S*)-mandelate, subclone (1 gene: Fig 5)

**DE54** phenylpyruvate to (*S*)-mandelate, gene dosage library (1-3 genes)

**DE55** phenylpyruvate to (*S*)-mandelate, subclone library 1 (1 gene)

**DE56** phenylpyruvate to (*S*)-mandelate, subclone library 2 (1 gene)

**DE58** phenylpyruvate to (*S*)- or (*R*)-mandelate, subcloned mutants (1 gene)

For each design, where viable enzyme candidates were identified, multiple variant plasmids were designed using a *D*-optimal *Design of experiments* (DoE) approach (Goos & Jones, 2011). Varied factors included plasmid copy number (ColE1, p15a, BBR1 or SC101 origins of replication), promoter strength (Ptrc or PlacUV5 promoters), candidate gene selection, and sequential ordering of genes/promoters within the plasmid. Library sizes were selected in the range of 2-48 plasmids, depending on the number of factors and combinatorial space. Full DoE libraries, illustrating each plasmid pathway design, can be found in the ‘*Design of Experiments*’ dataset, accessible from the Mendeley Data repository (<http://dx.doi.org/10.17632/t4jtcf9dr2.2>).

Following DoE, Synthetic DNA designs were automatically assigned SBC numbers and stored in a local installation of JBEI-ICE (Ham et al., 2012), through its integration with the design tools PartsGenie (Swainston et al., 2018) and PlasmidGenie (Robinson et al., 2018). Lists of 5′-phosphorylated PCR primer pairs were automatically generated for the preparation of DNA parts. Bridging oligonucleotides to enable the assembly of DNA parts into biosynthetic pathway plasmids were designed using PlasmidGenie, which was more closely integrated with the DoE design pipeline through the development of a Python-based client interface (<https://github.com/synbiochem/PlasmidGenieClient>). PCR primers (Table D1) and bridging oligonucleotides (Table D2) for LCR assembly of plasmid libraries (Figures D1-D18) are included in the ‘*Design of Experiments*’ dataset, accessible from the Mendeley Data repository (<http://dx.doi.org/10.17632/t4jtcf9dr2.2>).

### DNA synthesis

All synthetic DNA was ordered from Twist Biosciences (San Francisco, USA) or GeneArt / Thermo Fisher (Regensburg, Germany). Synthetic gene parts were custom cloned into expression vectors (pBbE2c or pBbA1k) to allow for direct expression in *E. coli* cells. PCR primers and bridging oligomers were synthesized by Integrated DNA Technologies (Leuven, Belgium).

## Build

### Bacterial strains and media

*Escherichia coli* DH5α or DH10β strains (New England Biolabs) were used for routine cloning and plasmid propagation. Strains were maintained on Lysogeny Broth (LB, Formedium Ltd.) or LB-agar containing appropriate antibiotics for plasmid selection. Cells transformed with plasmid assembly reactions were plated on LB-agar supplemented with 1% glucose and appropriate antibiotics. Standard antibiotic concentrations used were: 100 µg/mL ampicillin; 50 µg/mL kanamycin; 20 µg/mL chloramphenicol; 100 µg/mL streptomycin; or 150 μg /mL hygromycin B. All strains used in this study are listed in Table S4.

### Genome engineering

*E. coli* gene knockouts were accomplished by standard lambda Red recombineering (Datsenko & Wanner, 2000). Briefly, the kanamycin resistance cassette-disrupted genes from the corresponding KEIO strains (Baba et al., 2006) were PCR amplified from genomic DNA preparations using primers designed to include ~250 bp sequences which flank the target gene in the *E. coli* genome (Table S6). Cells were transformed with the pSIM18 plasmid (Datta et al., 2006), grown to an OD600 of 0.3, then heat shocked at 42 °C for 15 min to induce FLP recombinase expression; electrocompetent cells were then prepared from these cultures. 50 µl of electrocompetent cells were transformed with 300 ng of the kanamycin cassette PCR product, then plated on agar plates containing 50 µg /mL kanamycin. Correct knockout of genes was confirmed by colony PCR using primers designed to flank the recombination sites (Table S6). Cells were cured of the pSIM18 plasmid by growth in liquid culture at 43 °C for 4-5 hr, with loss confirmed by replica plating single colonies on LB-agar plates containing 50 μg /mL kanamycin (growth) or 150 μg /mL hygromycin B (no growth). Kanamycin resistance cassettes were subsequently removed by transformation of cells with the pCP20 plasmid (Cherepanov & Wackernagel, 1995), followed by growth at 43 °C for 4-5 hr in LB media with no antibiotics. Cultures were plated on LB-agar with no antibiotics, then replica plated onto LB-agar plates containing 50 μg /mL kanamycin (no growth) or 100 μg /mL ampicillin (no growth) to confirm removal of the Kanamycin resistance cassette and loss of pCP20, respectively.

Pathways were integrated into the *E. coli* genome at the *lacZ* locus using lambda Red recombineering facilitated by CRISPR editing. pSIM18 was modified to encode Cpf1 from *Acidaminococcus sp.*, constitutively expressed from promoter JS23151 (pSIMcpf1), and an arabinose-inducible CRISPR-RNA (crRNA) designed to target the pMB1 origin of pTFlacZ. Donor pathway DNA, including an associated *lacI* gene, were cloned into plasmid pTFlacZ between two 500 bp *lacZ* gene fragments. pTFlacZ also encodes a constitutively expressed crRNA designed to cleave the genomic *lacZ* gene to generate corresponding 500 bp *lacZ* gene fragments. Target *E. coli* strains containing plasmid pSIMcpf1 were prepared as electrocompetent cells and transformed with pTFlacZ plasmids (pTFlacZ-8376 or pTFlacZ-5753), then recovered in LB for 2 hr at 30 °C prior to plating on LB-agar containing streptomycin and hygromycin. Isolated colonies were inoculated into LB supplemented with hygromycin and 0.2 % w/v arabinose and grown at 30 °C overnight to cure of pTFlacZ plasmids. Overnight cultures were diluted 1:1000 into LB and further grown at 37 °C to cure of pSIMcpf1, before serial dilutions were plated onto LB-agar. Single colonies were replica plated onto LB-agar with no antibiotics, with streptomycin, or with hygromycin, and clones showing loss of both plasmids were PCR screened for correct integration.

### Pathway assembly

Plasmid-borne pathways were assembled using our previously described semi-automated pipeline (Carbonell et al., 2018a; Robinson et al., 2018). The assembly pipeline follows a number of steps, including PCR-based amplification of parts, part processing and electrophoretic analysis, followed by robot-driven ligase cycling reaction (LCR). Worklists for driving liquid handling robots were developed through custom software (<https://github.com/synbiochem/AssemblyGenie>). The supplementary file – ‘*Design of Experiments*’ lists PCR primers (Table D1) and bridging oligonucleotides (Table D2) for LCR assembly of plasmid libraries (Figures D1-D18). LCR reactions were transformed into *E. coli* DH5α and grown on LB-agar supplemented with 1% glucose and selective antibiotics. Plasmid DNA was prepared from 4-6 clones for each assembly for DNA sequencing, and one clone for each sequence-verified pathway was archived and tested.

In some cases, plasmids were prepared manually by In-Fusion cloning (Takara Bio Inc). PCR primers were designed with 15 nt overhangs to anneal DNA fragments through sequence complementarity. This was done to create plasmids: SBC007575, SBC007580, SBC007583, SBC007589 (subcloning genes from pG9m2-AtPAL and pG9m2-FjTAL into pBbA1a and pBbA5a plasmids); SBC007639 (replacing T7 promoters in pCDFduet-AnFDC-PaUbiX with *trc* promoters); and SBC010238 (removing the ScHMO-PlacUV5-RgDMD fragment from plasmid SBC009478). In-Fusion was also used to subclone SyHMAS into different plasmid vectors (SBC010706-10826) and to construct ‘gene dosage’ plasmids with 2 and 3 copies of SyHMAS (SBC010779-10780). For the gene dosage constructs, SyHMAS genes with identical amino acid sequences but divergent codon usage were designed and synthesised with upstream terminator and promoter sequences (TB1006-Ptrc). PCR primers and sequencing primers for these plasmid preparations are listed in Table S6. The pCDFduet-AnFDC-PaUbiX plasmid was kindly provided by Professor David Leys (University of Manchester).

All plasmids that were constructed, sequence-verified and tested in this study are listed in Table S2.

### Mutagenesis

Variant libraries of SyHMAS mutants were generated using an asymmetric PCR method for mutagenesis (as described in: Sadler et al., 2018; Currin et al., 2019a). Each selected amino acid was randomised using the NNK degenerate codon, encoded in the mutagenic PCR primers (Table S6). Gene variants were cloned into the pBbE1a expression vector by In-Fusion cloning, then *E. coli* DH5α cells were transformed and spread onto LB-agar plates containing 100 µg/ml ampicillin. Individual colonies were selected (QPix 400 colony picker, Molecular Devices LLC) and inoculated into 1 mL of phosphate-buffered Terrific Broth (TBP; Formedium Ltd) in 96-deepwell plates (DWPs) for *in vivo* enzyme screening. Cell pellets were prepared for each DWP culture and stored (-80 °C). Plasmid DNA samples were prepared using an automated miniprep protocol (QIAcube, Qiagen NV) for Sanger sequencing (Eurofins Scientific) to identify variant mutations with improved activity.

### Next-generation sequencing

Successful assembly of plasmids were validated through a novel next-generation sequencing pipeline, based on MinION next-generation sequencing data (Oxford Nanopore Technologies). Individual plasmids were uniquely barcoded, and the sequencing pipeline includes de-multiplexing, sequence alignment and variant analysis (Currin et al., 2019b). The software analysis pipeline is based on Samtools and BCFtools (<http://www.htslib.org>), and has been optimized to run on cloud computing platforms. It is publicly available at <https://github.com/synbiochem/SequenceGenie>.

Samples from 6 bioreactor cultures (mandelic acid production strains), and one control culture without plasmid, were recovered after 72 hr and gDNA was extracted (Monarch Genomic DNA Purification Kit, NEB). DNA was sheared (g-TUBE, Covaris) to produce 10kb fragments, barcoded and prepared for sequencing using the SMRTbell Express Template Prep Kit 2.0 (Pacific Biosciences). All 7 samples were multiplexed on a single SMRT cell then sequenced on a Sequel II system (Pacific Biosciences). Whole genome and plasmid DNA sequence analysis was performed using Geneious Prime 2020.1 software package. Sequencing from the control culture was *de novo* assembled into a single circular contig using the default microbial assembly algorithm, bioreactor samples were then re-sequenced using the control genome and plasmid maps as reference sequences.

## Test

### In vitro enzyme screening

Enzyme screening was carried out using clarified lysates from *E. coli* cultures expressing individual recombinant enzymes. Synthetic genes were custom cloned into pBbE2c or pBbA1k expression vectors for this purpose, and archived at -80 ° as glycerol stocks of DH10β transformants. Glycerol stocks were used to inoculate 1 mL aliquots of phosphate-buffered Terrific Broth (TBP; Formedium, UK) with appropriate antibiotics in 96-deepwell plates (DWP). DWPs were sealed with breathable plate seals and incubated overnight at 30 °C with shaking at 950 rpm. Overnight cultures were diluted 1/50 into fresh media, and returned to the shaker-incubator until an OD_600_ (optical density at 600 nm) of ~0.6 was reached, whereupon cultures were induced by adding isopropyl ß-D-1-thiogalactopyranoside (IPTG) or anhydrotetracycline (aTet) to final concentrations of 100 µM or 200 nM, respectively. Cultures were then returned to the shaker-incubator for 24 hr, before centrifugation (2700 x g, 10 min) and removal of culture media. Cell pellets were resuspended in 800 µl of phosphate-buffered saline (PBS), supplemented with 10% glycerol, 5 mM MgCl_2_, 1 mM dithiothreitol, 50 µg/mL DNaseI and protease inhibitor cocktail (Roche), then transferred to a 96-tube array of 2 mL FastPrep tubes containing Lysing Matrix B (MPBio). FastPrep tubes were loaded into a FastPrep-96 homogenizer and pulsed for 3 x 20 sec at 1600 rpm to lyse cells. Lysates were clarified by centrifugation (2700 x g, 10 min), aliquoted into 96-well assay plates and frozen at -80 °C.

Enzyme assays were conducted in 96-well PCR plates (Bio-Rad) in a final volume of 20 µl. 19 µl of lysate (or 2 x 9.5 µl aliquots of two lysates for combined enzyme assays) and 1 µl of 60 mM substrate were mixed for each assay, using pipetting worklists on a Hamilton Star robotic platform. Where necessary, 1 µl of 60 mM enzyme cofactors were also added to reactions (NAD+, NADPH, SAM). Plates were sealed and incubated in a shaker-incubator at 30 °C, 950 rpm. 1 µl samples were removed at 4 hr and 24 hr, then mixed with 10% methanol at a dilution factor between 50x and 1000x depending on the downstream MS analysis method. Some reactions involved volatile substrates or products, in which case these assays were prepared manually and incubated inside sealed 200 µl glass HPLC vials. Results from *in vitro* enzyme screens are presented as mean values ± standard deviation, for duplicate samples prepared from separately prepared *E. coli* cultures.

### In vivo enzyme and pathway screening

Pathways were screened *in vivo* in a 96-array format using one robot-driven workflow, with a few exceptions (detailed below) where the nature of the substrates or products required modifications. *E. coli* cells (typically DH5α, unless otherwise specified) were heat-shock transformed with plasmid DNA and spread onto LB-agar plates containing selective antibiotics. Four colonies from each transformation were picked into 1 mL aliquots of TBP (supplemented with 0.4% glycerol and antibiotics) in a DWP. DWPs were sealed with breathable plate seals and incubated overnight at 30 °C with shaking at 950 rpm. Overnight cultures were diluted 1/50 into fresh media, and returned to the shaker-incubator until an OD_600_ of 1.0 – 2.0 was reached, whereupon cultures were induced by adding IPTG to a final concentration of 100 µM. Where appropriate, pathway substrate compounds were also added at this stage to a final concentration of 3 mM. Cultures were then returned to the shaker-incubator for 24 hr. Following incubation, the OD_600_ was recorded and 100 µl of culture was quenched with 100 µl of methanol. Samples were thoroughly mixed by vortexing, and clarified by centrifugation (2700 x g, 10 min), prior to dilution with 10% methanol or H_2_O for MS analysis (typically samples were diluted 50x to 16000x, depending on the MS analysis method and expected analyte concentration). Results from *in vivo* pathway screens are presented as mean values ± standard deviation, for quadruplicate cultures picked as separate colonies from freshly-transformed cells.

For cell media screening, colonies were picked into 1 mL aliquots of various media formulations (supplemented with antibiotics and 0.4% glycerol or glucose) in a DWP. DWPs were sealed with breathable plate seals and incubated overnight at 30 °C with shaking at 950 rpm. Overnight cultures were diluted 1/50 into fresh media, and screening proceeded as described above. Media used were: TBP, Super Optimal Broth (SOB) and auto-induction TB media (AIM) from Formedium Ltd; EZ rich defined MOPS medium from Teknova Inc.; and M9 media (Sambrook & Russell 2001) prepared in house with (1x) M9 salts, 2 mM MgSO_4_, 0.1 mM CaCl_2_ 0.2 % w/v Casamino acids, 100 mg/L thiamine and (1x) trace element solution. All media were supplemented with antibiotics and 0.4% glycerol or glucose, as appropriate.

For pathways with volatile products, a modified workflow was required. Following induction by IPTG, 1 mL cultures were transferred to 20 mL headspace vials and overlaid with 0.5 mL of 2,2,4-trimethylpentane (TMP). Vials were sealed with gas-tight screw caps, then secured in the shaker-incubator overnight at 37 °C, 200 rpm. After 24 hr incubation, the OD_600_ of the cultures were recorded before the cultures and overlays were transferred to 2 mL Eppendorf tubes and centrifuged (10,000 x g, 3 min) to pellet cells. The clarified cell media and TMP overlay were recovered separately and prepared for GC-MS analysis as described below. Results from volatile product screens are presented as mean values ± standard deviation, for duplicate cultures picked as separate colonies from freshly-transformed cells.

### Bioreactor scale-up

Fermentation scale-up was conducted on a Multifors 2 bioreactor assembly (Infors HT) equipped with 6 x 1 L vessels, with a working culture volume of 650 mL per vessel. The pH and dissolved oxygen (dO_2_) probes were calibrated according to the manufacturer’s guidelines at the start of each fermentation cycle. Culture conditions were optimized through a series of preliminary experiments (data not shown), with subsequent cultures maintained at a constant pH 7.0 at 37 °C. The stirrer speed was linked to dO_2_ concentration to maintain a maximum dO2 (> 90%) within each vessel, with stirrer speeds ranging over 300-1200 rpm. Foaming of the culture media was controlled by the automatic addition of antifoam solution. The pH was controlled by the automatic addition of 2M phosphoric acid or 2M sodium hydroxide solutions.

Culture media were autoclaved in the fermenter vessels, with syringe-filtered supplements and antibiotics added immediately before inoculation. Starter cultures were grown in corresponding media at 37 °C in a shaker-incubator overnight, then added aseptically to the culture vessels at a dilution ratio of 1 in 100. Cultures were induced at an OD600 of ~2.0 by adding IPTG to a final concentration of 100 µM. Cultures were supplemented with glycerol at 12 hr intervals (0.4% w/v) resulting in the total addition of 2.4% w/v glycerol over the 72 hr fermentation period. Culture samples were collected at 72 hr for next-generation sequencing to assess the stability of genomic and plasmid DNA. Samples (1 mL) were also collected aseptically at 24, 48 and 72 hr time-points, then centrifuged (10,000 × g, 3 min) to pellet cells, with clarified cell media diluted (1:16000) in water for LC-MS/MS analysis. Results from bioreactor cultures are presented as mean values ± standard deviation, for triplicate cultures picked as separate colonies from freshly-transformed cells.

### Quantification of target compounds

The target compounds for MS analysis ranged from gaseous, liquid and solid state at room temperature and varied widely in their solubility in H_2_O and selected organic solvents. The different target properties posed an analytical challenge, particularly when it was required to monitor multiple targets in each sample with high-throughput. For this reason, it was necessary to optimize several LC-MS and GC-MS methods on three different mass spectrometers (LC-TripleQuad, GC-QToF and LC-IMS QToF). The linear range and dynamic range of detection for each targeted compound was determined using authentic standards, prior to the analysis of real experimental samples. All compounds for which MS quantification methods were developed are listed in Table S3 along with the MS instrument used. Note: quantification and chiral analysis of mandelic acid was initially performed on the LC-IMS QToF instrument (*Methods 3.4.2*, data presented in Fig.5 and Table 1). Subsequently, more sensitive methods were developed for mandelic acid and hydroxymandelic acid on the LC-Triplequad instrument (*Methods 3.4.1*, data presented in Fig. 6). Targets were first quantified by the rapid non-chiral method before chiral analysis was performed on selected samples.

#### LC-TripleQuad: LC-MS/MS analysis

LC-MS/MS analysis was conducted using an ultra-performance liquid chromatography system (Waters Acquity UPLC H-class) coupled to a Xevo TQ-S triple-quadrupole mass spectrometer (Waters Corporation, MA, USA) equipped with an electrospray ionization source (ESI). The desolvation gas flow rate was set to 1000 L/h at a temperature of 600 °C. The cone gas flow rate was fixed at 150 L/h and the source temperature at 150 °C. The source offset was set to 50 V. The capillary voltage was optimized at 1.0 kV for both positive (ESI+) and negative mode (ESI-). Dwell times of 25 ms/transition were chosen.

The following compounds were monitored in ESI+ mode, with the numbers in brackets indicating the precursor ion mass, product ion mass, cone voltage (V) and collision energy (eV) respectively: Phenylalanine (166.1109, 120.1251, 10 V, 14 eV); Tyrosine (182.06, 136.13, 20 V, 13 eV); L-DOPA (198.1009, 152.1017, 32 V, 12 eV); 3-*O*-methyl-L-DOPA (212.1169, 195.1071, 14 V, 10 eV); Tyramine (138.0195, 121.1586, 2 V, 8 eV).

The following compounds were monitored in ESI- mode, with the numbers in brackets indicating the precursor ion mass, product ion mass, cone voltage (V) and collision energy (eV) respectively: Cinnamic acid (147.0005, 103.0428, 20 V, 10 eV); Coumaric acid (163.0181, 119.0673, 4 V, 14 eV); Caffeic acid (178.9463, 135.0229, 44 V, 12 eV); Ferulic acid (193.092, 134.0974, 30 V, 16 eV); Urocanic acid (137.07, 93.04, 26 V, 12 eV); Coumaraldehyde (147.0839, 41.0244, 54 V, 16 eV); Coniferaldehyde (177.0949, 162.0457, 40 V, 14 eV); Coumaryl alcohol (149.0999, 131.0649, 36 V, 10 eV); Coniferyl alcohol (179.1109, 146.0546, 36 V, 14 eV); Chavicol (133.0411, 105.382, 46 V, 16 eV); Eugenol (163.0521, 148.071, 2 V, 14 eV); Tyrosol (137.0361, 106.143, 42 V, 14 eV); 2-oxoisovaleric acid (115.0151, 71.05, 74 V, 8 eV); Mandelic Acid (151.02, 107.07, 2V, 10 eV); 4-Hydroxymandelic acid (167.07, 123.06, 34 V, 10 eV).

MassLynx v 4.1 (Waters) software was used to process the quantitative data obtained from calibration standards and from diluted samples. The mobile phases, columns, column temperature and gradient programs were set based on the specific chemical properties of the target analytes, as listed below:

1. *Tyrosine, Tyramine and Tyrosol*: A Waters Acquity BEH C18 column (50 mm x 2.1 mm, 1.7 μm) was used at 45 °C, flow rate 0.6 mL/min. An optimum separation gradient was obtained with a binary mobile phase A (H_2_O, 0.01% NH_4_OH) and B (MeOH, 0.01% NH_4_OH). The gradient elution program was: 0-0.5 min, 99-37% A; 0.5-1.0 min, 37-2% A; 1.0-2.0 min, hold at 2% A; 2.0-2.1 min, 2-99% A; 2.1-3.0 min, hold at 99% A. The inject volume was 1 μL.
2. *Cinnamic acid, Coumaric acid, Ferulic acid, Urocanic acid*: A Waters Acquity HSS T3 column (50 mm x 2.1 mm, 1.8 μm) was used at 40 °C, flow rate 0.6 mL/min. An optimum separation gradient was obtained with a binary mobile phase A (H_2_O, 0.01% NH_4_OH) and B (MeOH, 0.01% NH_4_OH). The gradient elution program was: 0-0.5 min, 95-60% A; 0.5-1.0 min, 60-2% A; 1.0-2.0 min, hold at 2% A; 2.0-2.1 min, 2-99% A; 2.1-3.0 min, hold at 99% A. The inject volume was 1 μL.
3. *Tyrosine, L-DOPA, 3-O-methyl-L-DOPA, Coumaric acid, Caffeic acid, Ferulic acid, Coumaryl aldehyde, Coniferyl aldehyde, Coumaryl alcohol, Coniferyl alcohol, Chavicol, Eugenol*: A Waters Acquity HSS T3 column (50 mm x 2.1 mm, 1.8 μm) was used at 40 °C, flow rate 0.6 mL/min. An optimum separation gradient was obtained with a binary mobile phase A (H_2_O, 0.05% NH_4_OH) and B (MeOH, 0.05% NH_4_OH). The gradient elution program was: 0-0.5 min, 99-35% A; 0.5-1.0 min, 35-2% A; 1.0-2.0 min, hold at 2% A; 2.0-2.1 min, 2-99% A; 2.1-3.0 min, hold at 99% A. The inject volume was 1 μL.
4. *2-oxoisovaleric acid*: A Waters Acquity BEH Amide column (50 mm x 2.1 mm, 1.7 μm) was used at 50 °C, flow rate 0.6 mL/min. An optimum separation gradient was obtained with a binary mobile phase A (50/50 MeCN/H_2_O with 10 mM CH_3_COONH_4_ and 0.04% NH_4_OH, pH 9.0) and B (90/10 MeCN/H_2_O with 10 mM CH_3_COONH_4_ and 0.04% NH_4_OH, pH 9.0). The gradient elution program was: 0-0.4 min, 0.1% A; 0.4-0.5 min, 0.1-40% A; 0.5-2.0 min, 40-70% A; 2.0-2.1 min, 70-0.1% A; 2.1-5.0 min, hold at 0.1% A. The inject volume was 1 μL.
5. *Phenylalanine, Tyrosine*: A Waters Acquity HSS T3 column (50 mm x 2.1 mm, 1.8 μm) was used at 40 °C, flow rate 0.6 mL/min. An optimum separation gradient was obtained with a binary mobile phase A (H_2_O, 0.05% NH_4_OH) and B (MeOH, 0.05% NH_4_OH). The gradient elution program was: 0-0.5 min, 95-60% A; 0.5-1.0 min, 60-2% A; 1.0-2.0 min, hold at 2% A; 2.0-2.1 min, 2-95% A; 2.1-3.0 min, hold at 95% A. The inject volume was 1 μL.
6. *Coumaric acid, Cinnamic acid*: A Waters Acquity HSS T3 column (50 mm x 2.1 mm, 1.8 μm) was used at 40 °C, flow rate 0.6 mL/min. An optimum separation gradient was obtained with a binary mobile phase A (H_2_O, 0.01% formic acid) and B (MeOH, 0.01% formic acid). The gradient elution program was: 0-0.5 min, 95-60% A; 0.5-1.0 min, 60-2% A; 1.0-2.0 min, hold at 2% A; 2.0-2.1 min, 2-95% A; 2.1-3.0 min, hold at 95% A. The inject volume was 1 μL.
7. *Mandelic acid, 4-Hydroxymandelic acid*: Non-chiral quantification. A Waters Acquity HSS T3 column (50 mm x 2.1 mm, 1.8 μm) was used at 40 °C, flow rate 0.6 mL/min. An optimum separation gradient was obtained with a binary mobile phase A (H_2_O, 0.005% NH_4_OH) and B (MeOH, 0.005% NH_4_OH). The gradient elution program was: 0-0.5 min, 98% A; 0.5-0.8 min, 98-2% A; 0.8-1.2 min, hold at 2% A; 1.2-1.3 min, 2-98% A; 1.3-2.0 min, hold at 98% A. The inject volume was 2 μL.
8. *Mandelic acid, 4-Hydroxymandelic acid*: Chiral analysis. An Astec CHIROBIOTIC T column (250 mm X 4.6 mm, 5 μm) was used at 25 °C, flow rate 0.5 mL/min. An optimum separation gradient was obtained with a binary mobile phase A (H2O, 0.05% formic acid) and B (MeOH, 0.05% formic acid). The gradient elution program was: 0-12 min, 99% A; 12-13 min, 98-2% A; 13-17 min, hold at 2% A; 17-18 min, 2-90% A; 18-25 min, hold at 90% A. The inject volume was 0.5 μl.

#### LC-IMS QToF: LC-MS analysis

Further LC-MS based analysis was undertaken using an Agilent 1290 infinity II UHPLC system coupled to an Agilent 6560 IMS QToF equipped with an ESI source in negative ion mode with the following settings: VCap 3000 V; fragmentor 175 V; gas temperature 300 °C; sheath gas temperature 350 °C; drying gas flow 10 L/min; nebulizer 50 psi; and sheath gas flow 12 L/min.

1. *Phenylpyruvic acid, phenylglyoxylic acid, (S)-Mandelic acid and (R)-Mandelic acid*: A Chiralpak IB-U column (50 mm x 3.0 mm, 1.6 μm) (Daicel Corp) was used at 45 °C with varying flow rate. An optimum separation gradient was obtained with a binary mobile phase A (H_2_O, 0.1% formic acid) and B (Isopropanol, 0.1% formic acid). The elution program was: 0-10 min, hold 99% A at 0.1 mL/min; 10-10.5 min, 99-5% A at 0.3-0.5 mL/min; 10.5-11 min, hold 5% A at 0.5 mL/min; 11-11.5 min, 5-99% A at 0.5 mL/min; 11.5-12 min, 99% A at 0.5-0.1 mL/min. The inject volume was 5 μL.
2. *cis,cis-Muconic acid and trans,trans-Muconic acid*: A Waters Acquity BEH Amide column (50 mm x 2.1 mm, 1.7 μm) was used at 40 °C with varying flow rate. An optimum separation gradient was obtained with a binary mobile phase A (H_2_O, 0.1% formic acid) and B (Acetonitrile, 0.1% formic acid). The elution program was: 0-1.0 min, hold 99% A at 0.6 mL/min; 1.0-2.0 min, 99%-97% A at 0.2 mL/min; 2.0-2.1 min, 97-5% A at 0.2-0.6 mL/min; 2.1-2.9 min, hold 5% A at 0.6 mL/min; 2.9-3.0 min, 5-99% A at 0.6 mL/min; 3.0-4.0 min, hold 99% A at 0.6 mL/min.

Data was analyzed within MassHunter proprietary software (Agilent), where raw peak areas were calculated and quantification of the targets was conducted by extrapolating the target peak area against a generated standard curve.

#### GC-QToF: GC-MS sample preparation and analysis

GC-MS analysis was used to quantify volatile targets (styrene, 4-vinylphenol, 4-vinylimidazole, 4-vinylguaiacol, butadiene and pentadiene). Culture media was overlaid with TMP, and both this organic layer and the aqueous culture media were recovered separately, then diluted 20x or 50x in TMP. Following dilution, the samples were vortexed with anhydrous MgSO_4_ to remove residual water, then 100 µl of the dry samples were transferred to 200 µl MS vials for immediate analysis. For quantification of 4-vinylimidazole, the analyte partitioned fully into the aqueous phase, from which a 20x dilution in methanol was prepared. *Sec*-butylbenzene (Sigma) was used as an internal standard to allow for accurate quantification. 0.005% v/v *sec*-butylbenzene was added to all samples and the quantification of the respective target was calculated relative to the peak area of this internal standard. In addition, an eight-point calibration curve was constructed in the range of 0-200 mg/L in the appropriate solvent/matrix to the samples.

GC-MS targets were analyzed on an Agilent Technologies 7200 accurate mass Q-TOF MS coupled to a 7890B GC with a PAL RSI 85 autosampler and an Agilent VF-5ms column (30 m x 250 μm x 0.25 μm). The MS was equipped with an electron impact ion source using 70 eV ionization and a fixed emission of 35 μA. Mass spectra were collected for the range of 35-500 mz with an acquisition rate of 5 spectra/s and an acquisition time of 200 ms/spectrum.

1. *Vinylbenzene analytes*: The sample (1 μL) was analyzed with an inlet temperature of 280 °C and a split ratio of 10:1. Helium was used as the carrier gas with a flow rate of 1.5 mL/min and a pressure of 16.2 psi. The chromatography was programmed to begin at 100 °C with a hold time of 1 min, followed by an increase to 178 °C at a rate of 80 °C/min, a subsequent increase to 182 °C at a rate of 5 °C/min, followed by an increase to 325 °C at a rate of 120 °C/min and a final hold time of 1 min. The total runtime per analysis was 4.967 minutes.
2. *Diene analytes (Butadiene & Pentadiene)*: The sample (1 μL) was analyzed with an inlet temperature of 250 °C and a split ratio of 25:1. Helium was used as the carrier gas with a flow rate of 1 mL/min and a pressure of 7 psi. The chromatography was programmed to begin at 40 °C with a hold time of 2 min, followed by an increase to 140 °C at a rate of 40 °C/min, followed by an increase to 280 °C at a rate of 120 °C/min, and a final hold time of 0.5 min. The total runtime per analysis was 6.167 minutes.

Q-TOF vendor binary files were converted to open source mzXML data format (Pedrioli et al., 2004) using ProteoWizard msConvert (Chambers et al., 2012). Automated target quantitation was conducted using in-house scripts written in R to automatically extract relevant peak areas. Quantification of the targets was conducted by extrapolating the target/*sec*-butylbenzene peak area against a generated standard curve.

## Learn

#### Plasmid vector optimization

To optimize mandelic acid production, the SyHMAS gene was subcloned into different expression vectors from the BglBrick library of plasmids (Lee et al., 2011). Plasmid backbone screening was performed following an approach based on statistical learning. Starting from an initial combinatorial library of 48 plasmid variants (4 replication origins, 4 promoter types, 3 antibiotic resistance genes), a 12-member library was selected using DoE. The DoE library was generated using JMP Pro 12.2.0 (SAS Institute Inc.), through a custom script design based on the D-optimality criterion for design efficiency.

The 12 member plasmid library was constructed and experimental titers of the mandelic acid target were quantified, then fitted to a model through ordinary least square contrast regression analysis using the Statsmodels Python package (Seabold & Perktold 2010). More precisely, each of the factors that were varied in the experimental design, i.e. plasmid copy number, promoter strength, and antibiotic resistance, were considered in the regression analysis as categorical variables in order to fit the model against the production titers. Mandelic acid titers were then predicted for the full factorial library of 48 combinations. A further 6 plasmids were then selected (to include predicted high, medium and low producers), constructed and tested experimentally for mandelic acid titers to validate the model.

# References

Baba T, *et al*. Construction of *Escherichia coli* K-12 in-frame, single-gene knockout mutants: the Keio collection. *Mol. Syst. Biol.* **2**, 2006.0008 (2006)

Bramucci MG, Nagarajan V, & Thomas SM. Use of xylene monooxygenase for the oxidation of substituted monocyclic aromatic compounds. Patent: WO2003014368A2 (2001).

Carbonell P, *et al.* Selenzyme: enzyme selection tool for pathway design. *Bioinformatics* **34,** 2153–2154 (2018a).

Carbonell P, *et al.* An automated Design-Build-Test-Learn pipeline for enhanced microbial production of fine chemicals. *Commun. Biol.* **1,** 66 (2018b).

Chambers MC, *et al.* A cross-platform toolkit for mass spectrometry and proteomics. *Nat. Biotechnol.* **30**, 918-920 (2012).

Cherepanov PP, & Wackernagel W. Gene disruption in *Escherichia coli*: TcR and KmR cassettes with the option of Flp-catalyzed excision of the antibiotic-resistance determinant. *Gene* **158**, 9-14 (1995).

Chung D, Kim SY, & Ahn JH. Production of three phenylethanoids, tyrosol, hydroxytyrosol, and salidroside, using plant genes expressing in *Escherichia coli. Sci. Rep*. **7**, 2578 (2017).

Currin A, *et al*. GeneORator: An Effective Strategy for Navigating Protein Sequence Space More Efficiently through Boolean OR-Type DNA Libraries. *ACS Synth. Biol.* **8**, 1371-1378 (2019a).

Currin A, *et al*. Highly multiplexed, fast and accurate nanopore sequencing for verification of synthetic DNA constructs and sequence libraries. *Synth. Biol.* **4**, ysz025 (2019b).

Datsenko KA, & Wanner BL. One-step inactivation of chromosomal genes in Escherichia coli K-12 using PCR products. *Proc Nat Ac Sci*, **97**, 6640-6645 (2000).

Datta S, Costantino N, & Court DL. A set of recombineering plasmids for gram-negative bacteria. *Gene*. **379**, 109–115 (2006).

Delépine B, Duigou T, Carbonell P, & Faulon JL. RetroPath2.0: A retrosynthesis workflow for metabolic engineers. *Metab. Eng.* **45,** 158–170 (2018).

Duigou T, du Lac M, Carbonell P, & Faulon JL. RetroRules: a database of reaction rules for engineering biology. *Nucleic Acids Res.* **47,** D1229–D1235 (2019).

Espah Borujeni A, Channarasappa AS, & Salis HM. Translation rate is controlled by coupled trade-offs between site accessibility, selective RNA unfolding and sliding at upstream standby sites. *Nucleic Acids Res*. **42**, 2646-59 (2014).

Goldfeder M, Kanteev M, Adir N, & Fishman A. Influencing the monophenolase/diphenolase activity ratio in tyrosinase. *Biochim. Biophys. Acta.* **1834**, 629-633 (2013).

Goos P, & Jones B. Optimal design of experiments: a case study approach. *Wiley* (2011). ISBN: 978-0-470-74461-1

Ham TS, Dmytriv Z, Plahar H, Chen J, Hillson NJ, & Keasling JD. Design, implementation and practice of JBEI-ICE: an open source biological part registry platform and tools. *Nucleic Acids Res*. **40**, e141 (2012).

Kikuchi Y, Tsujimoto K, & Kurahashi O. Mutational analysis of the feedback sites of phenylalanine-sensitive 3-deoxy-D-arabino-heptulosonate-7-phosphate synthase of *Escherichia coli*. *Appl. Environ. Microbiol.* **63**, 761-762 (1997).

Lawson AJ, Swienty-Busch J, Géoui T, & Evans D. The making of Reaxys – Towards unobstructed access to relevant chemistry information. In *The Future of the History of Chemical Information*; McEwen, L. R.; Buntrock, R. E., Eds.; *ACS Symposium Series*. **1164**, 127–148 (2014).

Lee TS, *et al*. BglBrick vectors and datasheets: A synthetic biology platform for gene expression. *J. Biol. Eng.* **5**, 12 (2011).

Luo ZW, & Lee SY. Biotransformation of *p*-xylene into terephthalic acid by engineered *Escherichia coli*. *Nat. Commun.* **8**, 15689 (2017).

Lütke-Eversloh T, & Stephanopoulos G. Feedback inhibition of chorismate mutase/prephenate dehydrogenase (TyrA) of Escherichia coli: generation and characterization of tyrosine-insensitive mutants. *Appl. Environ. Microbiol*. **71**, 7224-7228 (2005).

Nelms J, Edwards RM, Warwick J, & Fotheringham I. Novel mutations in the *pheA* gene of *Escherichia coli* K-12 which result in highly feedback inhibition-resistant variants of chorismate mutase/prephenate dehydratase. *Appl. Environ. Microbiol.* **58**, 2592-2598 (1992).

Pedrioli PG, *et al*. A common open representation of mass spectrometry data and its application to proteomics research. *Nature Biotechnol*. **22**, 1459-1466 (2004).

Robinson CJ, *et al*. Multifragment DNA Assembly of Biochemical Pathways via Automated Ligase Cycling Reaction. *Methods Enzymol*. **608**, 369-392 (2018).

Sambrook J, & Russell D. *Molecular Cloning: A Laboratory Manual*, 3rd edn. Cold Spring Harbor, NY: Cold Spring Harbor Laboratory Press (2001).

Sadler JC, Green L, Swainston N, Kell DB, & Currin A. Fast and Flexible Synthesis of Combinatorial Libraries for Directed Evolution. *Methods Enzymol.* **608**, 59-79 (2018).

Seabold S, & Perktold J. Statsmodels: econometric and statistical modeling with Python. *Proc. of the 9th Python in Science Conf.* (SCIPY 2010)

Sukumar N, Xu Y, Gatti DL, Mitra B, & Mathews FS. Structure of an active soluble mutant of the membrane-associated (*S*)-mandelate dehydrogenase. *Biochemistry*. **40**, 9870-9878 (2001).

Swainston N, *et al*. PartsGenie: an integrated tool for optimizing and sharing synthetic biology parts. *Bioinformatics*. **34**, 2327-2329 (2018).

Weaver LM, & Herrmann KM. Cloning of an *aroF* allele encoding a tyrosine-insensitive 3-deoxy-D-arabino-heptulosonate 7-phosphate synthase. *J. Bacteriol.* **172**, 6581-6584 (1990).


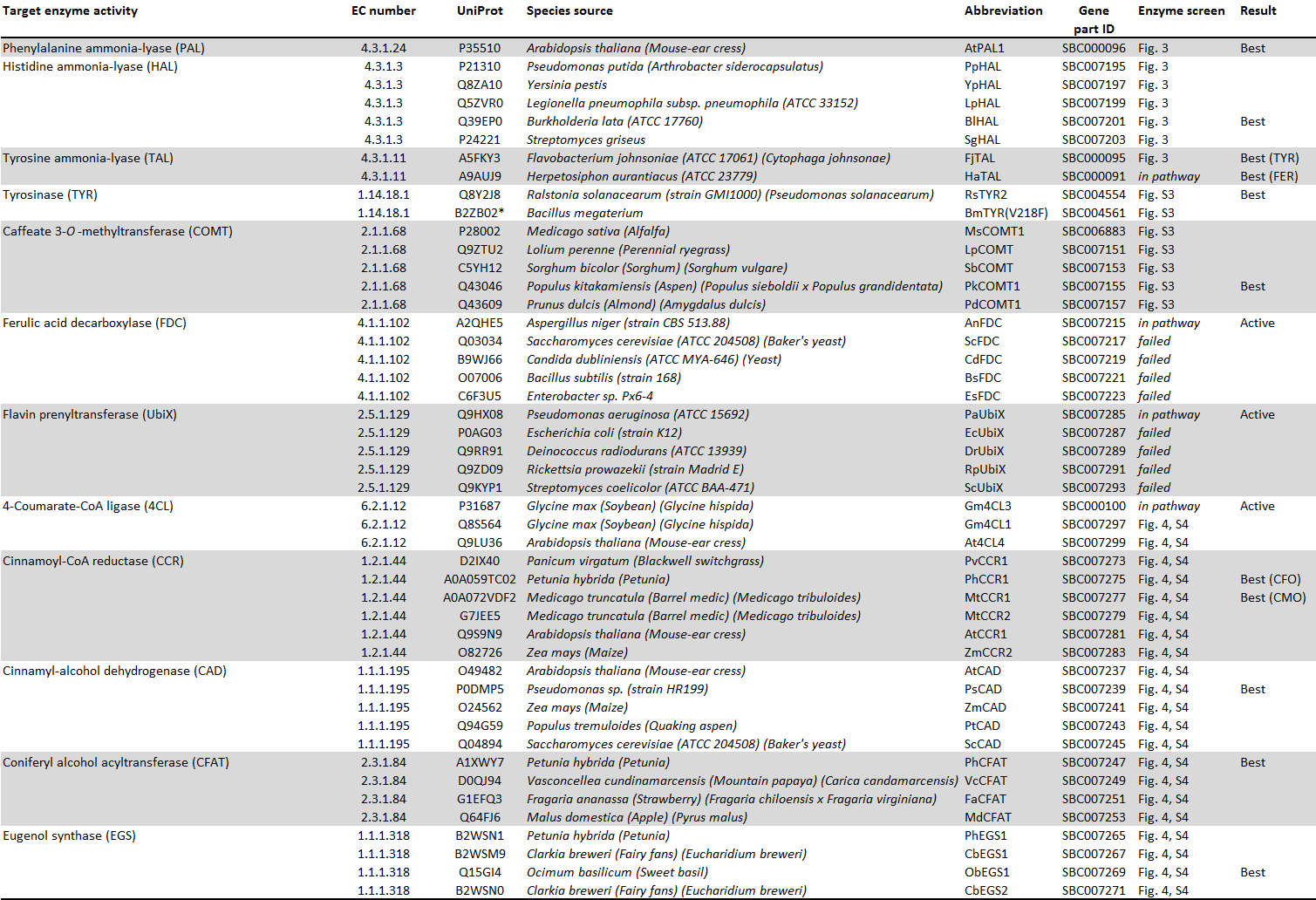


**Table S1**. Summary of enzyme candidates and screening results. The enzyme sequences used are identical to the UniProt IDs presented, except for BmTYR(V218F) which has a single amino acid mutation to enhance monophenolase/diphenolase activity (Goldfeder et al., 2013). *(continues on next page…)*


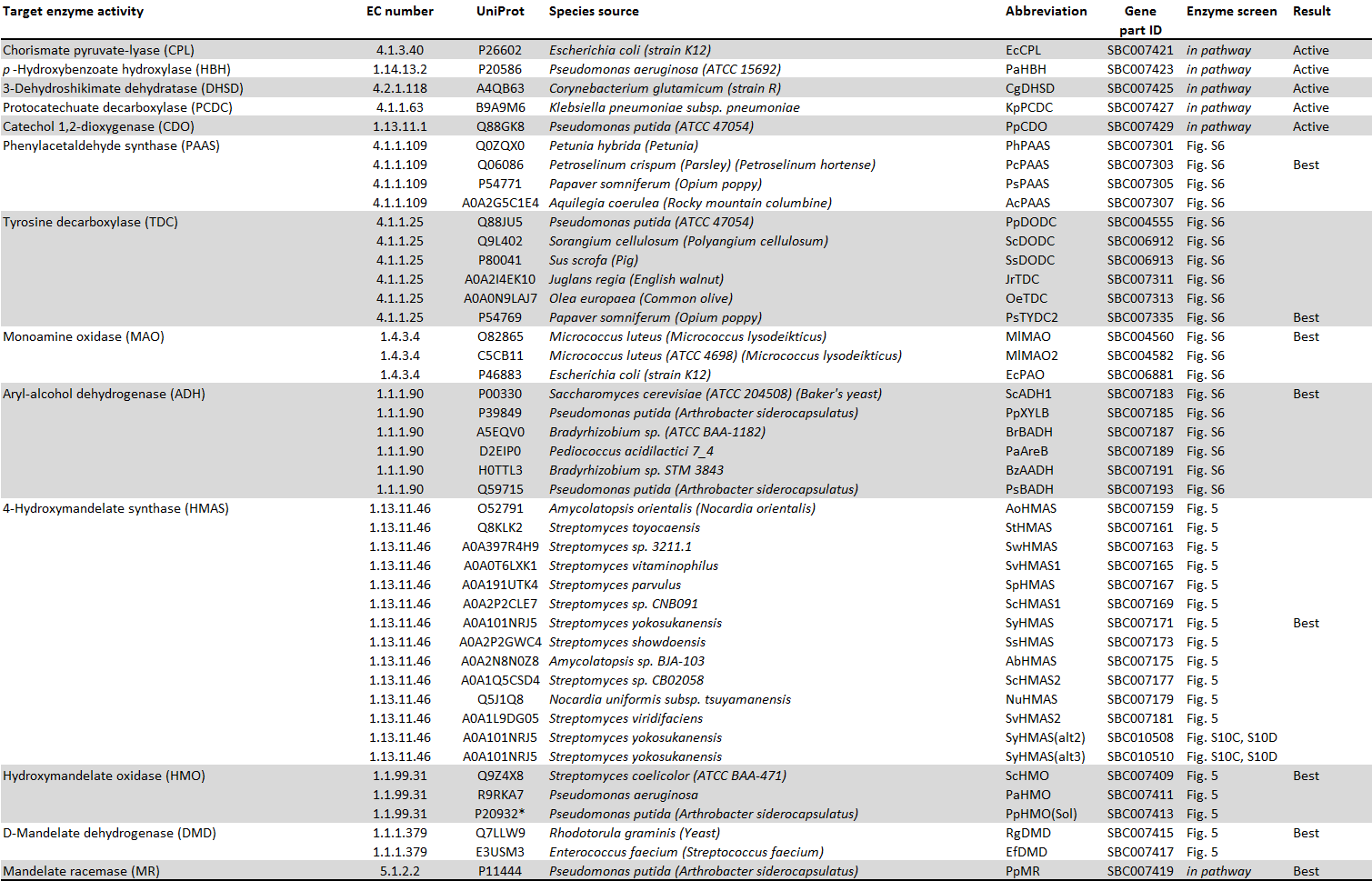


**Table S1**. Summary of enzyme candidates and screening results (*continued*). The enzyme sequences used are identical to the UniProt IDs presented, except for PpHMO(Sol) which has been modified to aid solubility (Sukumar et al., 2001). *(continues on next page…)*


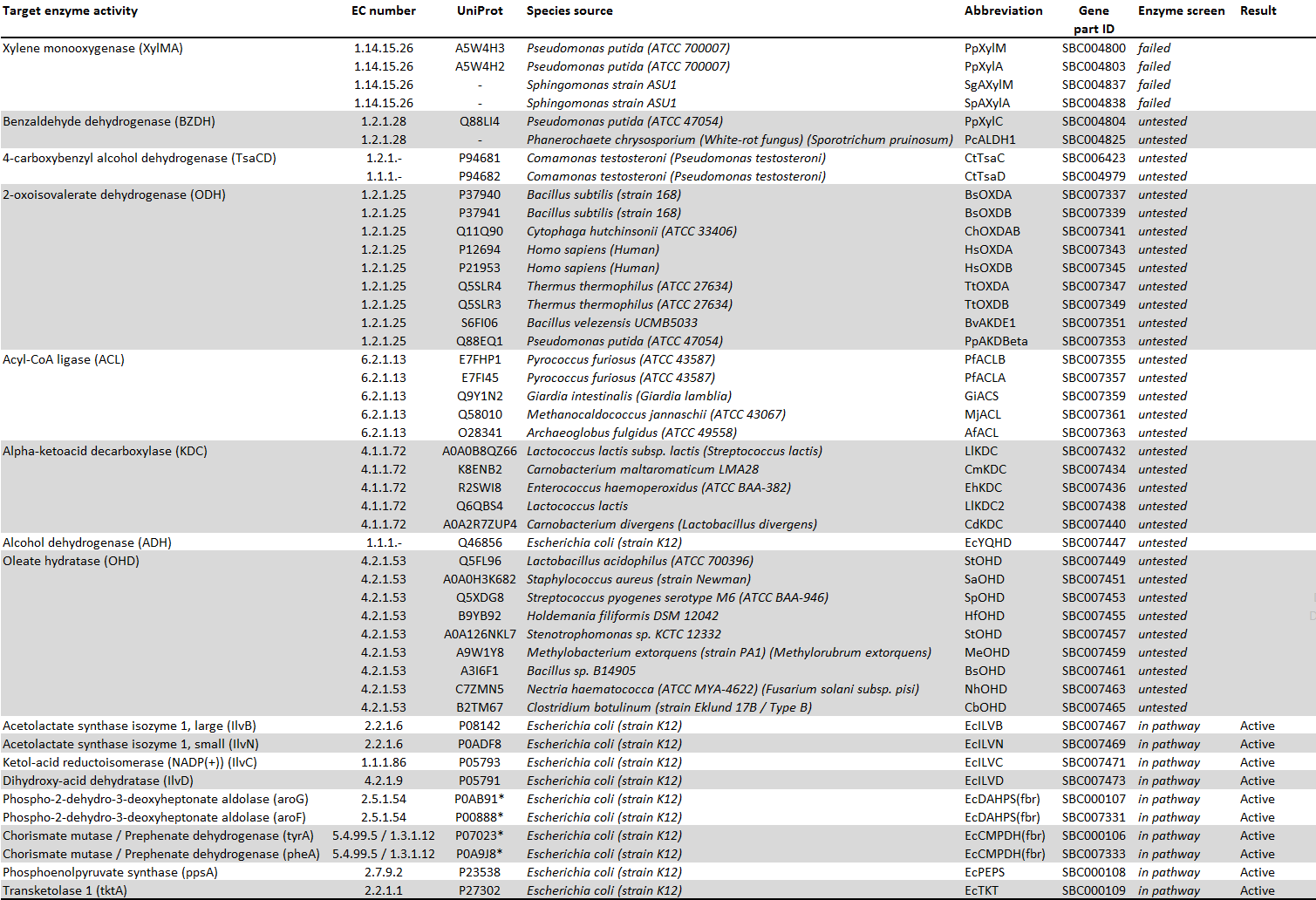


**Table S1**. Summary of enzyme candidates and screening results (*continued*). The enzyme sequences used are identical to the UniProt IDs presented, except for feedback resistant (fbr) mutations of EcDAHPS and EcCMPDH (Weaver & Herrmann, 1990; Nelms et al., 1992; Kikuchi et al., 1997; Lütke-Eversloh & Stephanopoulos, 2005). SgAXylM and SgAXylA sequences were from Bramucci *et al*. (2001).


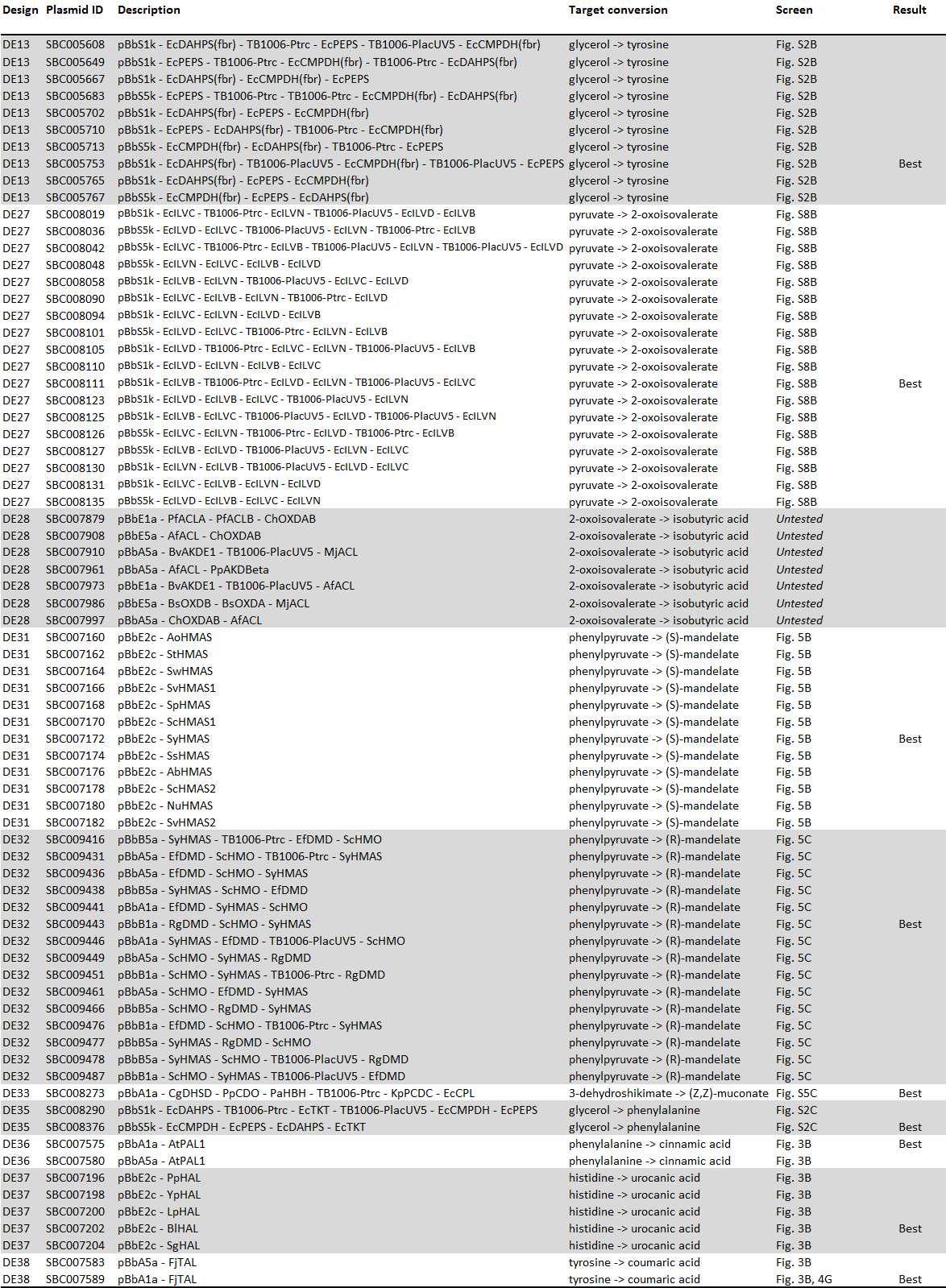


**Table S2**. Summary of plasmid constructs and screening results. Listed plasmids were constructed and sequence verified as described (*Methods 2.3, Pathway assembly*). Plasmids are grouped by design (DE) number. Figure references indicate were the screening data is presented. *(continues on next page…)*


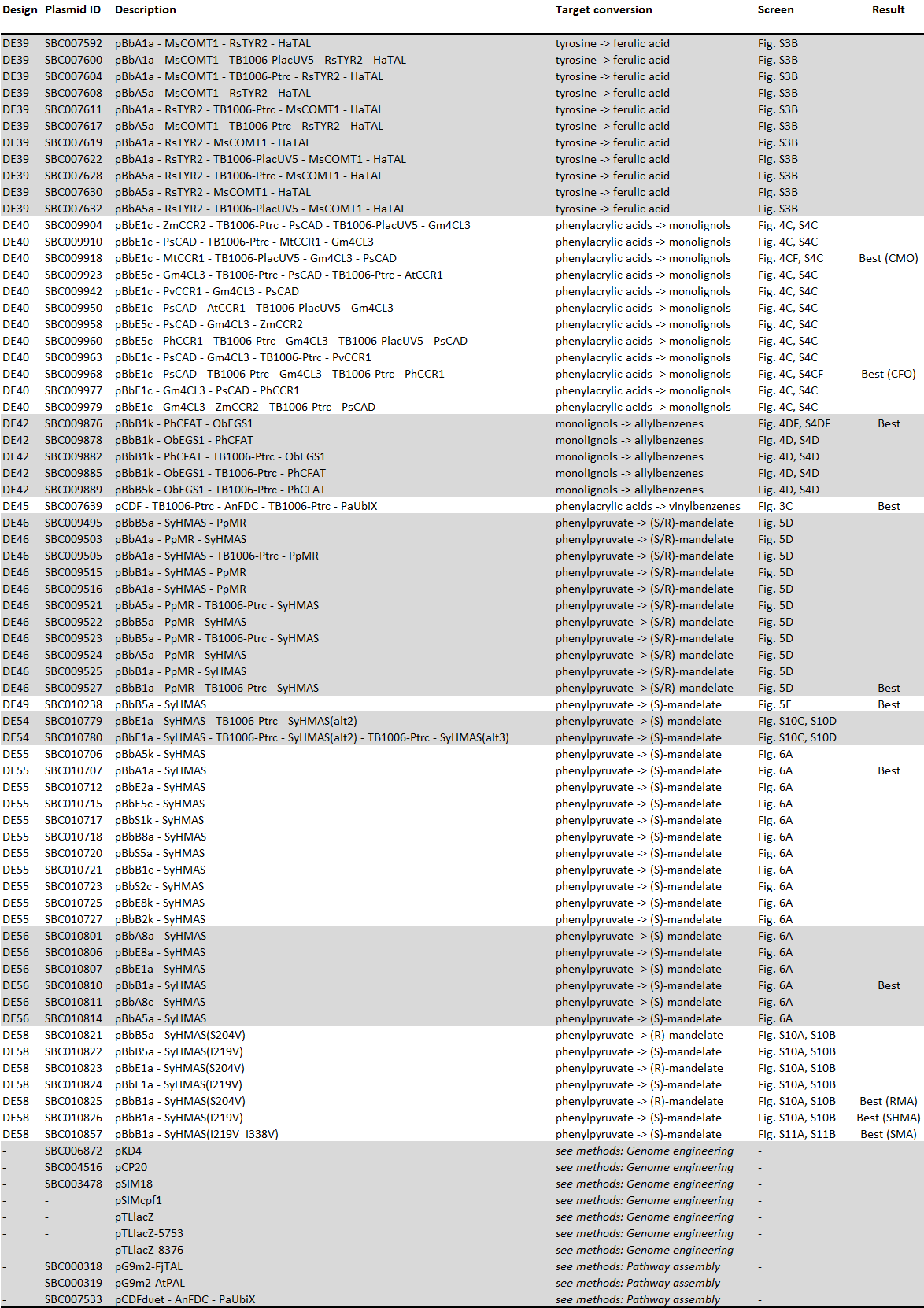


**Table S2**. Summary of plasmid constructs and screening results (*continued*). Listed plasmids were constructed and sequence verified as described (*Methods 2.3, Pathway assembly*). Plasmids are grouped by design (DE) number. Figure references indicate were the screening data is presented.


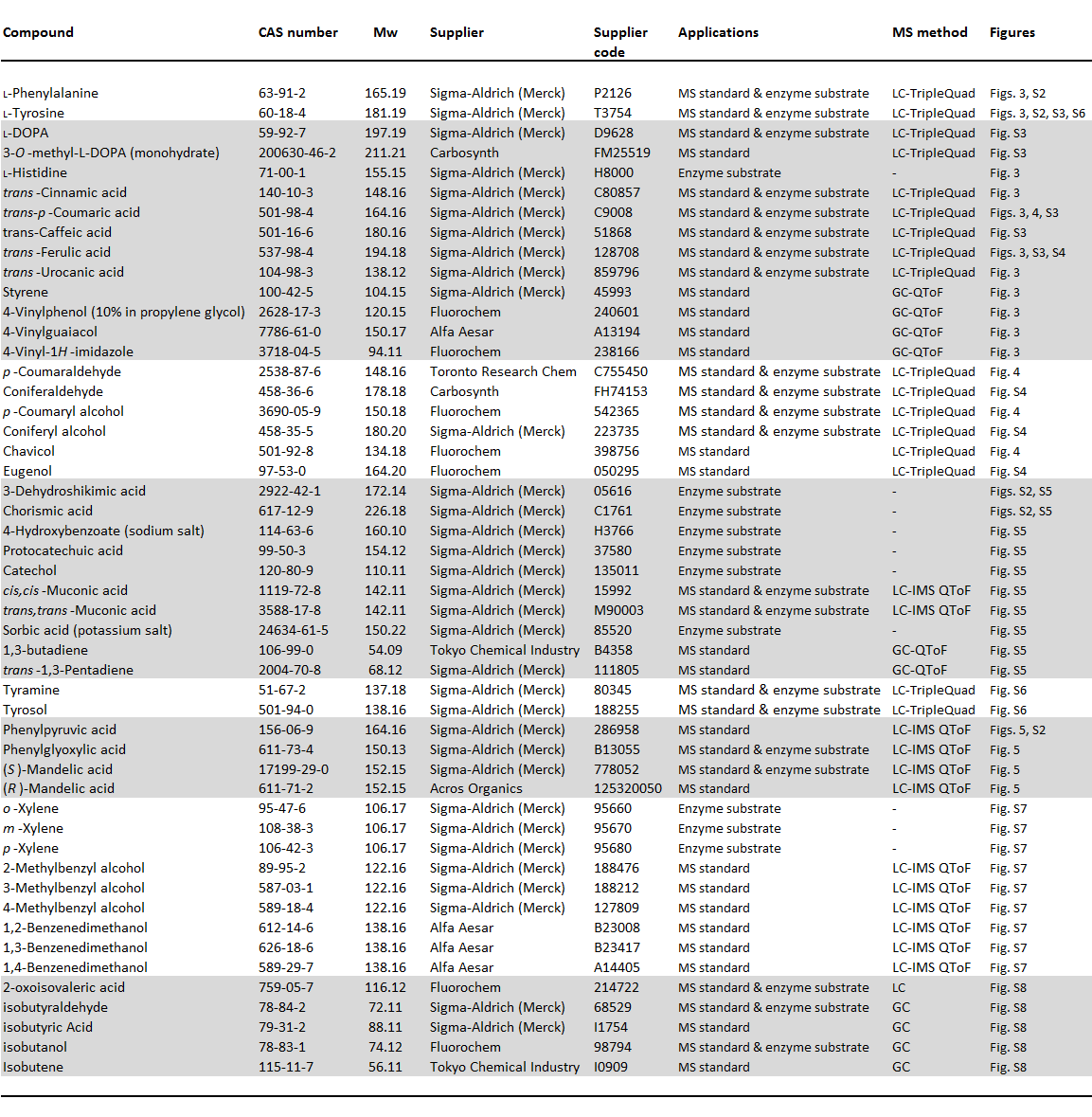


**Table S3**. Chemical standards and substrates. Listed compounds were used as standards for quantification by mass spectrometry (*Methods 3.4, Quantification of target compounds*) and as substrates for enzymes/pathways, as indicated. Supplier and supplier codes are provided for each compound purchased.


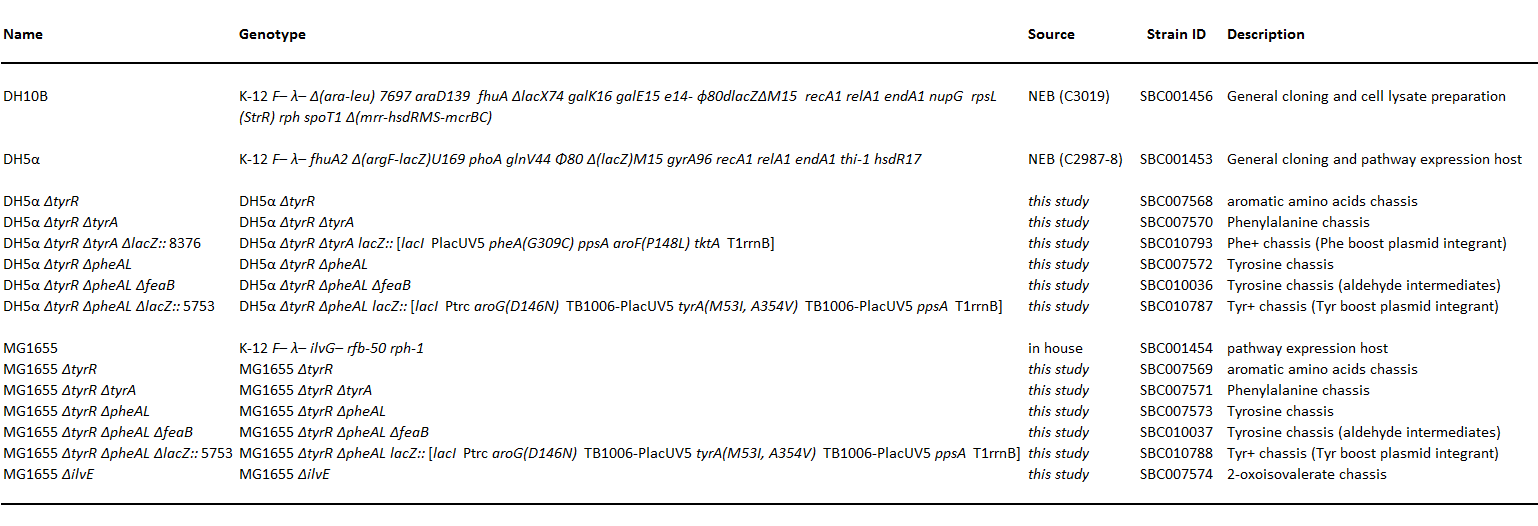


**Table S4**. *E. coli* strains used in this study. DH5α and MG1655 gene knockouts and plasmid integrants were prepared as described (*Methods 2.2, Genome engineering*) and verified by DNA sequencing.


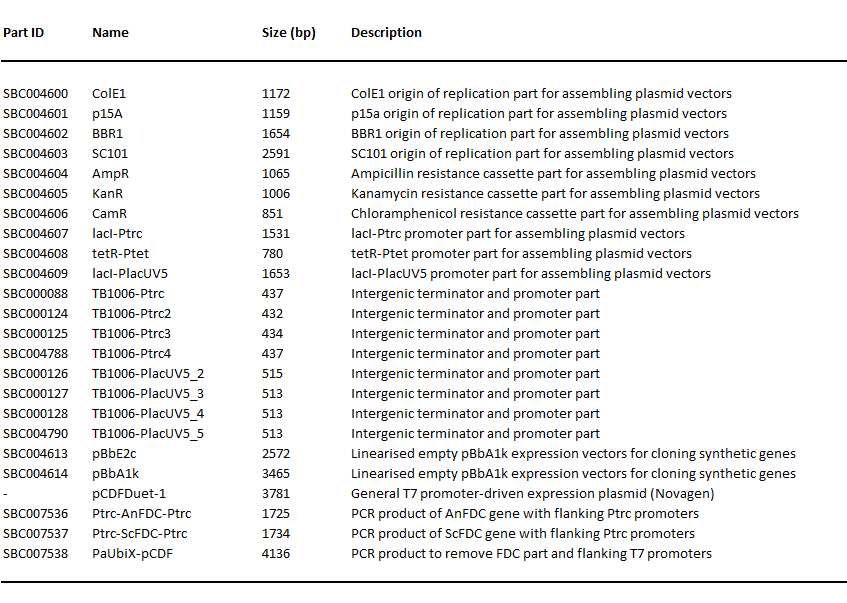


**Table S5**. General DNA parts for construction of plasmid constructs. Listed parts were used to create plasmid vector backbones during automated LCR or manual assembly of pathways, along with the gene parts listed in Table S1.


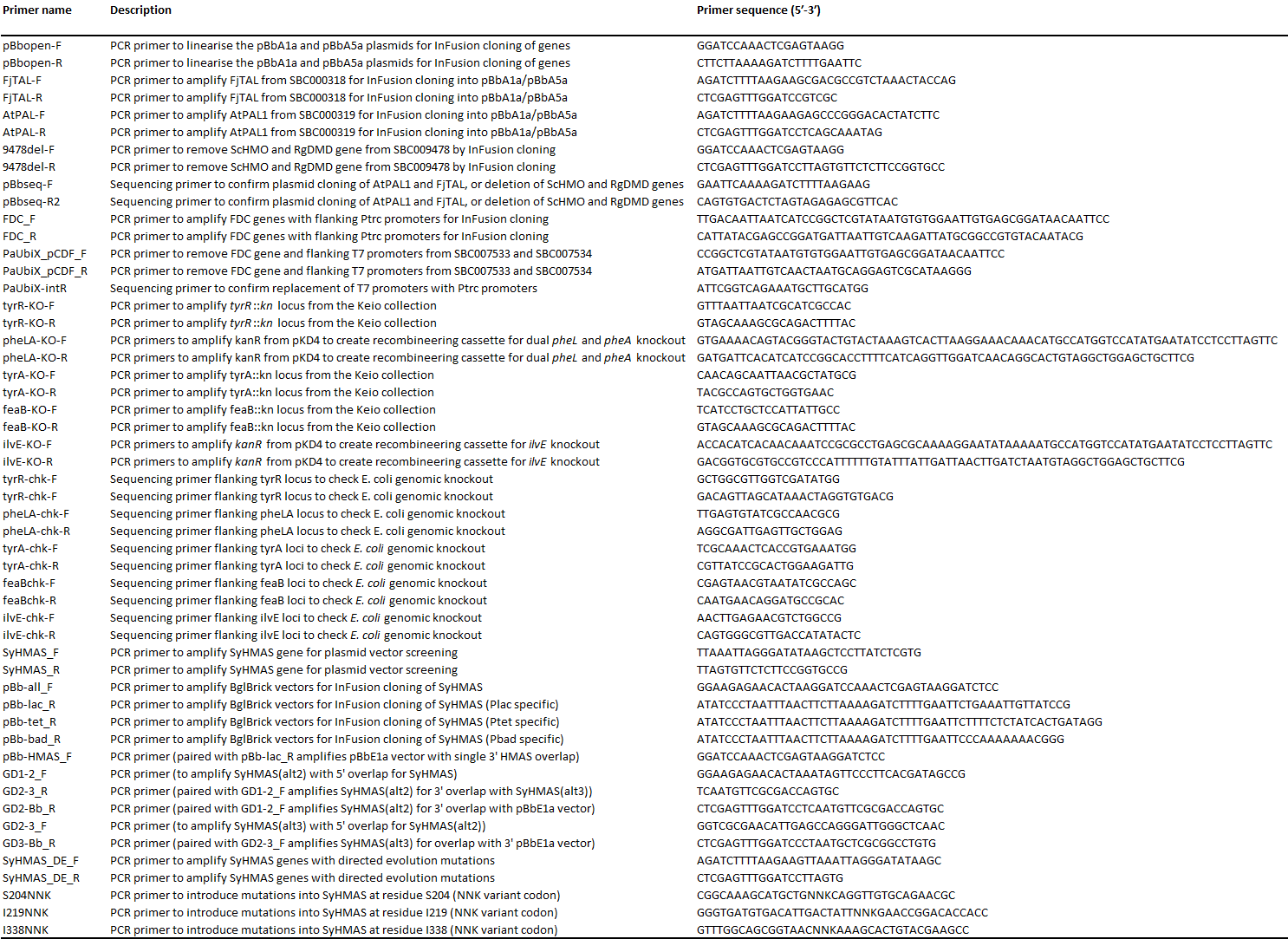


**Table S6**. DNA primers used in this study. Listed primers were used for PCR preparation of parts for In-Fusion cloning, for DNA sequencing, or for targeted gene knockouts in *E. coli*, or for mutagenesis of SyHMAS (see *Methods 2.2-2.4* for details). PCR primers for LCR part preparation and bridging oligonucleotides for LCR assembly of plasmids are listed in the separate ‘*Design of Experiments*’ document.

A

**
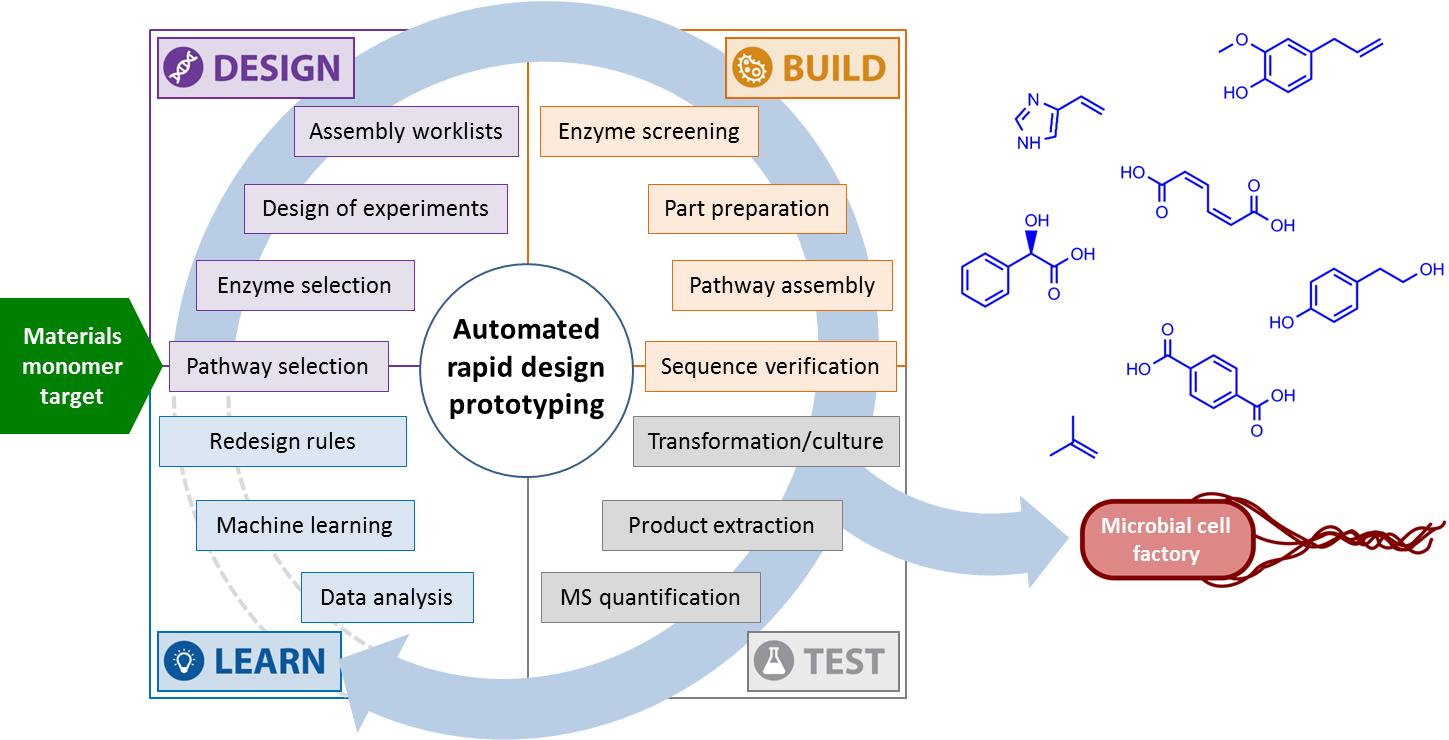
**

B

**
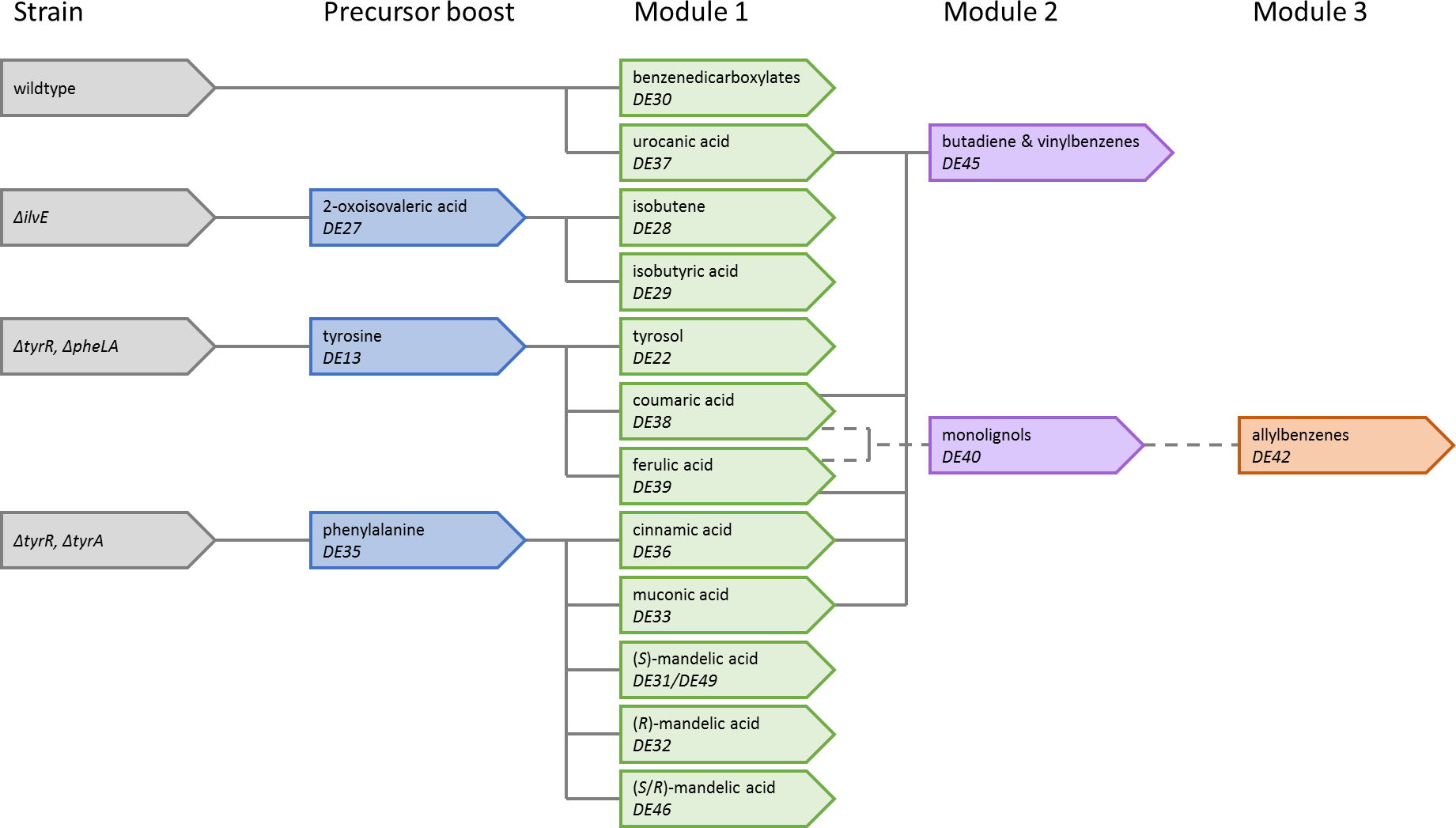
**

**Figure S1**: The DBTL cycle for microbial production of materials monomers. (**A**) The cycle starts at the Design stage (purple) with *in silico* tools for pathway and enzyme selection. Gene parts are sequence optimized, and combined with regulatory DNA parts into plasmid libraries through *Design of Experiments*, and automated assembly worklists are generated. The Build stage (orange) starts with enzyme screening in cell lysates, to identify the best performing candidates, which are then prepared as DNA parts for automated assembly into biosynthetic pathway libraries via ligase cycling reaction. Assembled constructs are then sequence verified through next-generation sequencing. The Test stage (grey) encompasses the transformation of microbial host strains with the biosynthetic pathway constructs, cell growth and induction of enzyme expression, automated product extraction, and target quantification via optimized MS methods. Results are analyzed at the Learn stage (blue) through predictive models using statistical methods and machine learning to inform further rounds of design. (**B**) Modular design of target production strains. *E. coli* host strains were engineered through gene knockouts to support production of multiple targets. Plasmids were then designed to increase flux towards metabolite precursors for the target pathways. Biosynthetic pathways were split into modules to increase assembly efficiency, aid troubleshooting and enable sharing of modules between pathways. DE numbers refer to the plasmid construct libraries generated through our *Design of Experiments* tools (see methods sections 1.4).


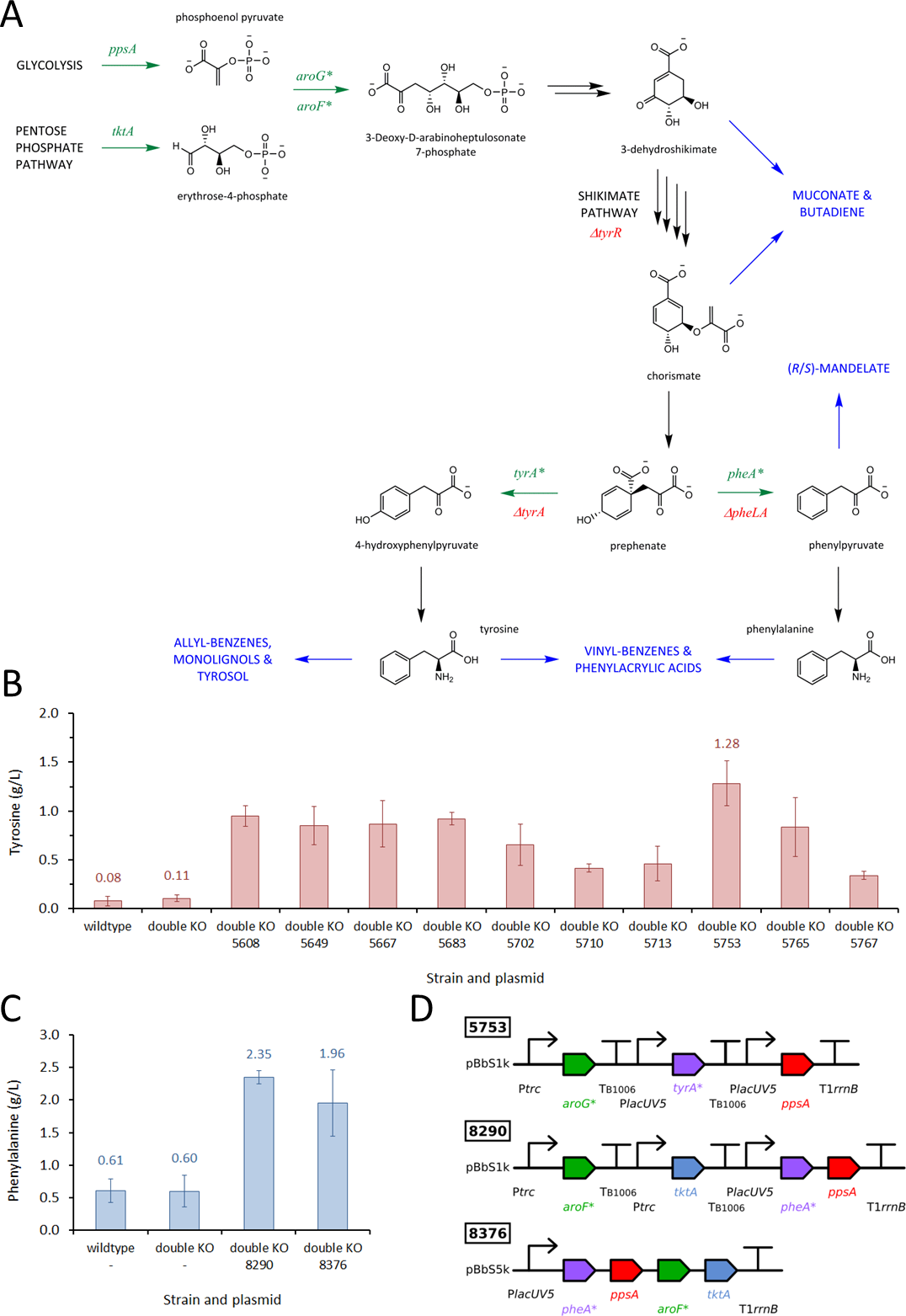


**Figure S2**: (**A**) Metabolic engineering of *E. coli* cells to increase flux down the shikimate pathway towards tyrosine and phenylalanine. Blue arrows indicate where this native pathway interfaces with biosynthetic pathways to material monomer targets. Enzymes highlighted in green were over-expressed from plasmid constructs, whilst those in red were deleted from the host genome. Genes marked with asterisks were mutated to relax feedback inhibition. Genes encode the following enzymes: *ppsA* (phosphoenolpyruvate synthase); *tktA* (transketolase 1); *aroG** (phospho-2-dehydro-3-deoxyheptonate aldolase, Phe-sensitive, with D146N mutation); *aroF** (phospho-2-dehydro-3-deoxyheptonate aldolase, Tyr-sensitive, with P148L mutation); *tyrA** (chorismate mutase/prephenate dehydratase, with M53I and A354V mutations); and *pheA** (chorismate mutase/prephenate dehydratase, with G309C mutation). To boost tyrosine production, *ppsA* and *tktA* were expressed with *aroG** and *tyrA** in *ΔtyrR*, *ΔpheLA* DH5α cells. To boost phenylalanine production, *ppsA* and *tktA* were expressed with *aroF** and *pheA** in *ΔtyrR, ΔtyrA* mutant DH5α cells. (**B**) Screening tyrosine pathway constructs in DH5α double KO (*ΔtyrR, ΔpheLA*) cells growing in TBP media with 0.4% glycerol. After 24 hr, SBC005753 was the most productive plasmid (1.28 g/L) in the double KO host, producing 16x more tyrosine than the wildtype (WT) strain. (**C**) Screening phenylalanine pathway constructs in DH5α double KO (*ΔtyrR, ΔtyrA*) cells growing in TBP media with 0.4% glycerol. After 24 hr, SBC008290 and SBC008376 produced 2.35 g/L and 1.96 g/L phenylalanine respectively in the double KO host, 3.85x and 3.21x more phenylalanine than the wildtype (WT) strain. (**D**) Best performing plasmid constructs.


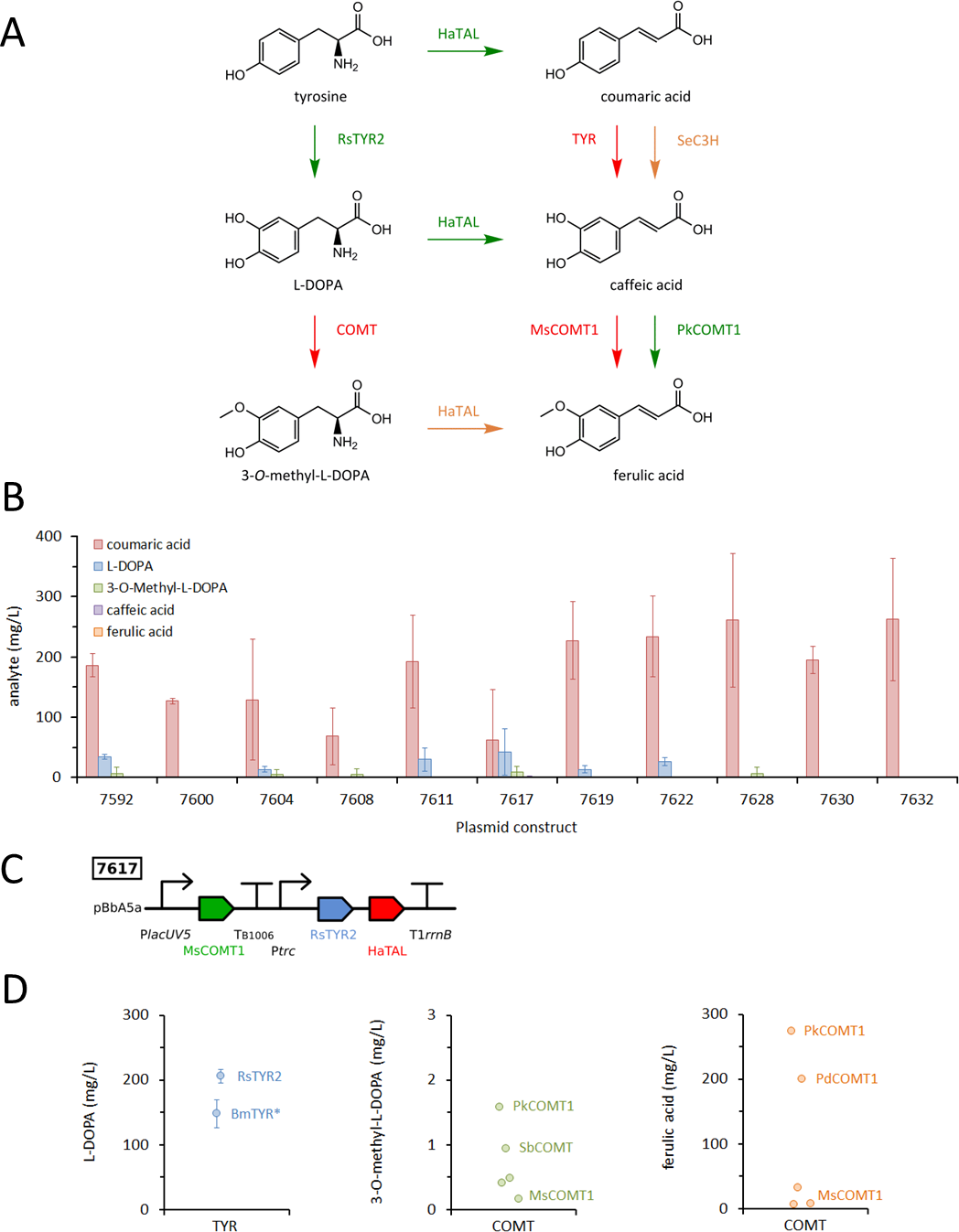


**Figure S3**: (**A**) Pathways from tyrosine to ferulic acid. Enzyme abbreviations: HaTAL (tyrosine ammonia-lyase from *Herpetosiphon aurantiacus*); TYR (tyrosinase); RsTYR2 (TYR from *Ralstonia solanacearum*); COMT (caffeic acid *O*-methyltransferase); MsCOMT (COMT from *Medicago sativa*); PkCOMT1 (COMT from *Populus kitakamiensis*); and SeC3H (4-coumarate 3-hydroxylase from *Saccharothrix espanaensis*). Multiple potential routes are shown: green arrows indicate experimentally verified activity (this study), orange arrows indicate potential activity (not tested), red arrows indicate no observed activity (this study). (**B**) Screening ferulic acid pathway constructs in DH5α cells fed 3 mM tyrosine. After 24 hr, no detectable ferulic acid was produced by any construct. (**C**) The best performing plasmid construct SBC007617 produced the most 3-*O*-methyl-L-DOPA (~10 mg/L) and caffeic acid (~1 mg/L). (**D**) Subsequent screening of enzyme candidates in cell lysates with 3 mM substrate. HaTAL and PkCOMT1 showed good activity with tyrosine and caffeic acid as substrates, respectively. We suspect that HaTAL activity outcompetes RsTYR2 *in vivo*, channeling the majority of tyrosine substrate into the dead-end product coumarate. Replacing RsTYR2 with SeC3H should reconstitute a sequential 3-step pathway to ferulic acid.


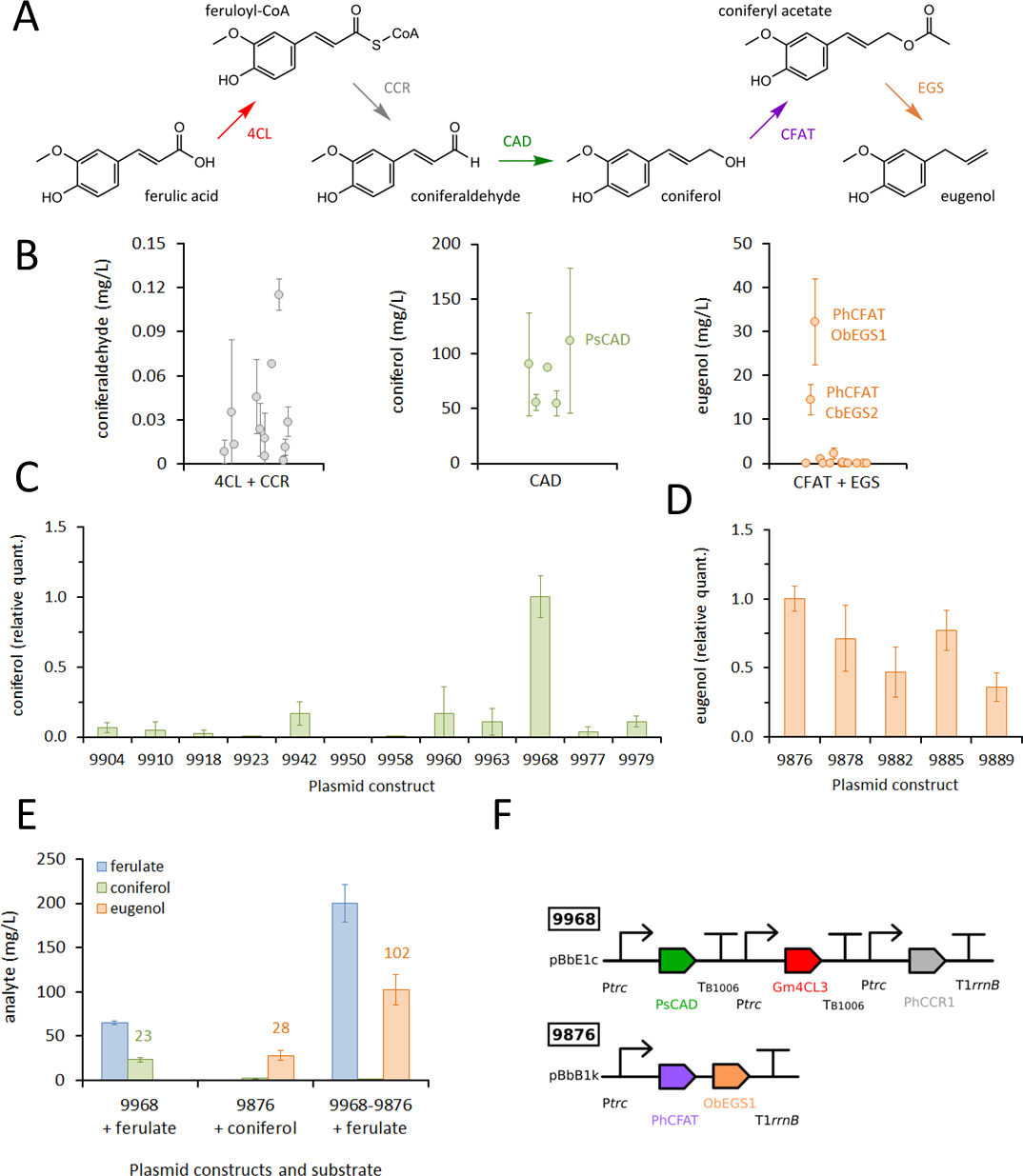


**Figure S4**: (**A**) Pathway from ferulate to coniferol and eugenol. Enzyme abbreviations: 4CL (4-coumarate-CoA ligase); CCR (cinnamoyl-CoA reductase); CAD (cinnamyl alcohol dehydrogenase); CFAT (coniferyl alcohol acyltransferase); and EGS (eugenol synthase). (**B**) Screening enzyme candidates in cell lysates with 3 mM substrate. No enzyme candidates were selected from the paired 4CL + CCR screen fed ferulate. PsCAD (*Pseudomonas* strain HR199) was selected to convert coniferaldehyde to coniferol. PhCFAT (*Petunia hybrida*) and OcEGS1 (*Ocimum basilicum*) were selected for converting coniferol to eugenol. (**C**) Screening coniferol pathway constructs in DH5α cells fed 3 mM ferulate. After 24 hr, SBC009968 was the most productive plasmid. (**D**) Screening eugenol pathway constructs in DH5α cells fed 3 mM coniferol. After 24 hr, SBC009876 was the most productive plasmid. (**E**) Best performing plasmid constructs. Functional constructs for converting tyrosine into ferulate were not identified in this study. PhCCR1 (CCR from *Petunia hybrida*), Gm4CL3 (4CL from *Glycine max*). (**F**) *In vivo* production of coniferol and eugenol. DH5α (*ΔtyrR ΔpheLA*) strains with the indicated plasmids were fed 3 mM ferulate or coniferol and assayed after 24 hr.


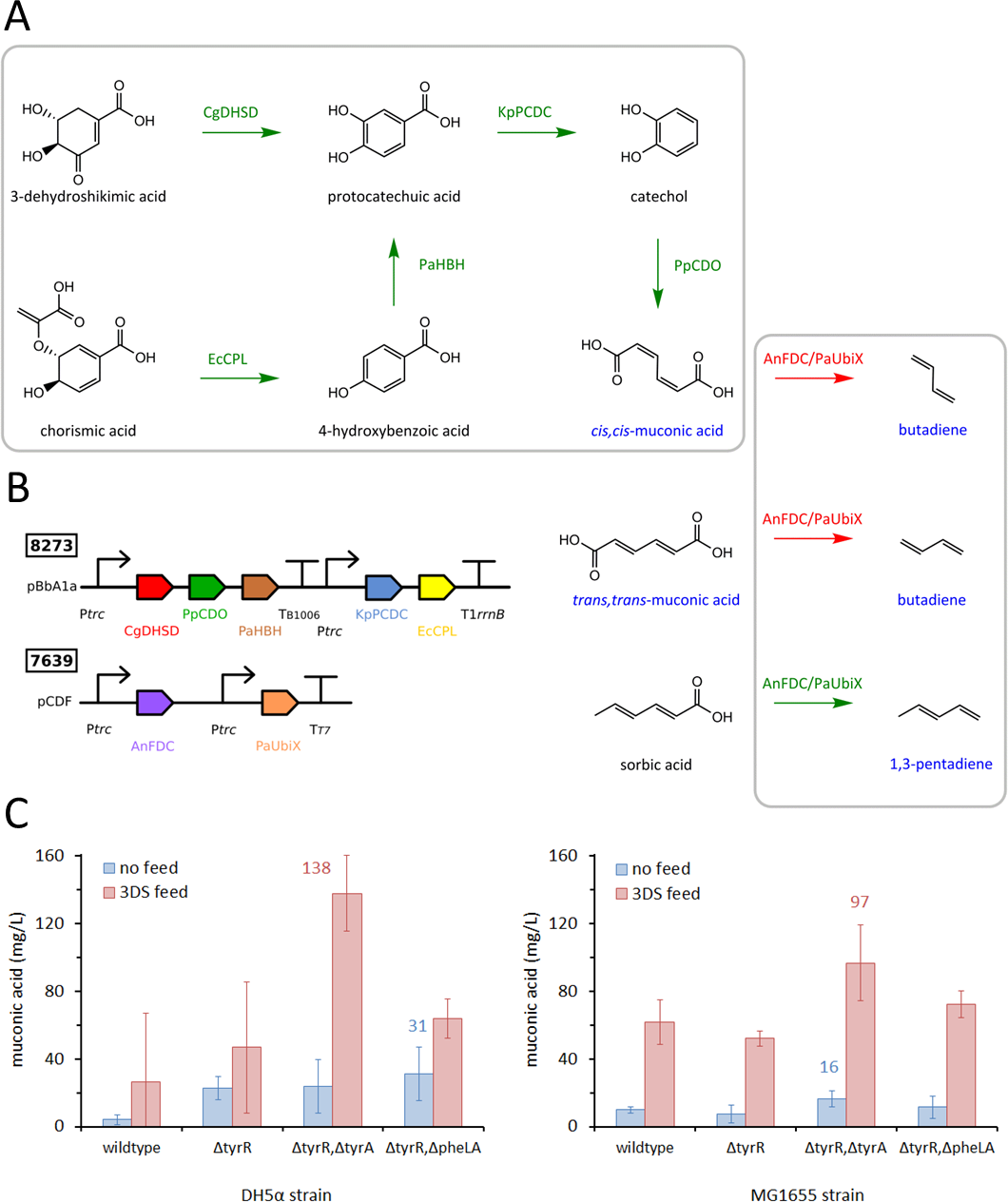


**Figure S5**: (**A**) Pathways from shikimate pathway intermediates to *cis,cis*-muconic acid and diene targets. Green arrows indicate experimentally verified activity (this study), red arrows indicate no observed activity. MS quantification methods were developed for compounds named in blue. Enzyme abbreviations: CgDHSD (3-dehydroshikimate dehydratase from *Corynebacterium glutamicum*); EcCPL (chorismate pyruvate-lyase from *Escherichia coli*); PaHBH (p-hydroxybenzoate hydroxylase from *Pseudomonas aeruginosa*); KpPCDC (protocatechuate decarboxylase from *Klebsiella pneumoniae*); PpCDO (catechol 1,2-dioxygenase from *Pseudomonas putida*); AnFDC (ferulic acid decarboxylase from *Aspergillus niger*); and PaUbiX (flavin prenyltransferase from *Pseudomonas aeruginosa*). (**B**) Plasmid constructs. SBC008273 carries the 5-gene *cis,cis*-muconic acid pathway, whilst SBC007639 carries the AnFDC and PaUbiX genes. DH5α cells transformed with SBC007639 converted sorbic acid (3 mM feed) into 1,3-pentadiene (9.2 mg/L), but no detectable butadiene was produced from either *cis,cis*- or *trans,trans*-muconic acid substrates. (**C**) *In vivo* production of muconic acid. DH5α and MG1655 wildtype strains, and mutant strains engineered to increase flux through the shikimate pathway, were transformed with SBC008273 and assayed for muconic acid production after 24 hr both with and with 3 mM 3-dehydroshikimic acid (3DS) feed.


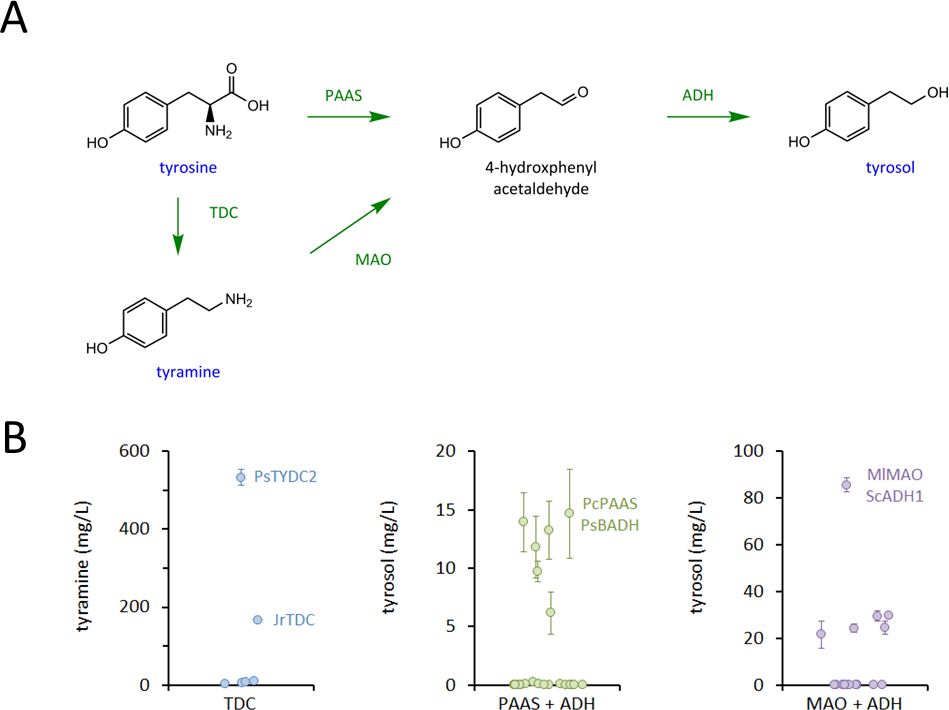


**Figure S6**: (**A**) Branched pathway from tyrosine to tyrosol. Green arrows indicate experimentally verified activity (this study). MS quantification methods were developed for compounds named in blue. Enzyme abbreviations: PAAS (phenylacetaldehyde synthase); TDC (tyrosine decarboxylase); MAO (monoamine oxidase); and ADH (aryl-alcohol dehydrogenase). (**B**) Screening enzyme candidates in cell lysates with 3 mM substrate. PsTYDC2 (*Papaver somniferum*) was selected to convert tyrosine to tyramine. PcPAAS (*Petroselinum crispum*) and PsBADH (*Pseudomonas putida*) were selected for converting tyrosine to tyrosol. MlMAO (*Micrococcus luteus*) and ScADH1 (*Saccharomyces cerevisiae*) were selected for converting tyramine to tyrosol. However, since these enzymes have been described previously for the production of tyrosol in *E. coli* (Chung et al., 2017), we abandoned further plans to design and construct tyrosol pathway libraries.


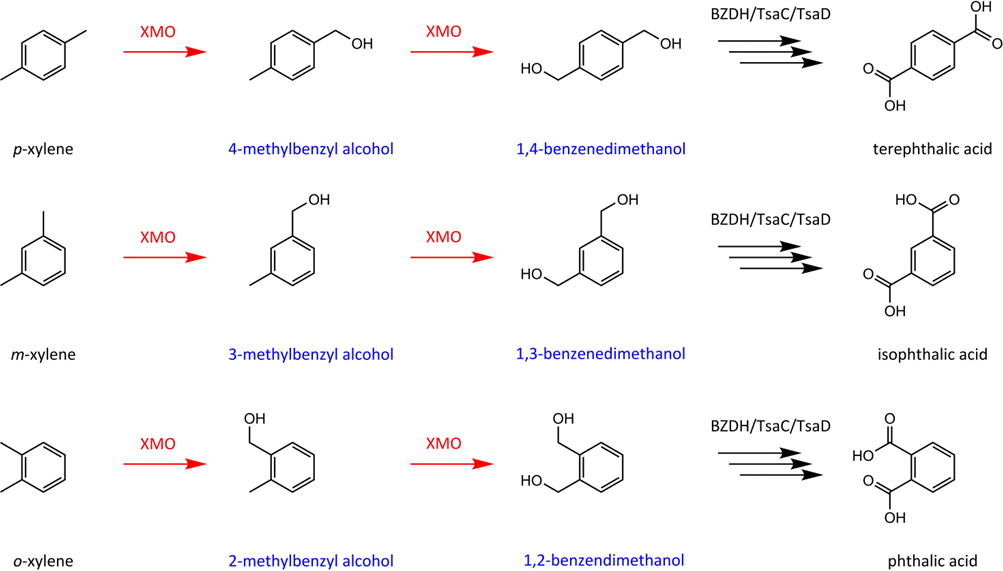


**Figure S7**: Putative pathways from xylene substrates to benzene dicarboxylate targets. Red arrows indicate no experimentally verified activity (this study). MS quantification methods were developed for compounds named in blue. Enzyme abbreviations: XMO (xylene monooxygenase); BZDH (benzaldehyde dehydrogenase); TsaC (4-formylbenzenesulfonate dehydrogenase); and TsaD (4-(hydroxymethyl) benzenesulfonate dehydrogenase). A novel XMO (from *Sphingomonas strain ASU1*, Bramucci et al., 2001) was tested alongside an XMO from the literature (*Pseudomonas putida*, Luo & Lee, 2017) for the ability to convert ortho-, meta- and para-xylene substrates into their respective methylbenzyl alcohol or benzenedimethanol products. Dual monoxygenase activity would shorten the published terephthalic acid pathway (Luo & Lee, 2017), by removing the need for the heterodimeric enzyme TsaMB (Toluene-4-sulfonate monooxygenase), and might allow for production of the alternative targets phthalic acid and isophthalic acid. However we detected no *in vitro* activity from the two XMO candidates with any of the xylene substrates, and so the production of benzenedicarboxylate targets in this study was abandoned.


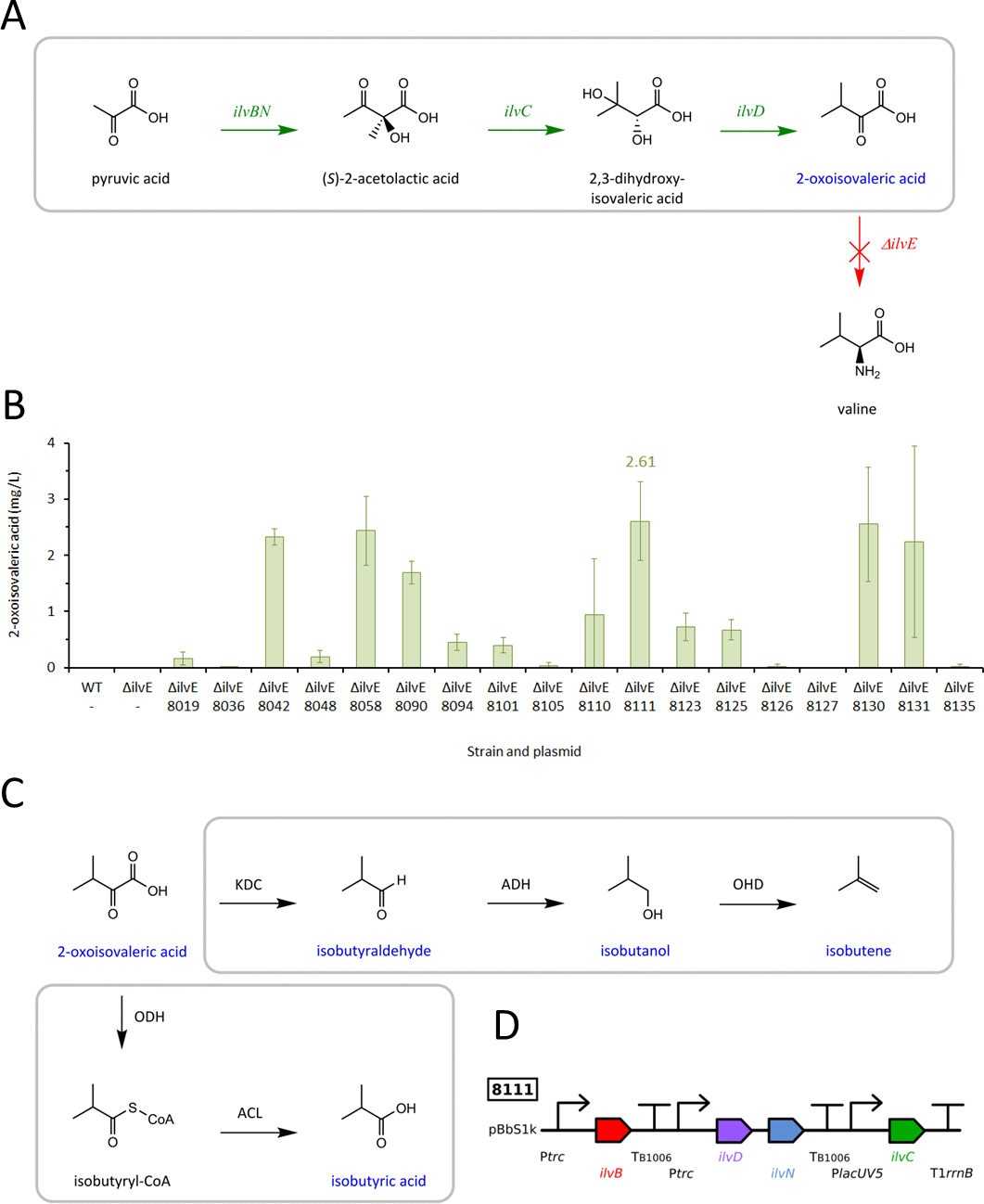


**Figure S8**: (**A**) Metabolic engineering of *E. coli* cells to increase flux down the valine pathway towards 2-oxoisovaleric acid. The three enzymes (four genes) shown in green were over-expressed from plasmid constructs, whilst the gene for the final step (*ilvE*) was deleted from the host genome. Genes encode the following enzymes: *ilvBN* (acetolactate synthase isozyme 1, large & small subunits); *ilvC* (ketol-acid reductoisomerase); and *ilvD* (dihydroxy-acid dehydratase). (**B**) Screening 2-oxoisovalerate pathway constructs in DH5α mutants (*ΔilvE*) growing in TBP media with 0.4% glycerol. After 24 hr, SBC008111 was the most productive plasmid (2.61 mg/L) in the *ΔilvE* host. 2-oxoisovalerate was below the limit of detection for both the wildtype (WT) strain and the *ΔilvE* mutant when no pathway construct was present. (**C**) Pathways from 2-oxoisovalerate to isobutene and isobutyric acid. MS quantification methods were developed for compounds named in blue. Enzyme abbreviations: KDC (α-ketoacid decarboxylase); ADH (alcohol dehydrogenase); OHD (oleate hydratase); ODH (2-oxoisovalerate dehydrogenase); ACL (acetate-CoA ligase). A 24 member DoE plasmid library for the 2-step pathway to isobutyric acid was designed, constructed and sequence-verified. However, suitable MS methods for accurate quantification of target compounds were not developed during the timeframe of this study, and so screening of enzyme candidates *in vitro* or plasmid libraries *in vivo* could not proceed. (**D**) Best performing 2-oxoisovalerate pathway construct (SBC008111).


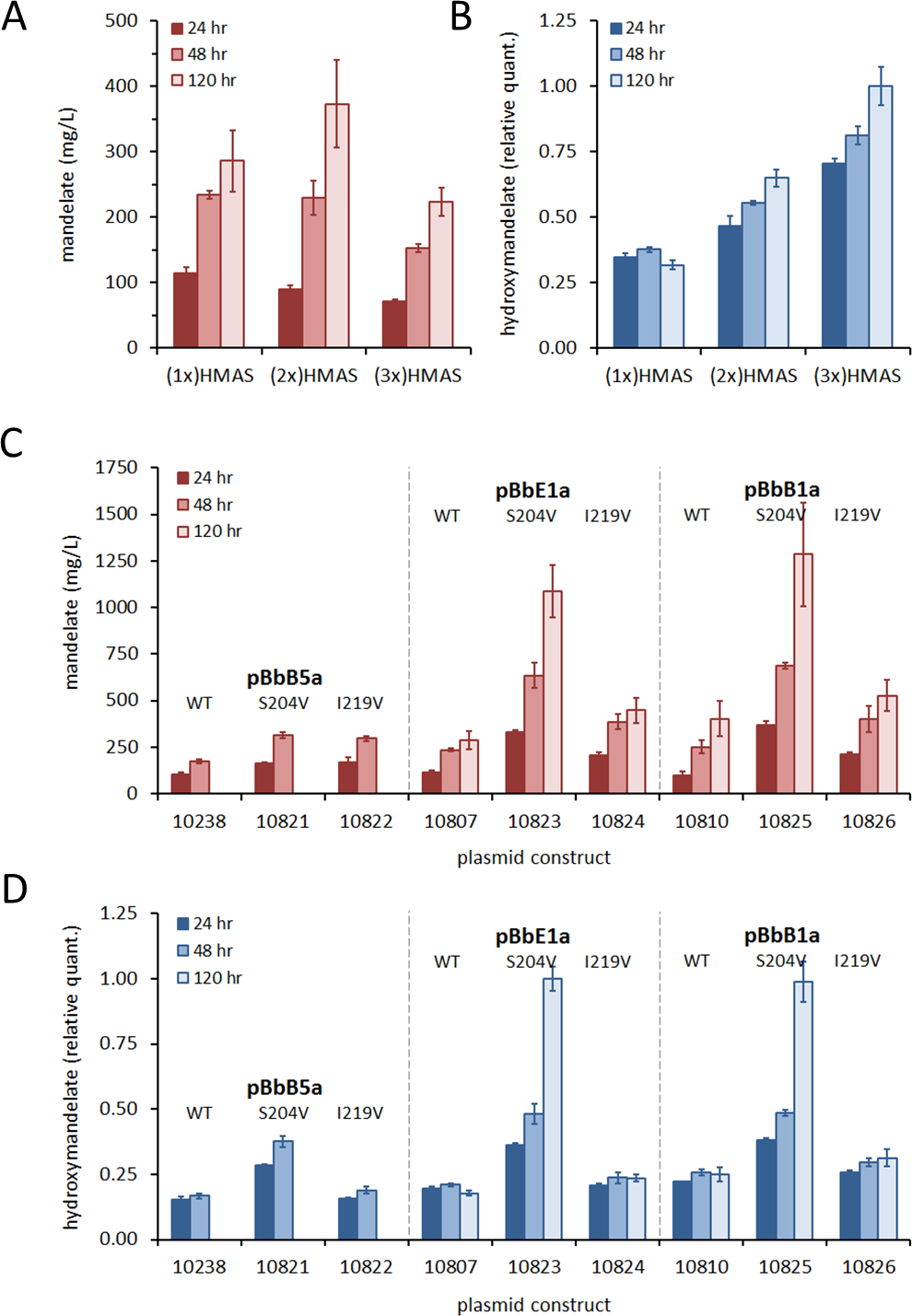


**Figure S9**: Optimization of plasmid constructs for production of mandelic/hydroxymandelic acid. (**A**) Gene dosage assay. Gene synthesis of SyHMAS was performed three times (using different redundant codons to reduce sequence homology). One, two or three copies of SyHMAS were then cloned into the pBbE1a vector then tested for mandelic acid production (24, 48, and 120 hr timepoints) in *E. coli* DH5α cells grown at 1 ml scale in TBP media supplemented with 0.4% glycerol. No significant differences in mandelic acid titers were observed between the three constructs. (**B**) The same cultures described in (A) were screened for relative production of hydroxymandelate. For this analyte there was a strong correlation between gene copy number and final titer. (**C**) SyHMAS mutants S204V ((*R*)-selective) and I219V (enhanced titer) were sub-cloned from the pBbB5a vector into pBbE1a and pBbB1a. *E. coli* DH5α cells were transformed with each plasmid and grown at 1 ml scale in TBP media supplemented with 0.4% glycerol. Mandelic acid titers were measured at 24, 48 and 120 hr timepoints. The pBbB1a plasmids were most productive, yielding 402 mg/L (WT), 527 mg/L (S204V) and 1285 mg/L (I219V) of mandelic acid. (**D**) The same cultures described in (C) were screened for relative production of hydroxymandelate (prior to optimization of an absolute quantification method).


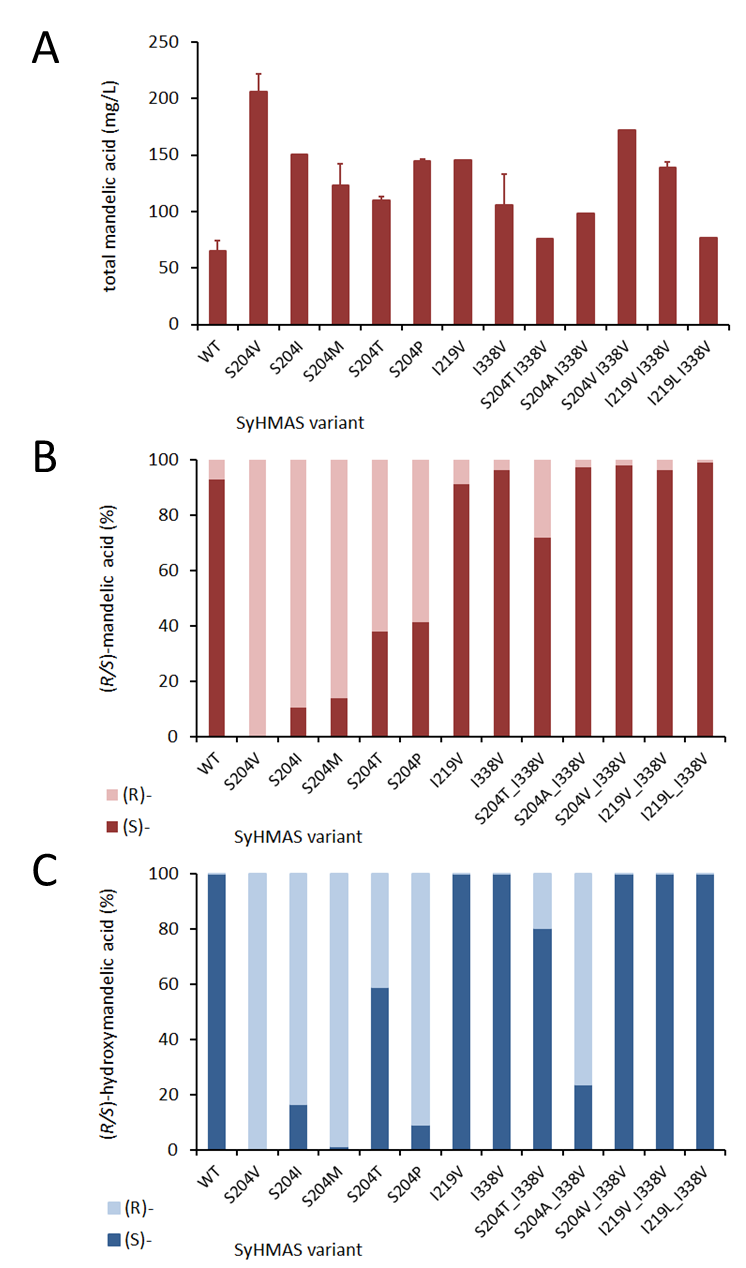


**Figure S10**: Mutagenesis of hydroxymandelic acid synthase. (**A**) SyHMAS was targeted for mutagenesis at three residues (S204, I219, I338) surrounding the phenyl group of the mandelic/hydroxymandelic acid substrates (Fig. 6B). PCR using the NNK codon generated products covering all potential amino acid substitutions at each target residue. PCR products were cloned into the pBbB5a expression vector then transformed into *E. coli* DH5α cells. Colonies were picked into TBP media with 0.4% glycerol and mandelic acid titers measured 24 hr after IPTG induction. Clones with enhanced titer compared to the wildtype were selected for DNA sequencing and chiral analysis. The chart shows mandelic acid titers for sequenced single and double mutants. (**B**) Chiral analysis of mandelic acid produced by selected SyHMAS mutants above. The ratio of (*R*)- and (*S*)-enantiomers is presented. (**C**) Chiral analysis of hydroxymandelic acid produced by selected SyHMAS mutants above. The ratio of (*R*)- and (*S*)-enantiomers is presented. From this data, S204V was the mutant with the most enhanced mandelic acid titer (3.15x wildtype). The S204V mutant also produced enantiopure (*R*)-mandelic/hydroxymandelic acid. The I219L_I338V double mutant produced the purest (*S*)-mandelic/hydroxymandelic acid (>99%).


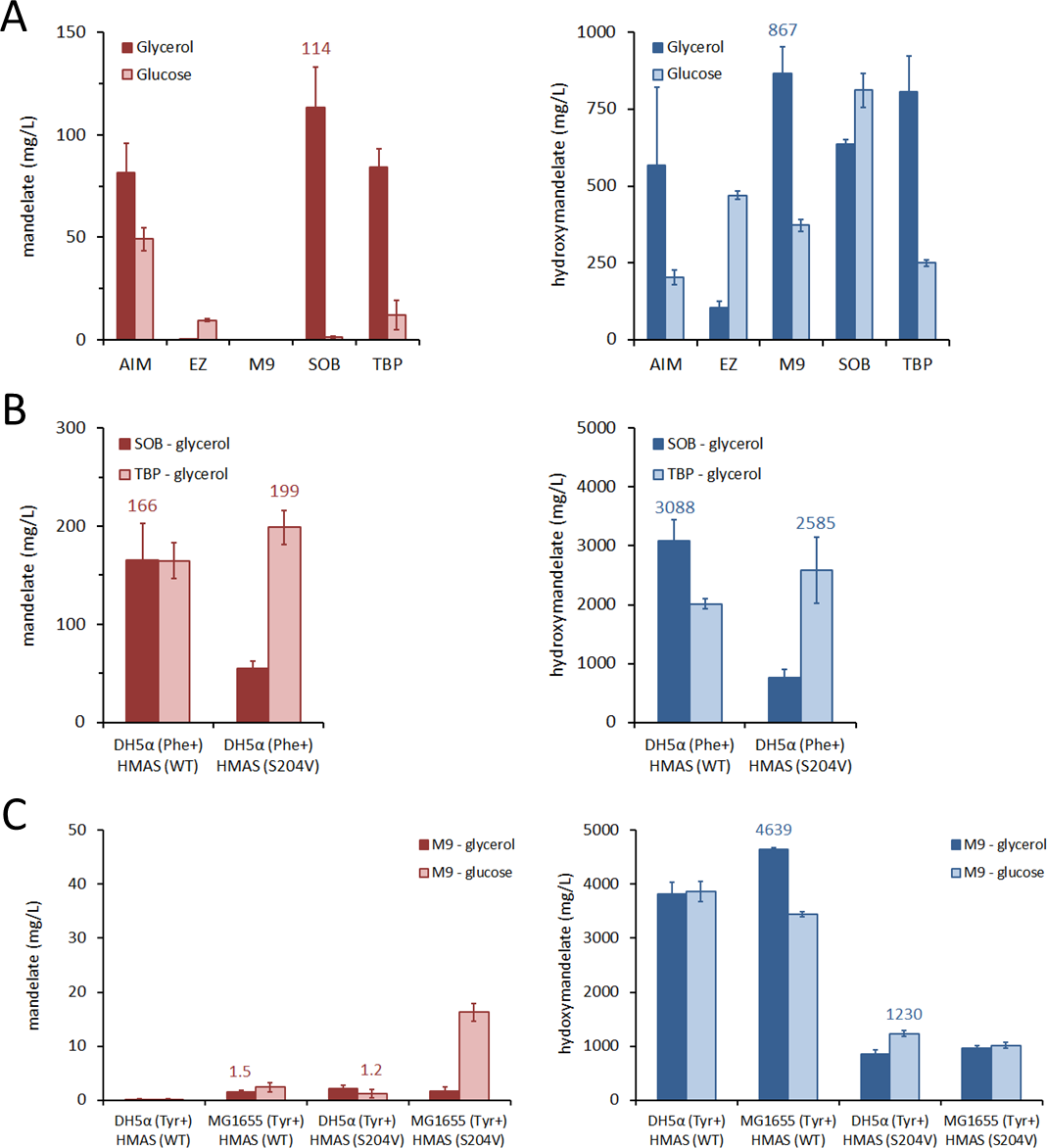


**Figure S11**: Optimization of *E.coli* growth media for production of mandelic/hydroxymandelic acid. (**A**) First round media screen. Wildtype *E. coli* DH5α cells were transformed with the pBbB5a-SyHMAS plasmid (SBC0010238) and grown at 1 ml scale in the indicated media supplemented with 0.4% glycerol or glucose. Mandelic acid (*red bars*) and hydroxymandelic acid (*blue bars*) titers were measured after 24 hr. From this screen, SOB-glycerol and M9-glycerol were identified as the best media for mandelic acid and hydroxymandelic acid production, respectively. (**B**) Second round media screen in the Phe+ engineered host strain. The phenylalanine boost construct (SBC008376; Fig. S2) was integrated at the *lacZ* genome locus of the DH5α Δ*tyrR* Δ*tyrA* double knockout strain, to create DH5α(Phe+). This strain was transformed with pBbB5a-SyHMAS(WT) or pBbB5a-SyHMAS(S204V) plasmids and grown at 1 ml scale in SOB or TBP media supplemented with 0.4% glycerol. Mandelic acid (*red bars*) and hydroxymandelic acid (*blue bars*) titers were measured after 24 hr. From this screen, TBP-glycerol was chosen as the best media for (*S*)-mandelic acid production (HMAS(WT)) and SOB-glycerol chosen for (*R*)-mandelic acid production (HMAS(S204V)). (**C**) Second round media screen in Tyr+ engineered host strains. The tyrosine boost construct (SBC005753; Fig. S2) was integrated at the *lacZ* genome locus of the DH5α and MG1655 Δ*tyrR* Δ*pheLA* double knockout strains, to create DH5α(Tyr+) and MG1655(Tyr+). These strains were transformed with pBbB5a-SyHMAS(WT) or pBbB5a-SyHMAS(S204V) plasmids and grown at 1 ml scale in M9 media supplemented with 0.4% glycerol or glucose, as indicated. Mandelic acid (*red bars*) and hydroxymandelic acid (*blue bars*) titers were measured after 24 hr. From this screen, M9-glycerol was chosen as the best media for (*S*)-hydroxymandelic acid production (HMAS(WT)) and M9-glucose chosen for (*R*)-mandelic acid production (HMAS(S204V)).
